# A compilation of ticks and tick-borne pathogen distributions in seven countries within North and West Africa from 1901 to 2022: a systematic literature review

**DOI:** 10.1186/s13071-025-07153-8

**Published:** 2025-12-04

**Authors:** Graham Matulis, Alexander M. Potter, Abigail A. Lilak, David B. Pecor, Dustin Rodriguez, Liberty Wood, Regina Cerimele, Kenna Stone, Nora G. Cleary, Kathleen Butler, Yvonne-Marie Linton, Michael E. von Fricken

**Affiliations:** 1 One Health Center of Excellence, College of Public Health and Health Professions, University of Florida, Gainesville, FL USA; 2 Emerging Pathogens Institute, University of Florida, Gainesville, FL USA; 3 Entomology Branch, Walter Reed Army Institute of Research (WRAIR), Silver Spring, MD USA; 4 James Madison University, Harrisonburg, VA USA; 5 Columbia University, New York, NY USA; 6 George Mason University, Fairfax, VA USA; 7 Natural History Research Experiences Internship Program, Smithsonian Institution - National Museum of Natural History, Washington, DC USA; 8 Department of Entomology, Smithsonian Institution - National Museum of Natural History, Washington, DC USA

**Keywords:** Ticks, Tick-borne pathogens, Algeria, Morocco, Niger, Nigeria, Senegal, Sierra Leone, Tunisia, Systematic review

## Abstract

**Background:**

Ticks continue to represent a significant threat to human and animal health worldwide. In the absence of comprehensive databases that compile the current published records of tick diversity and distributions, the information from hundreds of articles remains in isolation, incapable of being easily incorporated into surveillance gap analyses and tick-borne pathogen risk modeling. Here, our group systematically reviews and extracts data from published studies that detail ticks collected within seven target countries in North and West Africa.

**Methods:**

We conducted a systematic literature review of documented tick distribution and tick-borne pathogen associations published from Algeria, Morocco, Nigeria, Niger, Senegal, Sierra Leone, and Tunisia between 1901 and 2022. Three databases were screened, and after a priori exclusion criteria, 372 articles were deemed eligible for review and underwent data extraction. Relevant information pertaining to ticks and tick-borne pathogens was collected and compiled into a dataset allowing for the articles to be georeferenced.

**Results:**

The articles included within the final dataset reported 74 hard tick species and 30 soft tick species throughout the seven target countries. Species of the genus *Rhipicephalus* dominated (*n* = 24 species), followed by *Hyalomma* (*n* = 14)*, Ornithodoros* (*n* = 14), and *Haemaphysalis* (*n* = 13). Almost 80% of collection events involved ticks removed from nonhuman vertebrates. Approximately 12% of collection events included evidence of an associated microbial species, including 17 bacterial genera, 3 eukaryotic genera, and 16 viruses. Through mapping of the collection events, we begin to characterize surveillance gaps within each country.

**Conclusions:**

This systematic review highlights notable surveillance gaps that vary by country and region. While Morocco, Nigeria, and Senegal report numerous studies concerning tick diversity, certain regions appear oversampled, while reports from other regions are largely absent. Information on the ticks of Sierra Leone and Niger remains largely uncharacterized. Across all target countries, reports are heavily biased toward sampling and testing ticks collected from domestic animals. Future tick surveillance efforts need to include sampling and testing of ticks collected from the environment and wildlife species to obtain a more complete assessment of tick distribution and tick-borne pathogen ecology.

**Graphical Abstract:**

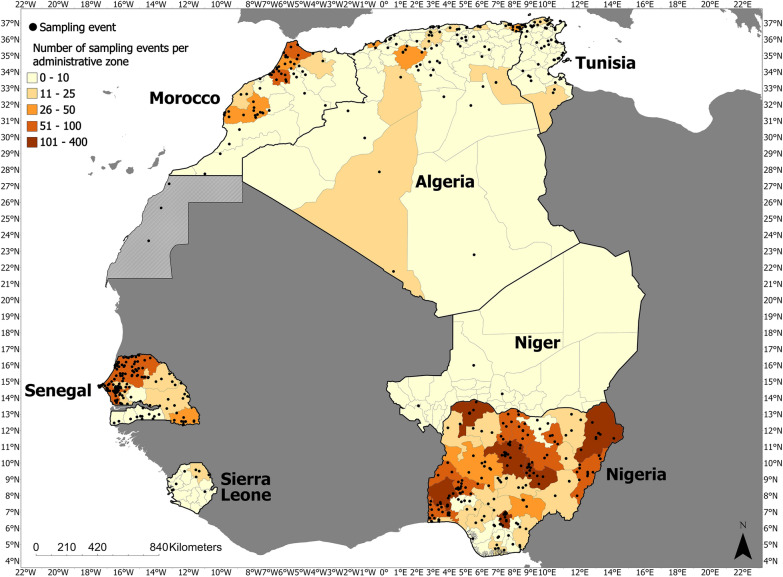

**Supplementary Information:**

The online version contains supplementary material available at 10.1186/s13071-025-07153-8.

## Background

Within certain areas of North and West Africa, up to 50% of the population tend livestock, which often represents their primary source of income and food security [1, 2]. This is especially common in periods of low crop yield, or in regions with little arable land [1, 2]. Diverse livestock production systems are practiced within these regions, including pastoral, agropastoral, and intensive and extensive production systems, each of which have differing levels of exposure to tick-infested habitats [2]. While there is a trend toward the intensification of production systems in North and West Africa, tick-borne pathogens (TBPs) continue to affect productivity gains and efficiency of production [2]. Within the West African region, an estimated two-thirds of traded animals are transported across international borders [1]. This mass movement of animals has already been associated with the introduction of the invasive cattle tick *Rhipicephalus microplus* across West Africa in recent years [3, 4]. This commerce-driven spread of tick species is co-occurring with changes in climate, which further facilitates the geographic expansion of invasive tick vectors and their associated pathogens into native populations, increasing the risk of zoonotic spillover [5].

There are major gaps in our understanding of tick distributions and TBPs in North and West Africa. Previous reviews concerning ticks and TBPs in the Middle East and North Africa (MENA) and West African countries [6, 7] did not capture locality datasets of tick and/or TBP observations. This lack of geospatial data largely precludes analyses using geographic information systems (GIS), where data could be correlated with real-time diagnostic tools and remote sensing observations.

To best characterize the threat posed by TBPs, we must consider how these diseases interact with humans, animals, and the environment. To elucidate connections between these elements, high-quality data detailing repeatable observations are needed. This is particularly important for regions such as North and West Africa, where the impacts of TBPs can have devastating consequences for both human and animal health.

Following a similar process that our team previously used to systematically assess the published records of ticks within countries of East and Central Africa [8, 9], here we present the results of an expansive literature review to better characterize the diversity and distribution of ticks and TBP in seven countries in North and West Africa: Algeria, Morocco, Niger, Nigeria, Sierra Leone, Senegal, and Tunisia. Outputs of this review enhance the knowledge and expose the gaps in knowledge of ticks and tick-borne pathogens in North and West Africa, which can be used to inform future surveillance efforts and better characterize TBP risk for humans and animals within the region.

## Methods

To acquire articles, three databases (PubMed, Scopus, and Web of Science) were utilized with 19 MeSH search terms using Boolean operators as outlined in Table 1. Captured articles were published between January 1901 through May 2022 and included articles written both in English and in French. All French articles were translated to English prior to data extraction. Cited references in retrieved articles were leveraged to identify additional peer-reviewed publications that may not have been captured with the initial search terms. All additional articles were reviewed using the same methodology as described.

**Table 1:** Search terms applied in the four search engines used for systematic review

PubMed	Scopus, Web of Science
("Tick-Borne Diseases"[Mesh] OR "Rickettsia"[Mesh] OR "Anaplasmataceae"[Mesh] OR "Borrelia"[Mesh] OR "Babesia"[Mesh] OR “tick-borne zoonosis” OR "tick-borne zoonotic disease" OR “Seroepidemiologic Studies"[Mesh] OR "Hemorrhagic Fever virus, Crimean-Congo"[Mesh] OR "Ticks"[Mesh] OR “*Amblyomma*” OR “*Dermacentor*” OR “*Haemaphysalis*” OR “*Hyalomma*” OR “*Ixodes*” OR “*Margaropus*” OR “*Rhipicephalus*” OR "*Ornithodoros*" OR "*Argas*") AND (*country*)	("Tick-Borne Diseases" OR "Rickettsia" OR "Anaplasmataceae" OR "Borrelia" OR "Babesia" OR "tick-borne zoonosis" OR "tick-borne zoonotic disease" OR "Seroepidemiologic Studies" OR "Crimean-Congo Hemorrhagic Fever virus" OR "Ticks" OR "*Amblyomma*" OR "*Dermacentor*" OR "*Haemaphysalis*" OR "*Hyalomma*" OR "*Ixodes*" OR "*Margaropus*" OR "*Rhipicephalus*" OR "*Ornithodoros*" OR "*Argas*") AND (*country*)

### Eligibility criteria

Captured articles underwent a standardized a priori inclusion/exclusion assessment. Inclusion criteria included those reporting (1) original research in the target West and North African countries, (2) studies focusing on ticks and/or TBPs, and (3) articles with georeferenced localities, or sufficiently detailed survey location data that allowed for retrospective georeferencing. Exclusion criteria included (1) studies conducted outside our countries of interest, (2) studies with insufficient data detail for retrospective georeferencing, and (3) studies not based on field observations (i.e., laboratory studies working with tick colonies). The breakdown of articles by country can be found in Table 2 as well as Additional File 1.

**Table 2:** Country breakdown of article inclusion at each criteria stage. Number represents remaining number of articles at each stage. Nine final articles included focused on multiple countries (making a total of 373 individual papers). *The inaccessible articles were those unrecoverable through databases, online searches, and interlibrary loan requests

Database	Algeria	Morocco	Niger	Nigeria	Senegal	Sierra Leone	Tunisia	Total
PubMed	170	190	154	672	298	32	318	1834
WOS	371	383	191	888	505	50	508	2896
Scopus	170	155	85	572	261	27	252	1522
Total	711	728	430	2132	1064	109	1078	6252
Removed duplicates	425	245	314	1081	801	60	619	3545
Removed on the basis of title	231	219	102	483	612	21	477	2145
Removed on the basis of abstract	148	135	40	273	165	13	317	1090
Removed on the basis of article	87	45	6	151	94	7	76	466
Inaccessible*	24	10	0	1	38	0	39	112
Additional from secondary sourcing	0	1	0	53	0	1	0	55

### Data management

The process of assessing individual article eligibility was recorded for each country using Microsoft Excel spreadsheets. All database results were compiled by country then sorted according to Preferred Reporting Items for Systematic reviews and Meta-Analyses (PRISMA) guidelines. Each stage was documented as a separate sheet showing the process of elimination throughout the title, abstract, and full review stages. First, results from each database were combined into a single list and duplicate articles were removed. Next, titles were reviewed and then abstracts, and finally a full article review was conducted. If any of the articles did not meet the inclusion criteria at any stage, they were removed. Articles that met all inclusion criteria after full review were compiled for data mining.

### Data mining

Data were extracted using a customized data schema outlined in Lilak et al. (2024), which contains 93 fields of potential information to document collection events [8]. All relevant fields were captured for each collection event record within every article. The data schema included information in relation to the tick identification, environmental observations, collection method, locality, specimen count, host association, and pathogen detection methods and/or results. All collection events were recorded to the most specific locality and separated by time and date if the information was provided. If a single collection event captured multiple species of ticks or sampled multiple hosts, a separate entry was made for each species or sampled host species. The dataset including the articles, Global Positioning System (GPS) coordinates, and relevant fields can be found in Additional File 2.

### Georeferencing

Locality descriptions of tick collection sites captured during data mining were separated by those that included geographic coordinates for tick collections and those that reported named locations only. Articles with published GPS coordinates were converted into decimal degrees, and spatial uncertainty was measured using the point-radius method [10]. Collection events with only named locations were searched within an online gazetteer to obtain a locality centroid (GeoNames.org). Uncertainty was measured by calculating the furthest distance between the centroid and the respective boundary of the area border using Google Maps. Location was recorded by country, state/province, county, and most specific locality level. If the location was unable to be determined, the next highest administrative unit was then used to obtain a centroid. All the geographic data visualizations were created in ArcGIS Pro 2.7.3 (ESRI Inc.), and country shapefiles were obtained from the Database of Global Administrative Areas (GADM, version 4.0).

## Results

**Fig. 1:**
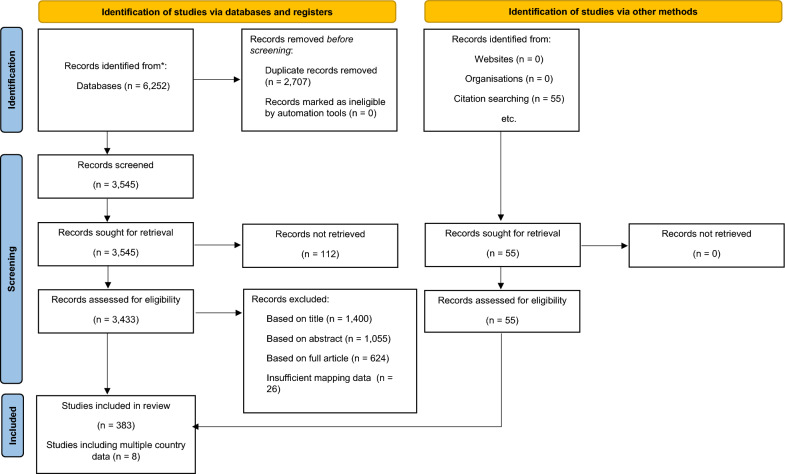
Overall PRISMA

Through the data collection process, 6252 articles were recovered for the seven selected countries through the three databases (PubMed = 1834, Scopus = 1522, WoS = 2896). After all captured articles were assessed using the inclusion/exclusion criteria, 372 unique articles were mined and data georeferenced manually. A breakdown of the overall article assessment process can be found in Fig. 1. Across all seven countries, there were 112 articles identified through the databases that our group was unable to locate using interlibrary loans at our respective institutions. The articles included in our dataset for each country include: Algeria [11–66], Morocco [25, 62–65, 67–93], Niger [94–99], Nigeria [64, 100–290], Senegal [64, 74, 280, 291–341], Sierra Leone [167, 342–347], and Tunisia [64–66, 348–381] (Additional File 3). A visual representation of article publication trends over time by country is included as Additional File 4.

### Study results

Across the seven target countries, a total of 74 hard tick species, representing seven genera, and 21 soft tick species, representing three genera, were reported. A complete list of tick species reported by country can be found in Additional File 5. *Rhipicephalus* ticks had the greatest number of unique species reported in the North and West African target countries (*n* = 24 species), followed by *Hyalomma* (*n* = 14 species), *Ornithodoros* (*n* = 14 species), and *Haemaphysalis* (*n* = 13 species). Ticks attached or crawling on vertebrate hosts accounted for 79.9% of the reports, while 7.5% were collected from the environment (e.g., by flagging or dragging); only eight records detailed a tick attached or crawling on a human.

Twelve percent of all extracted tick records were associated with microbial species. In all, across all seven countries, 17 bacterial genera, 3 protozoal genera, and 16 viruses were found (Table 3). *Rickettsia*, *Borrelia*, and *Anaplasma* were the bacterial genera with the greatest number of reported unique species, while *Babesia* was the most diverse of the protozoal genera.

**Table 3:** Comprehensive list of all pathogens identified in ticks from this s﻿﻿tudy

**Bacteria**	*R. monacensis*
*Anaplasma* spp.	*R. raoultii*
*A. bovis*	*R. rickettsii*
*A. centrale*	*R. sibrica mongolitimonae*
*A. marginale*	*R. slovaca*
*A. ovis*	*Rickettsiella* spp.
*A. phagocytophilum*	*Staphylococcus aureus*
*A. platys*	*Streptococcus pyogenes*
Anaplasmataceae	*Streptomyces albus*
*Bacillus* spp.	**Protozoa**
*B. subtilis*	*Babesia* spp.
*Bartonella* spp.	*B. bigemina*
*B. elizabethae*	*B. bovis*
*B. senegalensis*	*B. caballi*
*Borrelia* spp.	*B. canis rossi*
*B. anserina*	*B. divergens*
*B. burgdorferi*	*B. equi*
*B.* cf. *turicatae*	*B. occultans*
*B. crocidurae*	*Hemolivia mauritanica*
*B. garinii*	*Theileria* spp.
*B. hispanica*	*T. annulata*
*Candidatus* B. kalaharica	*T. buffeli*
*Coxiella* spp.	*T. equi*
*C. burnetii*	*T. mutans*
*Ehrlichia* spp.	*T. orientalis*
*E. canis*	*Trypanosoma evansi*
*E. chaffeensis*	**Viruses**
*E. ewingii*	African swine fever virus (ASFV)
*E. ruminantium*	Bandia virus (BDAV)
*Candidatus* E. urmitei	Bhanja virus (BHAV)
*Escherichia coli*	Crimean–Congo hemorrhagic fever virus (CCHFV)
*Francisella* spp.	Dugbe virus (DUGV)
*Occidentia massiliensis* gen. nov., sp. nov	Essaouria virus (ESSV)
*Proteus mirabilis*	Jos virus (JOSV)
*Pseudomonas aeruginosa*	Kala Iris virus (KIRV)
*Rickettsia* spp.	Ngoye virus (NGOV)
*R. africae*	Nyamanini virus (NYMV)
*R. aeschlimannii*	Quaranfil virus (QRFV)
*R. conorii*	Soldado virus (SOLV)
*R. felis*	Thogotovirus (THOV)
*R. helvetica*	Tick-borne encephalitis virus (TBEV)
*R. massiliae*	Tunis virus (TUNV)
*Candidatus* R. mauretanica	Wad Medani virus (WMV)

**Fig. 2:**
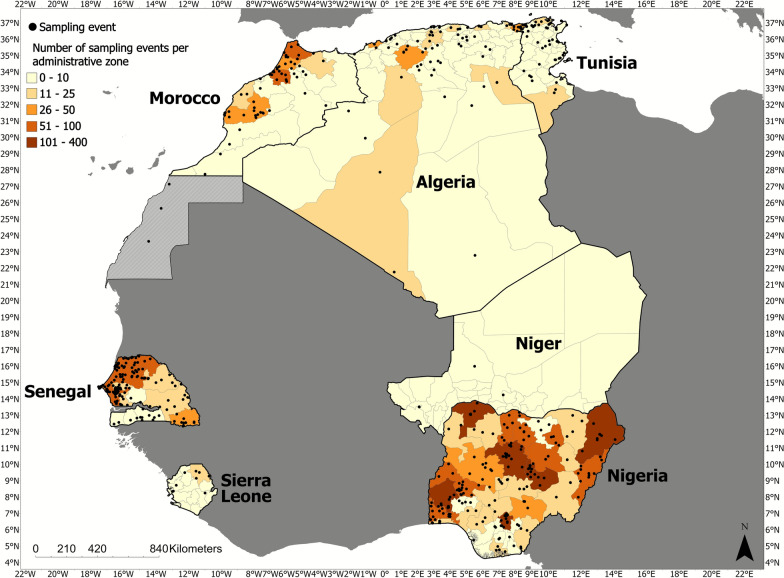
Map of tick collection events in target countries (1901–2022)

A map depicting the distribution of collection events across all seven countries can be found in Fig. 2. Collection events in which georeferencing was executed at country level were removed from these maps to avoid artificial overrepresentation of sampling events within the center of each country.

#### Algeria

Our initial literature search returned 425 unique articles, of which only 56 articles met the inclusion criteria and were included in the final dataset (Table 2). A total of 33 species belonging to nine tick genera were documented in records from Algeria (Additional File 5), including six hard tick genera (*Amblyomma* spp., *Dermacentor* spp., *Haemaphysalis* spp., *Hyalomma* spp., *Ixodes* spp., and *Rhipicephalus* spp.) and three soft tick genera (*Argas* spp., *Carios* spp. and *Ornithodoros* spp). The *Hyalomma* genus was most speciose (*n* = 11 species), followed by *Ornithodoros* (*n* = 7 species). The reported microbial groups include Crimean–Congo hemorrhagic fever virus (CCHFV), *Anaplasma* spp., *Babesia* spp., *Borrelia* spp., *Ehrlichia* spp., *Rickettsia* spp., and *Theileria* spp. (Additional File 6). Tick collection events were heavily biased toward host removal (76%), of which 84% were from livestock, primarily cattle (*Bos taurus*). Other host animal collections occurred from dogs (*Canis lupus*) and various reptile species.

#### Morocco

Our initial search returned 245 unique articles. After full review, 32 articles met the final inclusion requirements (Table 2). There were seven tick genera reported (Additional File 5):five hard tick genera (*Dermacentor* spp., *Haemaphysalis* spp., *Hyalomma* spp., *Ixodes* spp., and *Rhipicephalus* spp.) and two soft tick genera (*Carios* spp. and *Ornithodoros* spp.). The genus *Hyalomma* had the greatest number of unique species (*n* = 8), followed by *Ornithodoros* (*n* = 5) and *Rhipicephalus* (*n* = 3). Of the 288 unique data entries, 250 were reported as collections from animal hosts, while only 6 records were collected from the environment and the remainder included a combination of animal and environmental collections. Reported microbial groups include: *Anaplasma* spp., *B. crocidurae*, CCHFV, Kemerovo group virus, and *Rickettsia* spp. (*Rickettsia aeschlimannii*, *R. conorii*, and *Rickettsia monacensis*) (Additional File 7).

#### Niger

A total of 314 unique articles were collected using the MeSH terms for Niger. After processing these articles through the inclusion/exclusion criteria, only six articles were extracted and included in the final dataset (Table 2). Twenty-one individual tick species were observed, spanning five genera, from both hard ticks (*Amblyomma* spp., *Hyalomma* spp., and *Rhipicephalus* spp.) and soft ticks (*Argas* spp. and *Ornithodoros* spp.), with the genus having the greatest number of unique species being *Rhipicephalus* with eight species. Associated pathogens included *Ehrlichia* spp. (*Ehrlichia canis)* and *Rickettsia* spp. (*R. aeschilimannii*, *Rickettsia mongolotimonae*, and two *Rickettsia africae* subtypes) (Additional File 8). Of the 78 unique collection events, 76 involved animal hosts, primarily from domestic animals, including *B. taurus*, accounting for 34% of collection events, followed by *Capra hircus* (9%) and *Ovis aries* (5%).

#### Nigeria

Using the predetermined search criteria, 1081 articles were captured from the three separate databases. After assessing these articles for title relevance, abstract relevance, and article relevance, 192 articles were extracted for Nigeria, including 53 articles that were obtained through searching the reference lists of extracted articles (Table 2). These articles reported evidence of both hard tick genera (*Amblyomma*, *Dermacentor*, *Haemaphysalis*, *Hyalomma*, *Ixodes*, *Margaropus*, and *Rhipicephalus*) and soft tick genera (*Argas* and *Ornithodoros*) (Additional File 5). *Rhipicephalus* was the tick genus with the most unique species (*n* = 18), followed by *Amblyomma* (*n* = 12) and *Hyalomma* (*n* = 1). A majority (91.46%) of collection events represented instances in which a tick was collected off an animal. Domestic animals (cattle, dogs, sheep, and goats) were the most frequently sampled hosts (91.03% of entries associated with an animal), although wildlife animals were also sampled, with a particular emphasis on rodents.

Biosurveillance efforts reported the following tick-borne pathogens in Nigeria: *Anaplasma* spp., *Babesia* spp., *Bartonella* spp., *Borrelia* spp., *Coxiella* spp., Crimean–Congo hemorrhagic fever virus, *Ehrlichia* spp., *Rickettsia* spp., and *Theileria* spp. (Additional File 9). Of note, 90.5% of infected ticks were collected off animals.

#### Senegal

Initial searches recovered 801 unique articles from Senegal, with data eventually being extracted from 53 articles (Table 2). Ticks that were reported represented four hard tick genera (*Amblyomma*, *Haemaphysalis*, *Hyalomma*, and *Rhipicephalus*) and three soft tick genera (*Argas*, *Carios*, and *Ornithodoros*), with the *Rhipicephalus* genus being most speciose (*n* = 12), followed by *Hyalomma* (*n* = 7) (Additional File 5). Associated pathogens reported in ticks collected from Senegal included CCHFV, *Borrelia* spp., *Coxiella* spp., *Ehrlichia* spp., and *Rickettsia* spp. (Additional File 10). Most collections (65.4%) were from vertebrate hosts, most of which were livestock, including *Bos* spp., *C. hircus*, and *O. aries*.

#### Sierra Leone

The search criteria resulted in 60 unique articles across all three databases for Sierra Leone. Following our inclusion and exclusion criteria, only seven articles were extracted and incorporated into the final dataset (Table 2). Tick genera that have been reported from Sierra Leone include *Amblyomma*, *Dermacentor*, *Haemaphysalis*, *Ixodes*, *Ornithodoros*, and *Rhipicephalus* (Additional File 5). Like other countries, the *Rhipicephalus* genus was represented by the greatest number of unique individual species (*n* = 5), followed by *Amblyomma* (*n* = 4) and *Haemaphysalis* (*n* = 3). The majority (89.1%) of data entries reported ticks collected off animals. Interestingly, only 56.1% of reported tick collection events from animals were collected from domestic animals. Wild Bovidae were the most common group of wildlife that were sampled. There is a country-wide surveillance gap in Sierra Leone, with no published reports on tick-borne pathogens being detected in collected ticks.

#### Tunisia

Across the three databases, 619 articles were captured for Tunisia. Once the article titles, abstracts, and text were reviewed, data from 37 articles were incorporated into the final dataset (Table 2). Four hard tick genera (*Dermacentor* spp., *Haemaphysalis* spp., *Hyalomma* spp., *Ixodes* spp., and *Rhipicephalus* spp.) and three soft tick genera (*Argas* spp., *Carios* spp., and *Ornithodoros* spp.) have been reported within the country (Additional File 5). The *Hyalomma* genus was represented by the greatest number of unique species (*n* = 7 species). Associated microbes included *Anaplasma* spp., *Borrelia* spp., *Coxiella* spp., *Francisella* spp., *Rickettsia* spp., and *Theileria* spp. (Additional File 11). Most data entries (72.9%) detailed ticks that were from a host animal, most notably domestic camels.

## Discussion

Ticks and TBPs are a major threat to human and veterinary health within North and West Africa. Besides the direct impacts that TBPs have on public health, the effects that the related diseases have on livestock can negatively impact the economy and food security of populations who heavily rely on livestock as a source of income and food. This is true for most of the target countries of this systematic literature review, which have large populations that engage in either sustenance farming or are economically reliant on livestock production. In the advent of the continued geographic expansion of tick species and the diseases they vector, there is a greater need for comprehensive databases that characterize the presence and distributions of tick species and TBPs found within these regions.

Two groups have previously published reviews on ticks and TBPs in North and West Africa [6, 7]. Perveen et al. [7] largely focused their review on a discussion of ticks collected from livestock in the MENA region, however it did not include wildlife and environmental sampling. This may explain why the tick species and TBP profiles differ for Algeria and Tunisia, when comparing this review with Perveen et al. [7]. The other systematic review, by Diarra et al. [6], focused on tick-borne pathogens within West African countries. Their inclusion criteria included papers published between 1970 and 2020, focusing primarily on specific TBP associations with humans, animals, and ticks. The work by Diarra et al. thus provides an incomplete diversity profile of tick species found within West African countries. As such, our report attempts to provide for a more expansive profile of ticks and the microbial species found within ticks for our target countries. Most significantly, our dataset also includes a spatial data component associated with each tick collection event, an aspect largely absent from the other two literature reviews. The inclusion of these spatial data will allow for more accurate descriptions of surveillance gaps, while also providing the basis for more precise tick exposure risk analyses.

Overall, scientific literature provides valuable data that can be used to more accurately characterize the diversity of tick species found throughout North and West Africa. However, surveillance coverage is not even and certain countries such as Nigeria and Senegal have vastly larger quantities of data than others. Concerning the details of the published surveillance studies, much focus has been placed on sampling livestock and domestic animals for ticks and testing these ticks for tick-borne pathogens. While this provides insight into the ticks and TBPs that may have had an economic impact on livestock industries both currently and in the past, it leaves a large information gap concerning the tick-borne pathogens that are circulating within these countries that may threaten both veterinary and public health in the future. As such, all countries targeted in this systematic literature review would benefit greatly from future studies that focus on sampling ticks from both the environment and wildlife. Testing these ticks for microbial species would be of utmost importance for a more complete characterization of TBP ecology within each country.

### Algeria

Published literature on ticks present within Algeria demonstrates a high number of *Hyalomma* spp. within the country. *Hyalomma* spp. pose a risk to both humans and animals owing to their role as vectors of *Theileria* spp. and CCHFV [382]. Additionally, although not as diversely represented as the *Hyalomma* genus, the *Rhipicephalus* spp. ticks collected within Algeria have been reported to be positive for a variety of *Anaplasma* spp. and *Theileria* spp. of veterinary concern. Algeria remains an important transit point for goods and people across the Mediterranean Basin and North Africa [383, 384]. The livestock industry is one of the major contributors to the country’s economy, and the threat that TBPs represent to the wellbeing of animals is a major concern. The impact of tick-borne pathogens on animals not only affects the animals’ health but is of great economic concern for local populations. In recent years, owing to climate and socioeconomic changes, pastoralists within the region have been forced to migrate long distances in search of new feeding areas [6]. Given that local pastoralists are moving long distances with their livestock, the risk for tick and TBP exposure is likely heightened for these populations, and such movements may continue to increase in intensity given the transportation importance of Algeria’s geographic location [6]. Given the confirmed records of CCHFV and medically relevant *Rickettsia* spp. and *Theileria* spp. within ticks in Algeria, this highlights the need for more detailed studies concerning the disease ecology of these pathogens within Algeria. While there is some diversity of tick genera represented in published records, there are areas of Algeria with few or no records, making it difficult to assess whether current records are truly representative of tick species diversity, abundance, and distribution, or an artifact of sampling convenience.

### Morocco

This systematic review demonstrates that most tick collection efforts within Morocco have focused on sampling from domestic animals in the coastal regions of the country. The records document a variety of *Hyalomma* spp., a genus that includes several important vector species. Indeed, CCHFV, *R. aeschlimannii*, *R. africae*, and *T. annulata* were all reported within *Hyalomma* ticks from Morocco. The exposure risk to these TBPs may be high for the Moroccan population given that a large proportion of the country’s workforce is involved in agriculture [385]. Additionally, within the rangelands and valleys of the Atlas Mountains, extensive pastoralism is still practiced [386]. These populations are at an enhanced occupational risk for TBP exposure, given that pastoralism often involves moving animals through environments that are densely populated with ticks. Given that Morocco’s gross domestic product (GDP) includes economic sectors that heavily rely on the continued health of livestock animals and their owners, uncharacterized ticks and TBPs can have a devastating impact on the economy and the overall public and veterinary health of the country. Indeed, our review compiled reports of *Babesia equi*, *T. annulata*, and *Anaplasma platys* in ticks from Morocco, which are all associated with disease in animals [387–389].

### Niger

The results of this systematic literature review clearly demonstrate that there are few data concerning the diversity and distribution of tick populations in Niger, as well as the pathogens present within these tick populations. This is of particular concern, given the evidence that CCHFV and *C. burnetii* have been reported from Nigerian provinces on the Niger Border [390, 391]. Niger’s economy relies on livestock production, with as much as 15% of the country’s GDP coming from the livestock sector [392], and as such, future TBP outbreaks may have severe and widespread consequences on local populations.

Importantly, we found that published surveys conducted within this country often lacked specific locality information, with only three published studies having sufficient collection information that enabled tick data to be georeferenced and mapped with confidence. Future efforts should be made to standardize tick collection data reported from Niger to ensure that local scientists uniformly conduct surveys, with an emphasis placed on recording precise locality information. The economic dependence on livestock in Niger makes human–animal interactions unavoidable; therefore, it is crucial to understand the role that ticks play as parasites and disease vectors [392].

### Nigeria

Among the countries targeted within this systematic review, Nigeria’s endemic tick population is one of the more extensively characterized, having the most publications describing the diversity and distribution of tick species found within the country. These publications have largely focused on characterizing ticks collected from domestic animals. While there are still gaps in knowledge concerning other aspects of tick-borne pathogen ecology within Nigeria, the current information available can be used to generate exposure risk heat maps and improve awareness of tick-borne pathogens within at-risk populations, such as the large proportion of the Nigerian population that maintains livestock at an individual level [393]. Such knowledge can help prevent morbidity and mortality within human populations, while also minimizing the impact it has on food security and local economies.

Despite the large amount of information available for ticks and TBPs within Nigeria, there are still notable  gaps in knowledge,  with a distinct lack of studies in the southern states of Nigeria, compared with the northern states (Fig. 2). While this may represent the natural geoclimatic confines of ticks within the country, it will be important for future studies to confirm the distribution of tick species within these southern states. There are also gaps concerning the surveillance of tick-borne pathogens present within ticks collected from Nigeria. Of note, most studies that included pathogen detection experiments focused on ticks collected from domestic animals. This leaves the characterization of tick-borne pathogen ecology within Nigeria largely incomplete, with insufficient knowledge concerning microbial species present within environmental ticks and ticks collected from wildlife, and how this relates to the relevance of transstadial and transovarial transmission of TBPs within the country.

### Senegal

The results demonstrate that Senegal is among the more characterized countries within this systematic review, with many published studies focusing their efforts on the northwestern and southeastern regions of the country. These studies reported a variety of *Hyalomma* spp. and *Rhipicephalus* spp., as well as the greatest number of unique *Argas* species among the target countries. The reported *Hyalomma* spp. and *Rhipicephalus* spp. were associated with numerous high-consequence pathogens for human health, including *C. burnetii*, CCHFV, and pathogenic *Rickettsia* spp. These pathogens have also been detected within humans and domestic animals within Senegal, demonstrating active transmission within the country.

Livestock play a key role in the daily lives of the Senegalese population, with around 60% practicing pastoralism [394]. Given the extensive time spent in tick-dense habitats, these pastoralists may face an occupationally increased risk for acquisition of tick-borne pathogens. Additionally, the reports of numerous ticks carrying *C. burnetii* and rickettsial pathogens represent a significant threat to pastoralist herd health, with herd outbreaks having the potential to affect income and food security [319, 322, 395]. Given the diversity of ticks and tick-borne pathogens reported, especially in rural areas, further research is needed to continue characterizing tick and tick-borne pathogen ecology within Senegal [326].

### Sierra Leone

The results for Sierra Leone clearly demonstrate a large gap in knowledge concerning tick species diversity and distribution within the country. It is of particular concern that no studies have attempted to characterize the diversity of microbial species present within ticks from Sierra Leone, as these vectors and the diseases they spread may represent a significant source of morbidity and mortality to humans and animals. Many of the tick species that have been reported within Sierra Leone have been proven vectors for a variety of tick-borne pathogens, including *Amblyomma variegatum* (*Ehrlichia ruminantium* and *R. africae*), *Rhipicephalus annulatus* (*Babesia bovis*, *B. bigemina*, and *Anaplasma marginale*), and *Rhipicephalus sanguineus* s.l. (*C. burnetii*, *E. canis*, and *R. conorii*) [396–398].

Previous studies have demonstrated that humans and animals within Sierra Leone are positive or seropositive for high-consequence tick-borne pathogens such as *Anaplasma* spp., CCHFV, and *Rickettsia* spp. [399–401]. In addition to their direct impact on human health, these pathogens can have significant impacts on animal health and productivity. Within Sierra Leone, cattle and small ruminant animals are typically raised by individual families as part of subsistence farming [402, 403]. Given this individual-level dependency on livestock farming, uncontrolled spread of unrecognized endemic TBPs may represent a significant threat to food security and individual income within Sierra Leone.

### Tunisia

Although a few studies reported on the presence of several species of ticks and TBPs, there is still limited information concerning the epidemiology, distribution, and ecology of these species in Tunisia. Several studies identified the presence of *Hyalomma* ticks within Tunisia, which can carry a variety of pathogens that can affect both animals and humans. These include *Theileria* spp., responsible for tropical theileriosis, and *Coxiella burnetii*, both of which have been reported within the country [404, 405]. Of note, while recent studies have demonstrated past exposure of Tunisian ruminants to CCHFV, our review did not find evidence of this virus having been reported in ticks collected within this country [406–408]. Considering the impact that these diseases can have on both the agricultural industry and human health, an increase in surveillance is needed to further characterize the risk posed by tick species and the diseases they vector within Tunisia.

## Limitations

Perhaps the most significant limitation to this study is that it was focused on peer-reviewed articles only. Any data generated by government health workers or nongovernmental organizations (NGOs) that were not published in a peer-reviewed journal are not included in this study. As a result, we do not have a complete picture of ticks and TBP distributions and therefore risk. Another important limitation is that the targeted articles were limited to English and French only. Knowing that there are numerous languages spoken within each target country, the captured articles may not encompass all available tick literature. Additionally, our results highlight that our findings are heavily based on collections from domestic livestock and may not be fully representative of the environment. Another limitation is, given the time span examined, there are records that likely underwent taxonomic reclassification, which is why we have reports of species presence that is contested according to team expertise and Guglielmone et al. (2014) [409]. While modern tick collection events often include a component of molecular confirmation for tick speciation, older collections typically relied solely on morphology, and thus there may be inaccuracies with reported species. Inaccuracies regarding taxonomy may be of particular note for the reported soft ticks, given the general lack of consensus concerning soft tick genera and species taxonomy [410]. Finally, our search was limited to only articles that provide mappable collection event data.

## Conclusions

The collection of this tick and TBP information is essential to help to improve our current understanding of the presence, diversity, and abundance of ticks within these seven North and West African countries.

This systematic review provides insight into knowledge gaps of ticks and TBPs within each of the target countries. For instance, we were able to assess that countries such as Niger (*n* = 6) and Sierra Leone (*n* = 7) have very limited published articles, and therefore, the true tick diversity, abundance, and pathogen ecology is relatively unknown, highlighting an area that needs to be further developed and explored. In relation to the quantity of data collected, this systematic review also helps illustrate the need to improve and standardize the overall quality of tick surveillance information so that these studies can be utilized in future analyses. Ensuring that data are of high quality relies both on the ability for individuals to be properly trained on the best techniques for data collection during surveillance studies, and for further capacity building to ensure that there is a foundation for continual tick and TBP research.

## Supplementary Information

Additional file 1. PRISMA Checklist.docx

Additional file 2. Mastersheet.xlsx

Additional file 3. Article List.docx

Additional file 4. Timeline of Publications Over Decades.pdf

Additional file 5. Comprehensive list of all tick species identified.xlsx

Additional file 6. Tick pathogen profile_Algeria.xslx

Additional file 7. Tick pathogen profile_Morocco.xlsx

Additional file 8. Tick pathogen profile_Niger.xlsx

Additional file 9. Tick pathogen profile_Nigeria.xlsx

Additional file 10. Tick pathogen profile_Senegal.xlsx

Additional file 11. Tick pathogen profile_Tunisia.xlsx

## Data Availability

The dataset supporting the conclusions of this article is included within the article (and its additional file(s)). The dataset including the articles, GPS coordinates, and relevant fields can be found in Additional File 2.

## Abbreviations

TBP: Tick-borne pathogens
ASF: African swine fever
CCHFV: Crimean–Congo hemorrhagic fever virus
GIS: Geographic information system
DUGV: Dugbe virus
JOSV: Jos virus
THOV: Thogoto virus
BHAV: Bhanja virus
NGOV: Ngoye virus
TBEV: Tick-borne encephalitis virus

## References

[1] Organization for Economic Co-operation and Development. The structure of livestock trade in West Africa. 2020. https://www.oecd.org/countries/mali/the-structure-of-livestock-trade-in-west-africa-f8c71341-en.htm Accessed 3 May 2024.

[2] Food and Agriculture Organization. Livestock Contribution to Food Security in the Near East and North Africa. 2016. https://www.fao.org/family-farming/detail/en/c/897879/ Accessed 3 May 2024.

[3] Adakal H, Biguezoton A, Zoungrana S, Courtin F, De Clercq EM, Madder M. Alarming spread of the Asian cattle tick *Rhipicephalus microplus* in West Africa—another three countries are affected: Burkina Faso, Mali and Togo. Exp Appl Acarol. 2013;61:383–6. 23722233 10.1007/s10493-013-9706-6

[4] Addo SO, Bentil RE, Baako BOA, Addae CA, Larbi JA, Baidoo PK, et al. First record of *Rhipicephalus* (*Boophilus*) *microplus* in Ghana, a potential risk to livestock production. Exp Appl Acarol. 2023;89:475–83. 37052725 10.1007/s10493-023-00793-4

[5] Perez-Martinez MB, Moo-Llanes DA, Ibarra-Cerdeña CN, Romero-Salas D, Cruz-Romero A, López-Hernández KM, et al. Worldwide comparison between the potential distribution of *Rhipicephalus microplus* (Acari: Ixodidae) under climate change scenarios. Med Vet Entomol. 2023;37:745–53. 37427707 10.1111/mve.12680

[6] Diarra AZ, Kelly P, Davoust B, Parola P. Tick-borne diseases of humans and animals in West Africa. Pathogens. 2024;202:1276. 10.3390/pathogens12111276 PMC10675719 38003741

[7] Perveen N, Muzaffar SB, Al-Deeb MA. Ticks and tick-borne diseases of livestock in the Middle East and North Africa: a review. Insects. 2021;12:83. 33477991 10.3390/insects12010083 PMC7835866

[8] Lilak AA, Pecor DB, Matulis G, Potter AM, Wofford RN, Kearney MF, et al. Data release: targeted systematic literature search for tick and tick-borne pathogen distributions in six countries in sub-Saharan Africa from 1901 to 2020. Parasit Vectors. 2024;17:84. 38389097 10.1186/s13071-023-06086-4 PMC10885379

[9] Djiman TA, Biguezoton AS, Saegerman C. Tick-borne diseases in sub-Saharan Africa: a systematic review of pathogens, research focus, and implications for public health. Pathogens. 2024;13:697. 39204297 10.3390/pathogens13080697 PMC11356977

[10] Wieczorek JQG, Hijmans R. The point-radius method for georeferencing locality descriptions and calculating associated uncertainty. Int J Geogr Inf Sci. 2004;18:745–67.

[11] Aouragh H, Chaibi R, Bachir AS. Infestation modalities of *Hyalomma aegyptium* (Acari, Oxydidae) on the spur-thighed tortoise *Testudo graeca* in semiarid areas of Algeria. Vie Milieu. 2020;70:99–105.

[12] Belabed AI, Zediri H, Shehab A, Bouslama Z. The effect of altitude on seasonal dynamics of ticks (Acari: Ixodida) in northeastern Algeria. Adv Environ Biol. 2015;9:169–84.

[13] Benchikh-Elfegoun MC, Benakhla A, Bentounsi B, Bouattour A, Piarroux R. Identification et cinétique saisonnière des tiques parasites des bovins dans la région de Taher (Jijel) Algérie. Ann Med Vet. 2007;151:209–14.

[14] Bendjeddou ML, Bouslama Z, Amr ZS, BaniHani R. Infestation and seasonal activity of *Ixodes vespertilionis* Koch, 1844 (Acari: Ixodidae) on the Maghreb mouse-eared bat, *Myotis punicus* Felten, 1977, in Northeastern Algeria. J Vector Ecol. 2016;41:110–3. 27232132 10.1111/jvec.12201

[15] Benredjem W, Leulmi H, Bitam I, Raoult D, Parola P. *Borrelia garinii* and *Rickettsia monacensis* in *Ixodes ricinus* ticks, Algeria. Emerg Infect Dis. 2014;20:1776–7. 25272139 10.3201/eid2010.140265 PMC4193170

[16] Benyahia H, Diarra AZ, Gherissi DE, Bérenger JM, Benakhla A, Parola P. Molecular and MALDI-TOF MS characterization of *Hyalomma aegyptium* ticks collected from turtles and their associated microorganisms in Algeria. Ticks Tick Borne Dis. 2022;13:101858. 34814065 10.1016/j.ttbdis.2021.101858

[17] Bitam I, Kernif T, Harrat Z, Parola P, Raoult D. First detection of *Rickettsia aeschlimannii* in *Hyalomma aegyptium* from Algeria. Clin Microbiol Infect. 2009;15:253–4. 19548989 10.1111/j.1469-0691.2008.02274.x

[18] Bitam I, Parola P, Matsumoto K, Rolain JM, Baziz B, Boubidi SC, et al. First molecular detection of *R. conorii*, *R. aeschlimannii*, and *R. massiliae* in ticks from Algeria. Ann N Y Acad Sci. 2006;1078:368–72. 17114743 10.1196/annals.1374.073

[19] Bouchama B, Dik B, Benia F, Mouffok C. Dynamique saisonnière des tiques (Acari: Ixodidae) parasites des bovins dans la région semi-aride de la wilaya de Sétif Algérie. Bull Soc Zool Fr. 2020;145:71–81.

[20] Bouhous A, Aissi M, Harhoura K. Prevalence of Ixodidae in sheep brought for slaughter in Adrar municipal abattoir, Southwest Algeria. Sci Parasitol. 2011;12:197–201.

[21] Boularias G, Azzag N, Galon C, Šimo L, Boulouis HJ, Moutailler S. High-throughput microfluidic real-time PCR for the detection of multiple microorganisms in Ixodid cattle ticks in Northeast Algeria. Pathogens. 2021;10:362. 33803682 10.3390/pathogens10030362 PMC8002991

[22] Boularias G, Azzag N, Gandoin C, Bouillin C, Chomel B, Haddad N, et al. *Bartonella bovis* and *Bartonella chomelii* infection in dairy cattle and their ectoparasites in Algeria. Comp Immunol Microbiol Infect Dis. 2020;70:101450. 32126432 10.1016/j.cimid.2020.101450

[23] Boulkaboul A. Parasitisme des tiques (Ixodidae) des bovins à Tiaret, Algérie. Rev Elev Med Vet Pays Trop. 2003;56:157–62.

[24] Bouslama Z, Soualah-Alila H, Belabed A, Ouali K. Etude du système Tiques-lézard dans le parc national d’El Kala (Nord-Est algérie). Mésogée. 2009;65:73–83.

[25] Chastel C, Bailly-Choumara H, Bach-Hamba D, Le Lay G, Legrand MC, Le Goff F, et al. Arbovirus transmis par des tiques au Maghreb [tick-transmitted arbovirus in Maghreb]. Bull Soc Pathol Exot. 1995;88:81–5. 8555772

[26] Dib L, Bitam I, Bensouilah M, Parola P, Raoult D. First description of *Rickettsia monacensis* in *Ixodes ricinus* in Algeria. Clin Microbiol Infect. 2009;15:261–2. 19486071 10.1111/j.1469-0691.2008.02277.x

[27] Dib L, Lafri I, Boucheikhchoukh M, Dendani Z, Bitam I, Benakhla A. Seasonal distribution of *Rickettsia* spp. in ticks in Northeast Algeria. New Microbes New Infect. 2018;27:48–52. 30622709 10.1016/j.nmni.2018.10.008 PMC6304374

[28] Djerbouh A, Kernif T, Beneldjouzi A, Socolovschi C, Kechemir N, Parola P, et al. The first molecular detection of *Rickettsia aeschlimannii* in the ticks of camels from southern Algeria. Ticks Tick Borne Dis. 2012;3:374–6. 23168055 10.1016/j.ttbdis.2012.10.014

[29] Djerbouh A, Lafri I, Kechemir-Issad N, Bitam I. Endo-and ectoparasites (Ixodidae) of camels (*Camelus dromedarius*) from Southern Algeria. Livestock Res Rural Dev. 2018;30:1.

[30] Foley H, Parrot L. On the occurrence of *Ornithodorus marocanus* Velu in Algeria. Bull Soc Pathol Exot. 1929;22:436.

[31] Hornok S, Sándor AD, Tomanović S, Beck R, D’Amico G, Kontschán J, et al. East and west separation of *Rhipicephalus sanguineus* mitochondrial lineages in the Mediterranean Basin. Parasit Vectors. 2017;10:39. 28115024 10.1186/s13071-017-1985-z PMC5260041

[32] Kaaboub EA, Ouchene N, Ouchene NA, Dahmani A, Ouchtati I, Haif A, et al. Investigation of the principal vectors of abortive diseases in one-humped camels (*Camelus dromedarius*). Iraqi J Vet Sci. 2021;35:411–5.

[33] Kautman M, Tiar G, Papa A, Široký P. AP92-like Crimean-Congo hemorrhagic fever virus in *Hyalomma aegyptium* ticks, Algeria. Emerg Infect Dis. 2016;22:354–6. 26812469 10.3201/eid2202.151528 PMC4734512

[34] Kebbi R, Nait-Mouloud M, Hassissen L, Ayad A. Seasonal activity of ticks infesting domestic dogs in Bejaia province, Northern Algeria. Onderstepoort J Vet Res. 2019;86:e1–6. 31714138 10.4102/ojvr.v86i1.1755 PMC6852545

[35] Kernif T, Djerbouh A, Mediannikov O, Ayach B, Rolain JM, Raoult D, et al. *Rickettsia africae* in *Hyalomma dromedarii* ticks from sub-Saharan Algeria. Ticks Tick Borne Dis. 2012;3:377–9. 23164496 10.1016/j.ttbdis.2012.10.013

[36] Kernif T, Messaoudene D, Ouahioune S, Parola P, Raoult D, Bitam I. Spotted fever group rickettsiae identified in *Dermacentor marginatus* and *Ixodes ricinus* ticks in Algeria. Ticks Tick Borne Dis. 2012;3:380–1. 23168054 10.1016/j.ttbdis.2012.10.012

[37] Khaldi M, Socolovschi C, Benyettou M, Barech G, Biche M, Kernif T, et al. Rickettsiae in arthropods collected from the North African hedgehog (*Atelerix algirus*) and the desert hedgehog (*Paraechinus aethiopicus*) in Algeria. Comp Immunol Microbiol Infect Dis. 2012;35:117–22. 22222114 10.1016/j.cimid.2011.11.007

[38] Kheira LA, Radhwane SA, Nora MI, Farouk BE, Ratiba BA, Rachid CH, et al. The study of ectoparasites and mesoparasites in turtles (*Testudo Graeca Graeca*) in the Region of Laghouat (South of Algeria). Bull Univ Agric Sci Vet Med Cluj-Napoca. 2020;77:61–9.

[39] Khelifi-Ouchene NA, Ouchene N, Dahmani A, Kaaboub EA, Ouchetati I, Haif A. Investigation of internal and external parasites of the camels (*Camelus dromedarius*) in Algeria. Ann Parasitol. 2020;66:331–7. 33128515 10.17420/ap6603.271

[40] Kiouani A, Azzag N, Tennah S, Ghalmi F. Infection with *Babesia canis* in dogs in the Algiers region: parasitological and serological study. Vet World. 2020;13:1351–7. 32848310 10.14202/vetworld.2020.1351-1357 PMC7429378

[41] Laatamna A, Oswald B, Chitimia-Dobler L, Bakkes DK. Mitochondrial 16S rRNA gene analysis reveals occurrence of *Rhipicephalus sanguineus* sensu stricto from steppe and high plateaus regions, Algeria. Parasitol Res. 2020;119:2085–91. 32458117 10.1007/s00436-020-06725-0

[42] Lafri I, Benredjem W, Neffah-Baaziz F, Lalout R, Abdelouahed K, Gassen B, et al. Inventory and update on argasid ticks and associated pathogens in Algeria. New Microbes New Infect. 2018;23:110–4. 29692914 10.1016/j.nmni.2018.02.009 PMC5913355

[43] Lafri I, El Hamzaoui B, Bitam I, Leulmi H, Lalout R, Mediannikov O, et al. Detection of relapsing fever *Borrelia* spp., *Bartonella* spp. and *Anaplasmataceae* bacteria in argasid ticks in Algeria. PLoS Negl Trop Dis. 2017;11:e0006064. 29145396 10.1371/journal.pntd.0006064 PMC5708834

[44] Lakehal K, Saidi R, Rahmani MM, Kaidi R, Mimoune N, Benaceur F. Razlika u infestaciji krpeljima: *Hyalomma dromedarii Rhipicephalus sanguineus* sensu lato na jugu Alžira. Vet Stanica. 2021;16:331–7.

[45] Lo N, Beninati T, Sassera D, Bouman EA, Santagati S, Gern L, et al. Widespread distribution and high prevalence of an alpha-proteobacterial symbiont in the tick *Ixodes ricinus*. Environ Microbiol. 2006;8:1280–7. 16817936 10.1111/j.1462-2920.2006.01024.x

[46] Lotfi D, Karima K. Identification and incidence of hard tick species during summer season 2019 in Jijel Province (Northeastern Algeria). J Parasit Dis. 2021;45:211–7. 33746406 10.1007/s12639-020-01296-4 PMC7921241

[47] Matallah F, Benakhla A, Medjouel L, Matallah S. Tick infestation of dogs and prevalence of canine babesiosis in the North-East of Algeria; area of El-Tarf. Am-Eurasian J Sustain. 2012;1:126–35.

[48] Mokhtaria K, Ammar AA, Mohammed Ammar SS, Chahrazed K, Fadela S, Belkacem BT. Survey on species composition of Ixodidae tick infesting cattle in Tiaret (Algeria). Trop Agric. 2018;95:102–5.

[49] Ouchene N, Nebbak A, Ouchene-Khelifi NA, Dahmani A, Zeroual F, Khelef D, et al. Molecular detection of avian spirochete *Borrelia anserina* in *Argas persicus* ticks in Algeria. Comp Immunol Microbiol Infect Dis. 2020;68:101408. 31896047 10.1016/j.cimid.2019.101408

[50] Rahal M, Medkour H, Diarra AZ, Bitam I, Parola P, Mediannikov O. Molecular identification and evaluation of *Coxiella*-like endosymbionts genetic diversity carried by cattle ticks in Algeria. Ticks Tick-borne Dis. 2020;11:101493. 32723650 10.1016/j.ttbdis.2020.101493

[51] Randa ML, Meddour S, Bilal DI, Souttou K, Sekour M. First report of ectoparasites from black rats (*Rattus rattus* Linnaeus, 1758) in oasis regions from Algeria. Not Sci Biol. 2022;14:11013.

[52] Sadeddine R, Diarra AZ, Laroche M, Mediannikov O, Righi S, Benakhla A, et al. Molecular identification of protozoal and bacterial organisms in domestic animals and their infesting ticks from North-eastern Algeria. Ticks Tick-borne Dis. 2020;11:101330. 31786146 10.1016/j.ttbdis.2019.101330

[53] Sakraoui F, Boukheroufa M, Sakraoui W, El Madoui MB. Ectoparasitic ecology of Algerian hedgehog *Ateleris algirus* (Lereboullet, 1842)(Erinaceidae, Mammalia) in some localities of Edough Montain (W. Annaba, Northeast Algeria). Adv Environ Biol. 2014;1:217–22.

[54] Senevet G, Pampiglione S. Some species of Ixodidae from the Geryville region (high plateaus of Oran). Bull Soc Pathol Exotique. 1964;57:400–2. 14254065

[55] Socolovschi C, Bitam I, Raoult D, Parola P. Transmission of *Rickettsia conorii conorii* in naturally infected *Rhipicephalus sanguineus*. Clin Microbiol Infect. 2009;15:319–21. 19438619 10.1111/j.1469-0691.2008.02257.x

[56] Soualah-Alila H, Bouslama Z, Amr Z, Bani Hani R. Tick infestations (Acari: Ixodidae) on three lizard species from Seraidi (Annaba district), northeastern Algeria. Exp Appl Acarol. 2015;67:159–63. 26071102 10.1007/s10493-015-9932-1

[57] Tiar G, Tiar-Saadi M, Benyacoub S, Rouag R, Široký P. The dependence of *Hyalomma aegyptium* on its tortoise host *Testudo graeca* in Algeria. Med Vet Entomol. 2016;30:351–9. 27218892 10.1111/mve.12175

[58] Yousfi-Monod R. Annual evolution of the sex ratio of *Rhipicephalus sanguineus* (Acarina, Ixodidae) in an urban area of western Algeria. Acarologia. 1985;26:361–5.

[59] Yousfi-Monod R, Aeschlimann A, Derscheid JM. 3 infections by trypanosomes observed in *Hyalomma detritum*, *Ixodes ricinus* and *Rhipicephalus sanguineus* (Acarina: Ixodidae). Schweiz Arch Tierheilkd. 1986;128:243–54. 3726517

[60] Zeroual F, Bitam I, Ouchene N, Leulmi H, Aouadi A, Benakhla A. Identification and seasonal dynamics of ticks on wild boar (*Sus scrofa*) in the extreme North-east of Algeria. Bull Soc Zool Fr. 2014;139:245–53.

[61] Ziam H, Saidani K, Aissi M. Prevalence of bovine piroplasmosis and anaplasmosis in North-central Algeria. Sci Parasitol. 2017;18:7–15.

[62] Harris DJ, Graciá E, Jorge F, Maia JP, Perera A, Carretero MA, et al. Molecular detection of *Hemolivia* (Apicomplexa: Haemogregarinidae) from ticks of North African *Testudo graeca* (Testudines: Testudinidae) and an estimation of their phylogenetic relationships using 18S rRNA sequences. Comp Parasitol. 2013;80:292–6.

[63] Široký P, Mikulíček P, Jandzík D, Kami H, Mihalca AD, Rouag R, et al. Co-distribution pattern of a haemogregarine *Hemolivia mauritanica* (Apicomplexa: Haemogregarinidae) and its vector *Hyalomma aegyptium* (Metastigmata: Ixodidae). J Parasitol. 2009;95:728–33. 18954156 10.1645/GE-1842.1

[64] Duron O, Binetruy F, Noël V, Cremaschi J, McCoy KD, Arnathau C, et al. Evolutionary changes in symbiont community structure in ticks. Mol Ecol. 2017;26:2905–21. 28281305 10.1111/mec.14094

[65] Norte AC, Harris DJ, Silveira D, Nunes CS, Núncio MS, Martínez EG, et al. Diversity of microorganisms in *Hyalomma aegyptium* collected from spur-thighed tortoise (*Testudo graeca*) in North Africa and Anatolia. Transbound Emerg Dis. 2022;69:1951–62. 34125999 10.1111/tbed.14188

[66] Estrada-Peña A, Nava S, Petney T. Description of all the stages of *Ixodes inopinatus* n. sp. (Acari: Ixodidae). Ticks Tick-borne Dis. 2014;5:734–43. 25108790 10.1016/j.ttbdis.2014.05.003

[67] Ait Lbacha H, Zouagui Z, Alali S, Rhalem A, Petit E, Ducrotoy MJ, et al. “*Candidatus* anaplasma camelii” in one-humped camels (*Camelus dromedarius*) in Morocco: a novel and emerging *Anaplasma* species? Infect Dis Poverty. 2017;6:1–8. 28160773 10.1186/s40249-016-0216-8 PMC5292149

[68] Beati L, Meskini M, Thiers B, Raoult D. *Rickettsia aeschlimannii* sp. nov., a new spotted fever group rickettsia associated with *Hyalomma marginatum* ticks. Int J Syst Bacteriol. 1997;47:548–54. 9103647 10.1099/00207713-47-2-548

[69] Black F, Eley SM, Nuttall PA, Moore NF. Characterisation of orbiviruses of the Kemerovo serogroup: comparison of protein and RNA profiles. Acta Virol. 1986;30:320–4. 2876612

[70] Boudebouch N, Sarih M, Socolovschi C, Amarouch H, Hassar M, Raoult D, et al. Molecular survey for spotted fever group rickettsiae in ticks from Morocco. Clin Microbiol Infect. 2009;15:259–60. 19456813 10.1111/j.1469-0691.2008.02226.x

[71] Buysse M, Duron O. Two novel *Rickettsia* species of soft ticks in North Africa:‘*Candidatus* Rickettsia africaseptentrionalis’ and ‘*Candidatus* Rickettsia mauretanica’. Ticks Tick-borne Dis. 2020;11:101376. 32005627 10.1016/j.ttbdis.2020.101376

[72] Chastel C, Lay L. Pouvoir pathogène naturel pour l'homme d'un variant antigénique du virus Soldado isolé au Maroc. 1981;74; 499-505 6274527

[73] Chastel C, Main AJ, Bailly-Choumara H, Le Goff F, Le Lay G. Essaouira and Kala iris: two new orbiviruses of the Kemerovo serogroup, Chenuda complex, isolated from *Ornithodoros* (*Alectorobius*) *maritimus* ticks in Morocco. Acta Virol. 1993;37:484–92. 8010186

[74] Elbir H, FotsoFotso A, Diatta G, Trape JF, Arnathau C, Renaud F, et al. Ubiquitous bacteria *Borrelia crocidurae* in Western African ticks *Ornithodoros sonrai*. Parasites Vectors. 2015;8:1–5. 26382232 10.1186/s13071-015-1089-6 PMC4574609

[75] El Mouden EH, Laghzaoui EM, Elbahi A, Abbad A. A case of massive infestation of a female spur-thighed tortoise *Testudo graeca* by blood-sucking ticks *Hyalomma aegyptium* (Acari: Ixodidae). Int J Acarol. 2020;46:63–5.

[76] Elhachimi L, Rogiers C, Casaert S, Fellahi S, Van Leeuwen T, Dermauw W, et al. Ticks and tick-borne pathogens abound in the cattle population of the Rabat-Sale Kenitra region, Morocco. Pathogens. 2021;10:1594. 34959550 10.3390/pathogens10121594 PMC8703448

[77] Elhamiani Khatat S, Daminet S, Kachani M, Leutenegger CM, Duchateau L, El Amri H, et al. Anaplasma spp in dogs and owners in North-western Morocco. Parasit Vect. 2017;10:1. 10.1186/s13071-017-2148-y PMC5404288 28438220

[78] Er-Rguibi O, Laghzaoui EM, Aglagane A, Kimdil L, Abbad A, El Mouden EH. Determinants of prevalence and co-infestation by ecto-and endoparasites in the Atlas day gecko, *Quedenfeldtia trachyblepharus*, an endemic species of Morocco. Parasitol Res. 2021;120:2543–56. 33748890 10.1007/s00436-021-07120-z

[79] Flach EJ, Ouhelli H, Waddington D, Hasnaoui ME. Prevalence of *Theileria* in the tick *Hyalomma detritum detritum* in the Doukkala region, Morocco. Med Vet Entomol. 1993;7:343–50. 8268489 10.1111/j.1365-2915.1993.tb00703.x

[80] Gemel R, Hörweg C. Zum Befall der Maurischen Landschildkröte *Testudo graeca* Linnaeus, 1758 durch Zecken, und deren Bedeutung als Vektoren. Ein Literaturüberblick samt eigenen Beobachtungen (Testudines: Testudinidae). Herpetozoa. 2011;23:21–30.

[81] Khallaayoune K, Biron JM, Chaoui A, Duvallet G. Efficacy of 1% geraniol (Fulltec®) as a tick repellent. Parasite. 2009;16:223–6. 19839268 10.1051/parasite/2009163223

[82] Laamri M, El Kharrim K, Mrifag R, Boukbal M, Belghyti D. Population dynamics of cattle parasitic ticks in the Gharb region of Morocco. J Anim Husb Vet Med Trop Countries. 2012;2012:65:57.

[83] Laghzaoui EM, Kasrati A, Abbad A, Leach D, Spooner-Hart R, El Mouden EH. Acaricidal properties of essential oils from Moroccan plants against immature ticks of *Hyalomma aegyptium* (Linnaeus, 1758); an external parasite of the spur-thighed tortoise (*Testudo graeca*). Int J Acarol. 2018;44:315–21.

[84] Laghzaoui EM, Bouazza A, Abbad A, El Mouden EH. Cross-sectional study of ticks in the vulnerable free-living spur-thighed tortoise *Testudo graeca* (Testudines: Testudinidae) from Morocco. Int J Acarol. 2022;48:76–83.

[85] Ouhelli H, Pandey VS. Prevalence of cattle ticks in Morocco. Trop Anim Health Prod. 1982;14:151–4. 7123663 10.1007/BF02242145

[86] Palomar AM, Portillo A, Santibáñez P, Mazuelas D, Arizaga J, Crespo A, et al. Crimean-Congo hemorrhagic fever virus in ticks from migratory birds. Morocco Emerg Infect Dis. 2013;19:260. 23347801 10.3201/eid1902.121193 PMC3559059

[87] Pandey VS, Dakkak A, Elmamoune M. Parasites of stray dogs in the Rabat region, Morocco. Ann Trop Med Parasitol. 1987;81:53–5. 3675044 10.1080/00034983.1987.11812090

[88] Sahibi H, Rhalem A, Berrag B, Goff WL. Bovine babesiosis: seroprevalence and ticks associated with cattle from two different regions of Morocco. Ann N Y Acad Sci. 1998;849:213–8. 9668467 10.1111/j.1749-6632.1998.tb11051.x

[89] Sarih MH, M’Ghirbi Y, Bouattour A, Gern L, Baranton G, Postic D. Detection and identification of *Ehrlichia* spp. in ticks collected in Tunisia and Morocco. J Clin Microbiol. 2005;43:1127–32. 15750072 10.1128/JCM.43.3.1127-1132.2005 PMC1081246

[90] Sarih M, Socolovschi C, Boudebouch N, Hassar M, Raoult D, Parola P. Spotted fever group rickettsiae in ticks, Morocco. Emerg Infect Dis. 2008;14:1067. 18598627 10.3201/eid1407.070096 PMC2600325

[91] Schein E, Rehbein G, Voigt WP, Zweygarth E. *Babesia equi* (Laveran 1901). Development in horses and in lymphocyte culture. Trop Med Parasitol. 1981;32:223–7. 7345686

[92] Segura A, Rodríguez O, Ruiz-Fons F, Acevedo P. Tick parasitism in the Mediterranean spur-thighed tortoise in the Maamora forest, Morocco. Ticks Tick-borne Dis. 2019;10:286–9. 30459084 10.1016/j.ttbdis.2018.11.002

[93] Seng P, Sarih M, Socolovschi C, Boudebouch N, Hassar M, Parola P, et al. Detection of Anaplasmataceae in ticks collected in Morocco. Clin Microbiol Infect. 2009;15:86–7. 19438648 10.1111/j.1469-0691.2008.02251.x

[94] Abiola FA, Karimou M, Houeto P. Cholinesterase activity in bovine ticks and its inhibition *in vitro* by organophosphate acaricides. Rev Vet Med. 1991;142:147–52.

[95] Hoffmann G, Lindau M. Zecken an Nutz-und Wildtieren in Niger. Zeitschrift für Angewandte Entomol. 1971;69:72–82.

[96] Lempereur L, Geysen D, Madder M. Development and validation of a PCR–RFLP test to identify African *Rhipicephalus* (*Boophilus)* ticks. Acta Trop. 2010;114:55–8. 20080073 10.1016/j.actatropica.2010.01.004

[97] Parola P, Raoult D. Molecular tools in the epidemiology of tick-borne bacterial diseases. Ann Clin Biol. 2001;59:77–82. 11282521

[98] Parola P, Inokuma H, Camicas JL, Brouqui P, Raoult D. Detection and identification of spotted fever group Rickettsiae and Ehrlichiae in African ticks. Emerg Infect Dis. 2001;7:1014–7. 11747731 10.3201/eid0706.010616 PMC2631901

[99] Tager-Kagan P, Tibayrenc R, Garba D. Epidemiology of poultry parasitic disease in village breeding in Niamey area, Niger. Rev Elev Med Vet Pays Trop. 1992;45:139–47. 1301626

[100] Amuta EU, Houmsou RS, Ogabiela M. Tick infestation of dogs in Makurdi metropolis. Benue State-Nigeria J Vet Med. 2010;7:2.

[101] Abdulkareem BO, Christy AL, Samuel UU. Prevalence of ectoparasite infestations in owned dogs in Kwara State, Nigeria. Parasite Epidemiol Control. 2019;4:e00079. 30662964 10.1016/j.parepi.2018.e00079 PMC6324013

[102] Abdullahi YM, Magami IM, Audu A, Mainasara MM. Prevalence of ticks on camels and cattle brought to Dodoru market Kebbi State, Nigeria. Pos. 2018;4:3001–4.

[103] Adamu M, Troskie M, Oshadu DO, Malatji DP, Penzhorn BL, Matjila PT. Occurrence of tick-transmitted pathogens in dogs in Jos, Plateau State, Nigeria. Parasit Vectors. 2014;7:1–8. 24661795 10.1186/1756-3305-7-119 PMC3974742

[104] Adamu NB, Adamu JY, Salisu L. Prevalence of ecto-, endo-and haemoparasites in slaughtered dogs in Maiduguri, Nigeria. Rev Vet Med. 2012;163:178–82.

[105] Adang KL, Ayuba J, Yoriyo KP. Ectoparasites of sheep (*Ovis aries* L.) and goats (*Capra hirus* L.) in Gombe, Gombe State, Nigeria. Pak J Biol Sci. 2015;18:224–31.

[106] Adang LK, Oniye SJ, Ezealor AU, Abdu PA, Ajanusi JO. Ectoparasites of the laughing dove *Streptopelia senegalensis* (Linnaeus, 1766)(Aves: Columbidae) in Zaria, Nigeria. Lundiana Biodivers Int J. 2008;9:67–71.

[107] Adejoh VA, Pam VA, Uzoigwe NR, Naphtali RS, Yohanna JA, Pam RG, et al. Prevalence of hard ticks infesting cattle in Lafia, Nasarawa State, North Central Nigeria. J Agric Res Nat Resour. 2019;3:1–19.

[108] Adelusi SM, Vajime CG, Omudu EA, Okpotu RO, Onazi FO. Avian ectoparasitism in Makurdi, Nigeria: do wild birds serve as reservoir for domestic birds? Nigerian Ann Pure Appl Sci. 2015;6:11–5.

[109] Ademola IO, Akinboade OA. Increase exposure of *Rhipicephalus* (*Boophilus) decoloratus* (Koch, 1844)(Acarina: Ixodidae) to ultraviolet radiation affects its reproductive capacity. Int J Acarol. 2016;42:412–5.

[110] Adenubi OT, Abolaji AO, Salihu T, Akande FA, Lawal H. Chemical composition and acaricidal activity of *Eucalyptus globulus* essential oil against the vector of tropical bovine piroplasmosis, *Rhipicephalus* (*Boophilus*) *annulatus*. Exp Appl Acarol. 2021;83:301–12. 33389348 10.1007/s10493-020-00578-z

[111] Adeyefa CA, Dipeolu OO. Ectoparasites of horses in south-western Nigeria. Int J Trop Insect Sci. 1986;7:511–3.

[112] Agbede RI. A survey of ectoparasites and ectoparasitic conditions of animals in Zaria (Nigeria). J Anim Prod Res. 1981;1:179–80.

[113] Agbolade OM, Soetan EO, Awesu A, Ojo JA, Somoye OJ, Raufu ST. Ectoparasites of domestic dogs in some Ijebu communities Southwest Nigeria World. Appl Sci J. 2008;3:916–20.

[114] Agu NG, Okoye IC, Nwosu CG, Onyema I, Iheagwam CN, Anunobi TJ. Prevalence of ectoparasites infestation among companion animals in Nsukka cultural zone. Ann Med Health Sci Res. 2020;10:1050–7.

[115] Agwunobi DO, Kamani J, Zheng H, Guo L, Yu Z, Liu J. Bacterial diversity in *Rhipicephalus sanguineus* (Acari: Ixodidae) from two states in Nigeria. J Entomol Sci. 2021;56:256–71.

[116] Ahmed A, George BD. Incidence of hard ticks (Ixodidae) on horses around Zaria, Nigeria. Niger Vet J. 2002;23:70–4.

[117] Ajuwape AT, Sonibare AO, Adedokun RA, Adedokun OA, Adejinmi JO, Akinboye DG. Infestation of royal python (Python regius) with ticks *Amblyomma hebraeum* in Ibadan Zoo. Nigeria Trop Vet. 2003;21:38–41.

[118] Akande FA, Adebowale AF, Idowu OA, Sofela OO. Prevalence of ticks on indigenous breed of hunting dogs in Ogun State, Nigeria. Sokoto J Vet Sci. 2018;16:66–71.

[119] Akinboade OA, Dipeolu OO. Detection of *Babesia bovis* infections in *Boophilus geigyi* with egg crushings, larval smears, and haemolymph puncture. Vet Q. 1981;3:143–7. 7268748 10.1080/01652176.1981.9693815

[120] AkinboadeDipeolu OO. Bovine babesiosis in Nigeria: detection of *Babesia* organisms in salivary glands of *Boophilus decoloratus* collected on trade cattle. Zentralbl Vet B. 1983;30:153–5. 10.1111/j.1439-0450.1983.tb01826.x 6683052

[121] Alayande MO, Mayaki AM, Lawal MD, Abubakat A, Kassu M, Talabi AO. Pattern of ticks and lice infestation on small ruminants in Sokoto. Sokoto State Nigerian J Anim Sci. 2016;18:183–9.

[122] Alayande MO, Mayaki AM, Lawal MD, Bandi NI, Ibrahim DD, Talabi AO. Pattern of tick infestation on one humped camels (*Camelus dromedarius*) in Sokoto, Nigeria. Bull Anim Health Prod Afr. 2015;63:349–54.

[123] Ameen SA, Odetokun IA, Ghali-Muhammed LI, Azeez OM, Raji LO, Kolapo TU, et al. Status of ticks infestation in ruminant animals in Ogbomoso area of Oyo State, Nigeria. J Environ Issues Agric Dev Ctries. 2014;6:48–53.

[124] Ameh IG. A description of some ectoparasites of the wall gecko. J Entomol. 2005;2:21–4.

[125] Amoo AO, Dipeolu OO, Akinboade AO, Adeyemi A. Bacterial isolation from and transmission by *Boophilus decoloratus* and *Boophilus geigyi*. Folia Parasitol. 1987;34:69–74. 3108116

[126] Amuta E, Atu B, Houmsou R, Ayashar J. Prevalence of *Rhipicephalus sanguineus* infestation and *Babesia canis* infection in dogs with respect to breed type and degree of freedom in Makurdi, Benue State, Nigeria. Int J Parasit Dis. 2010;4:247–9.

[127] Amuta EU, Atu BO, Houmsou RS, Ayashar JG. *Rhipicephalus sanguineus* infestation and *Babesia canis* infection among domestic dogs in Makurdi, Benue state- Nigeria. Int J Acad Res. 2010;2:3.

[128] Anifowose OI, Takeet MI, Talabi AO, Otesile EB. Molecular detection of *Ehrlichia ruminantium* in engorged *Amblyomma variegatum* and cattle in Ogun State, Nigeria. J Parasitol. 2020;44:403–10. 10.1007/s12639-020-01218-4 PMC7244682 32508415

[129] Anyaegbunam LC, Obi ZC, Chinasa ME. Ectoparasitosis and endoparasites in local goats *Capra hircus* in Onitsha, Anambra State, Nigeria. Int J Biol Sci. 2013;1:1–3.

[130] Aquino LC, Kamani J, Haruna AM, Paludo GR, Hicks CA, Helps CR, et al. Analysis of risk factors and prevalence of haemoplasma infection in dogs. Vet Parasitol. 2016;221:111–7. 27084481 10.1016/j.vetpar.2016.03.014

[131] Arong GA, Adetunji BA, Mowang DA, Odu AE. Comparative distribution of ticks on dogs in the Calabar Metropolis, South-South Nigeria. Eur J Zool Res. 2013;2:14–8.

[132] Arong GA, Okon OE, Obhiokhenan AA, Esekhagbe OR, Okorafor KA, Emevatha O. The infestation rates and predilection sites of ticks on cattles and dogs in Calabar, Nigeria. Int J Curr Res. 2012;4:73–6.

[133] Arong GA, Shitta KB, James-Rugu NN, Effanga EO. Seasonal variation in the abundance and distribution of Ixodid ticks on Mongrel, Alsatian and mixed breeds of dogs *Canis familiaris* in Jos, in Plateau state, North-central Nigeria. World J Sci Technol. 2011;1:24–9.

[134] Ayeni JS, Dipeolu OO, Okaeme AN. Parasitic infections of the grey-breasted helmet guinea-fowl *Numida meleagris galeata* in Nigeria. Vet Parasitol. 1983;12:59–63. 6683038 10.1016/0304-4017(83)90088-2

[135] Bayer W, Maina JA. Seasonal pattern of tick load in Bunaji cattle in the subhumid zone of Nigeria. Vet Parasitol. 1984;15:301–7. 6541842 10.1016/0304-4017(84)90082-7

[136] Bida SA, Adams EW. Heartwater in a Bunaji calf: a case report. Vet Rec. 1973;92:200–1. 4705869 10.1136/vr.92.8.200

[137] Biu AA, Konto M. Survey of tick species infesting the one humped camel *Camelus dromedarius* in Borno state, Nigeria. J Agr Vet Sci. 2011;4:1–6.

[138] Bunza MD, Yahaya MM, Muhammad AS, Saidu AR. A survey on tick species infesting domestic birds sold at Sokoto central market, Nigeria. Sokoto J Vet Sci. 2008;7:52–4.

[139] Cafiso A, Bazzocchi C, De Marco L, Opara MN, Sassera D, Plantard O. Molecular screening for *Midichloria* in hard and soft ticks reveals variable prevalence levels and bacterial loads in different tick species. Ticks Tick-borne Dis. 2016;7:1186–92. 27521265 10.1016/j.ttbdis.2016.07.017

[140] Causey OR, Kemp GE, Madbouly MH, David-West TS. Congo virus from domestic livestock, African hedgehog, and arthropods in Nigeria. Am J Trop Med Hyg. 1970;19:846–50. 5453910 10.4269/ajtmh.1970.19.846

[141] Chiejina SN. Some parasitic diseases of intensively managed West African Dwarf sheep and goats in Nsukka. Eastern Nigeria Brit Vet J. 1987;143:264–72. 3594196 10.1016/0007-1935(87)90089-3

[142] Choudhury MK. Toxicity of neem seed oil against the larvae of *Boophilus decoloratus*, a one-host tick in cattle. Indian J Pharm Sci. 2009;71:562. 20502579 10.4103/0250-474X.58191 PMC2866352

[143] Cutler SJ, Idris JM, Ahmed AO, Elelu N. *Ornithodoros savignyi,* the tick vector of “*Candidatus* Borrelia kalaharica” in Nigeria. J Clin Microbiol. 2018;56:e00532–18. 29950331 10.1128/JCM.00532-18 PMC6113498

[144] Daodu OB, Eisenbarth A, Schulz A, Hartlaub J, Olopade JO, Oluwayelu DO, et al. Molecular detection of *dugbe orthonairovirus* in cattle and their infesting ticks (*Amblyomma* and* Rhipicephalus* (*Boophilus*)) in Nigeria. PLoS Negl Trop Dis. 2021;15:e0009905. 34788303 10.1371/journal.pntd.0009905 PMC8598060

[145] Davou KP, Dogo GA, Tanko J, Bialla M, Kogi CA. Epidemiology of ectoparasites infestation in Jos North, Plateau State, Nigeria. Saudi J Med Pharm. 2017;3:206–10.

[146] Deme GG, Malann YD, Olanrewaju CA, Lumi EB. Ticks and tick-borne infections in some livestocks slaughtered at Gwagwalada Abattoir, Federal Capital Territory, Abuja, Nigeria. Niger J Parasitol. 2017;38:258–60.

[147] Dipeolu OO. Studies on ticks of veterinary importance in Nigeria 1983. Int J Acarol. 1983;9:55–61.

[148] Dipeolu OO. A survey of the ectoparasitic infestations of dogs in Nigeria. J Small Anim Pract. 1975;16:123–9.

[149] Dipeolu OO. Survey of tick infestation in the trade cattle and sheep and goats in Nigeria. Bull Anim Health Prod Afr. 1975;23:165–72.

[150] Dipeolu OO. The incidence of ticks of *Boophilus* species on cattle, sheep, and goats in Nigeria. Trop Anim Health Prod. 1975;7:35–9.

[151] Dipeolu OO. The occurrence of ticks on a baby African elephant in Nigeria. Afr J Ecol. 1976;14:227.

[152] Dipeolu OO. Tick paralysis in a sheep caused by nymphs of *Amblyomma variegatum*: a preliminary report. Zeitschrift fr Parasitenkunde. 1976;49:293–5. 10.1007/BF00380599 988681

[153] Dipeolu OO. Studies on ticks of veterinary importance in Nigeria. VII. Comparison of some aspects of the biology of *Boophilus decoloratus* and *Boophilus geigyi*. Trop Vet. 1984;2:22–32.

[154] Dipeolu OO. Studies on ticks of veterinary importance in Nigeria. X. Notes on the biology of ticks of dogs *Rhipicephalus sanguineus* and *Haemaphysalis leachi leachi*. Bull Anim Health Prod Afr. 1984;31:1–15.

[155] Dipeolu OO, Adeyefa CAO. Studies on ticks of veterinary importance in Nigeria. VIII. Differences observed in the biology of ticks which fed on different domestic animal hosts. Folia Parasitol. 1984;31:53–61. 6714846

[156] Dipeolu OO, Oduye OO. Survey of blood parasites of horses in Ibadan Western Nigeria. Ann Parasitol. 1976;22:155–9. 1266263

[157] Dipeolu OO, Ogunji FO. The transmission of *Theileria annulata* to a rabbit by the larvae of the tick *Hyalomma rufipes*. Lab Anim. 1977;11:39–40. 839720 10.1258/002367777780959256

[158] Dipeolu OO, Akinboade OA, Ogunji FO. Observations on the epidemiology of house infesting *Rhipicephalus sanguineus* in a household in Lagos, Nigeria. Bull Anim Health Prod Afr. 1982;30:29–30. 7186809

[159] Dipeou OO, Akinboade OA. Scavenging dogs and the spread of tick infestation in Nigeria. Int J Zoonoses. 1982;9:90–6. 7169311

[160] Edosomwan EU, Oke CO, Evbuomwan IO, Imasuen AA. Prevalence of parasites of some wild birds in a tropical rainforest zone of Edo state, Nigeria. Niger J Parasitol. 2018;39:259–60.

[161] Ejima IA, Ayegba AE. Relative abundance of hard tick on reared cattle (Family: Bovidae; *Bos* pp.) in Idah local government area (LGA), Kogi state, Nigeria. The Zoologist. 2011;9:9–16.

[162] Ejima IA, Obayumi M, Olayemi IK, Dangana MC. Tick infestation among cattle in Minna metropolis Niger state. Nigeria. Res J Appl Sci. 2014;9:126–32.

[163] Ekanem MS, Mbagwu HO, Opara KN, Agbata QC. Ticks infestation of domestic dogs (*Canis familiaris lupus*) in Uyo, Akwa Ibom state, Nigeria. World Appl Sci J. 2010;2:191–6.

[164] Ekpenyong GD. The effect of climate on the seasonal activity and abundance of *Amblyomma variegatum* (Fabricius, 1794) (Acarina: Ixodidae) on trade cattle in Ibadan, Nigeria. Acarologia. 1996;37:165–72.

[165] Elelu N, Ola-Fadunsin SD, Bankole AA, Raji MA, Ogo NI, Cutler SJ. Prevalence of tick infestation and molecular characterization of spotted fever *Rickettsia massiliae* in *Rhipicephalus* species parasitizing domestic small ruminants in North-central Nigeria. PLoS ONE. 2022;17:e0263843. 35157723 10.1371/journal.pone.0263843 PMC8843212

[166] Elelu N, Bankole AA, Daphne HP, Rabiu M, Ola-Fadunsin SD, Ambali HM, et al. Molecular characterisation of *Rhipicephalus sanguineus* sensu lato ticks from domestic dogs in Nigeria. Vet Med Sci. 2022;8:454–9. 35166463 10.1002/vms3.655 PMC8959257

[167] El Kammah KM, Hoogstraal H, Camicas JL. Notes on African *Haemaphysalis* ticks: XI. *H.**(Rhipistoma*)* paraleachi* (Ixodoidea: Ixodidae) distribution and hosts of adults. Int J Acarol. 1992;18:205–12.

[168] Elom MO, Nworie A, Ukwa BN, Uhuo CA, Nwele DE, Ezeruigbo CF. Tick infestations and gastrointestinal helminthosis among goats and cattle at abattoirs in Abakaliki metropolis, Ebonyi state, Nigeria. Niger J Parasitol. 2018;39:248–52.

[169] Eyo JE, Ekeh FN, Ivoke N, Atama CI, Onah IE, Ezenwaji NE, et al. Survey of tick infestation of cattle at four selected grazing sites in the tropics. Glob Vet. 2014;12:479–86.

[170] Ezealor AU. Parasites and diseases of Abdim’s stork Ciconia abdimii. Malimbus. 1985;7:120.

[171] Fabiyi JP. Arthropod parasites of domestic fowl and guineafowl on the Jos plateau Northern Nigera. Trop Anim Health Pro. 1980;12:193–4. 10.1007/BF02242655 7434483

[172] Fabiyi JP, Alayande MO, Lawal MD, Mahmuda A, Usman M. Prevalence of ectoparasites attacking helmet guineafowl, *Numidamele agridis*, in Sokoto, North-western Nigeria. Niger J Parasitol. 2016;37:113–6.

[173] Fabiyi JP, Alayande MO, Lawal MD, Mahmuda A, Usman M. Prevalence and significance of ectoparasites other than lice attacking chickens in Sokoto, North-west Nigeria. Niger J Parasitol. 2017;38:125–7.

[174] Findlay GM, Archer GT. The occurrence of tick-borne typhus in West Africa. Trans R Soc Trop Med Hyg. 1948;41:815–8. 18865444 10.1016/s0035-9203(48)80008-8

[175] Heylen D, Day M, Schunack B, Fourie J, Labuschange M, Johnson S, et al. A community approach of pathogens and their arthropod vectors (ticks and fleas) in dogs of African sub-Sahara. Parasit Vectors. 2021;14:1–20. 34784947 10.1186/s13071-021-05014-8 PMC8594167

[176] Hornok S, Kontschán J, Takács N, Chaber AL, Halajian A, Abichu G, et al. Molecular phylogeny of *Amblyomma exornatum* and *Amblyomma transversale*, with reinstatement of the genus *Africaniella* (Acari: Ixodidae) for the latter. Ticks Tick-borne Dis. 2020;11:101494. 32993922 10.1016/j.ttbdis.2020.101494

[177] Idoko IS, Edeh RE, Adamu AM, Machunga-Mambula S, Okubanjo OO, Balogun EO, et al. Molecular and serological detection of piroplasms in horses from Nigeria. Pathogens. 2021;10:508. 33922468 10.3390/pathogens10050508 PMC8146079

[178] Idris HS, Umar H. Prevalence of ectoparasites in goats (*Capra aegagrus hircus*) brought for slaughter in the Gwagwalada area, Abuja, Nigeria. Entomol Res. 2007;37:25–8.

[179] Ikeme MM. *Haemaphysalis hoodi hoodi* (Warburton and Nuttall, 1909) on domestic chickens in Eastern Nigeria. Vet Rec. 1972;90:33. 5014720 10.1136/vr.90.2.33-a

[180] Ikeme MM. The diagnostic characteristics and host distribution of the larvae of some economically important ticks in Nigeria: The larvae of *Amblyomma variegatum* and *Boophilus decoloratus* of cattle. Nig J Ent. 1976;1–2:69–81.

[181] Ikpeze OO, Eneanya CI, Onyido AE. Abundance of ticks in pasture at Nnamdi Azikiwe University, Awka. Adv Biores. 2016;7:124–31.

[182] Ikpeze OO. Distribution of parasitic ticks (Acarina: Ixodidae) on cattle at designated cattle markets in Enugu, Ugwuoba and Amansea, South-eastern Nigeria. Int J Biol Sci. 2010;2:51–5.

[183] Iwuala MO, Okpala I. Studies on the ectoparasitic fauna of Nigerian livestock I: types and distribution patterns on hosts’. Bull Anim Health Prod Afr. 1978;26:339–50. 756758

[184] Iwuala MO, Okpala I. Studies on the ectoparasitic fauna of Nigerian livestock II: seasonal infestation rates. Bull Anim Health Prod Afr. 1978;26:351–9. 756759

[185] Ikpeze OO, Eneanya CI, Chinweoke OJ, Aribodor DN, Anyasodor AE. Species diversity, distribution and predilection sites of ticks (Acarina: Ixodidae) on trade cattle at Enugu and Anambra states, South-eastern Nigeria. Zoologist. 2011;9:1–8.

[186] Okoye JO, Ikeme MM. Acute dermatitis caused by *Amblyomma variegatum* larvae on chickens. Avian Pathol. 1990;19:785–9. 18679988 10.1080/03079459008418730

[187] Ja’afaru AI, Garba HS, Uko OJ, Lawal MD, Habibullah SA, Shehu SA, et al. Severe ectoparasitism and parasitic gastroenteritis in a two month old Sokoto red kid: a case report. Sokoto J Vet Sci. 2008;7:30–2.

[188] James NN, Iwuala MOE. Seasonal abundance of Ixodid ticks from some livestock in Plateau state. Tech Dev. 1992;2:61–7.

[189] James-Rugu NN, Iwuala MO. Ectoparasites of some domestic animals on the Jos plateau In Nigeria. Sci Forum. 2002;5:146–56.

[190] James-Rugu NN, Sale M. A study on tick borne infections of cattle in Yola locality of Adamawa state. Afr J Agr Res. 2011;6:6208–11.

[191] James-Rugu NN, Iwuala MO. Ticks of Nigerian livestock with different for conditions and colour shades. Afr J Nat Sci. 2002;3:102–6.

[192] James-Rugu NN, Jidayi S. A survey on the ectoparasites of some livestock from some areas of Borno and Yobe states. Nig Vet J. 2004;25:48–55.

[193] Johnston JE. A summary of an entomological survey of Kaduna district Northern Nigeria. B Entomol Res. 1916;7:19–28.

[194] Kamani J, Baneth G, Mumcuoglu KY, Waziri NE, Eyal O, Guthmann Y, et al. Molecular detection and characterization of tick-borne pathogens in dogs and ticks from Nigeria. PLoS Negl Trop Dis. 2013;7:e2108. 23505591 10.1371/journal.pntd.0002108 PMC3591325

[195] Kamani J, Morick D, Mumcuoglu KY, Harrus S. Prevalence and diversity of *Bartonella* species in commensal rodents and ectoparasites from Nigeria, West Africa. PLoS Negl Trop Dis. 2013;7:e2246. 23738028 10.1371/journal.pntd.0002246 PMC3667778

[196] Kamani J. Molecular evidence indicts *Haemaphysalis leachi* (Acari: Ixodidae) as the vector of *Babesia rossi* in dogs in Nigeria, West Africa. Ticks Tick-borne Dis. 2021;12:101717. 33774482 10.1016/j.ttbdis.2021.101717

[197] Kamani J. Molecular identification and genetic analysis of *Rhipicephalus sanguineus* sensu lato of dogs in Nigeria, West Africa. Exp Appl Acarol. 2021;85:277–89. 34686926 10.1007/s10493-021-00664-w

[198] Kamani J, Apanaskevich DA, Gutiérrez R, Nachum-Biala Y, Baneth G, Harrus S. Morphological and molecular identification of *Rhipicephalus* (*Boophilus*) *microplus* in Nigeria, West Africa: a threat to livestock health. Exp Appl Acarol. 2017;73:283–96. 28887701 10.1007/s10493-017-0177-z

[199] Kamani J, Baneth G, Apanaskevich DA, Mumcuoglu KY, Harrus S. Molecular detection of *Rickettsia aeschlimannii* in *Hyalomma* spp. ticks from camels (*Camelus dromedarius*) in Nigeria West Africa. Med Vet Entomol. 2015;2:205–9. 10.1111/mve.12094 25565180

[200] Kamani J, Baneth G, Gutiérrez R, Nachum-Biala Y, Mumcuoglu KY, Harrus S. *Coxiella burnetii* and *Rickettsia conorii*: two zoonotic pathogens in peridomestic rodents and their ectoparasites in Nigeria. Ticks Tick-borne Dis. 2018;9:86–92. 29042240 10.1016/j.ttbdis.2017.10.004

[201] Kamani J, Chung PJ, Lee CC, Chung YT. In search of the vector (s) of *Babesia rossi* in Nigeria: Molecular detection of *B. rossi* DNA in *Rhipicephalus sanguineus sensu lato* (Acari: Ixodidae) ticks collected from dogs, circumstantial evidence worth exploring. Exp Appl Acarol. 2018;76:243–8. 30298231 10.1007/s10493-018-0311-6

[202] Kamani J, González-Miguel J, Mshelbwala FM, Shekaro A, Apanaskevich DA. Ticks (Acari: Ixodidae) infesting dogs in Nigeria: epidemiological and public health implications. Exp Appl Acarol. 2019;78:231–46. 31152319 10.1007/s10493-019-00384-2

[203] Kamani J, Harrus S, Nachum-Biala Y, Gutiérrez R, Mumcuoglu KY, Baneth G. Prevalence of *Hepatozoon* and *Sarcocystis* spp. in rodents and their ectoparasites in Nigeria. Acta Trop. 2018;187:124–8. 30071191 10.1016/j.actatropica.2018.07.028

[204] Kamani J, Jwander LD, Ubali Z. Demonstration of vermicules of *Babesia* species in haemolymph smears of *Amblyomma variegatum* in Nigeria. J Adv Vet Res. 2011;1:1–3.

[205] Kamani J, Sannusi A, Dogo AG, Tanko JT, Egwu KO, Tafarki AE, et al. *Babesia canis* and *Babesia rossi* co-infection in an untraveled Nigerian dog. Vet Parasitol. 2010;173:334–5. 20705395 10.1016/j.vetpar.2010.06.040

[206] Kemp GE, Lee VH, Moore DL. isolation of nyamanini and quaranfil viruses from *Argas *(Persicargas)* arboreus* ticks in Niger. J Med Entomol. 1975;12:535–7. 1223303 10.1093/jmedent/12.5.535

[207] Konto M, Biu AA, Ahmed MI, Charles S. Prevalence and seasonal abundance of ticks on dogs and the role of *Rhipicephalus sanguineus* in transmitting *Babesia* species in Maidugiri, North-Eastern Nigeria. Vet World. 2014;7:119.

[208] Lawal MD, Ameh IG, Ahmed A. Some ectoparasites of *Camelus dromedarius* in Sokoto, Nigeria. J Entomol. 2007;4:143–8.

[209] Lawal MD, Mahmuda A, Fabiyi JP, George BD, Adamu Y, Kabir A, et al. A preliminary study on the monthly dynamics of cattle tick infestation in Sokoto Northwestern Nigeria. Nig J Anim Prod. 2017;44:296–300.

[210] Lee VH, Kemp GE, Madbouly MH, Moore DL, Causey OR, Casals J. Jos, a new tick-borne virus from Nigeria. Am J Vet Res. 1974;1:1165–7. 4416978

[211] Leeflang P, Pimentel WJ, Blotkamp J. The occurrence of *Haemobartonella canis* in Nigeria. Bull Epizoot Dis Afr. 1974;22:51–3. 4478986

[212] Lorusso V, Gruszka KA, Majekodunmi A, Igweh A, Welburn SC, Picozzi K. *Rickettsia africae* in *Amblyomma variegatum* ticks, Uganda and Nigeria. Emerg Infect Dis. 2013;19:1705. 24050756 10.3201/eid1910.130389 PMC3810746

[213] Lorusso V, Picozzi K, de Bronsvoort BM, Majekodunmi A, Dongkum C, Balak G, et al. Ixodid ticks of traditionally managed cattle in central Nigeria: where *Rhipicephalus* (*Boophilus*) *microplus* does not dare (yet?). Parasit Vectors. 2013;6:1–0. 23758913 10.1186/1756-3305-6-171 PMC3681633

[214] Ghamba PE, Goje LJ, Sheriff AB. Viral detection in ticks and the level of ticks’infestations amongst small ruminants in Maiduguri, Borno state. BIMA J Sci Technol GOMBE. 2022;6:125–34.

[215] Maidala AM. A survey of cattle, sheep and goat ticks infestation in Katagum local government area of Bauchi state, Nigeria. Int J Agric Earth Sci. 2015;1:1–5.

[216] Mamman AH, Lorusso V, Adam BM, Dogo GA, Bown KJ, Birtles RJ. First report of *Theileria annulata* in Nigeria: findings from cattle ticks in Zamfara and Sokoto states. Parasit Vectors. 2021;14:1–9. 33962649 10.1186/s13071-021-04731-4 PMC8105942

[217] Mbaya AW, Mohammed AM, Okwudiri NC, Isa IU. Captive wild animals as potential reservoirs of haemo and ectoparasitic infections of man and domestic animals in the aridregion of Northeastern Nigeria. Vet arhiv. 2008;78:429–40.

[218] Mohammed AN. Prevalence and experimental transmission of bovine piroplasms in northern Nigeria. Bull Anim Health Prod Afr. 1976;24:171–80. 16300138

[219] Mohammed AN. Seasonal incidence of Ixodid ticks of cattle in Northern Nigeria. Bull Anim Health Prod Afr. 1977;25:273–93.

[220] Mohammed AN, Aliu YO. Nymphae of *Amblyomma variegatum*, adults of *Aponomma latum* and *Haemogregarina* spp. from the African beauty snake *Psammorphis sibilans*. Niger J Sci. 1973;7:17–8.

[221] Musa HI, Jajere SM, Adamu NB, Atsanda NN, Lawal JR, Adamu SG, et al. Prevalence of tick infestation in different breeds of cattle in Maiduguri, Northeastern Nigeria. Bangladesh J Vet Med. 2014;12:161–6.

[222] Natala AJ, Balogun EO, Balogun JA, Inuwa HM, Nok AJ, Shiba T, et al. Identification and characterization of sialidase-like activity in the developmental stages of *Amblyomma variegatum*. J Med Entomol. 2013;50:85–93. 23427656 10.1603/me12152

[223] Natala AJ, Okubanjo OO, Ulayi BM, Owolabi YN, Jatau ID, Yusuf KH. Ectoparasites of domestic animals in Northern Nigeria. J Anim Plant Sci. 2009;3:238–42.

[224] Nduaka O, Ikeme MM. Human skin lesions in East Central state, Nigeria due to the larvae of *Amblyomma variegatum* (Fabricius, 1794). Niger Med J. 1973;3:140–3. 16366347

[225] Ndumu PA, George JB, Choudhury MK. Toxicity of neem seed oil (*Azadiracta indica*) against the larvae of *Amblyomma variegatum* a three-host tick in cattle. Phytother Res. 1999;13:532–4. 10479769 10.1002/(sici)1099-1573(199909)13:6<532::aid-ptr492>3.0.co;2-c

[226] Njila HL, Debi-Dore JD, Ombugadu A, Dibal M, Mafuyai MJ. Survey of ectoparasites infesting captive birds in the Jos museum zoological garden, North Central, Nigeria. J Nat Sci Res. 2018;8:36–40.

[227] Nwosu CO, Adamu M, Shinggu PA, Ahmed MI. Parasites of camels (*Camelus dromedarius*) in Borno state, Nigeria. Niger J Exp Appl Biol. 2003;4:65–70.

[228] Obadiah HI, Onah IE, Ugochukwu JU, Gbinde AK. Tick infestation of cattle in three markets in Makurdi, North-central, Nigeria. Am J Entomol. 2017;1:6–10.

[229] Obadiah HI, Shekaro A. Survey of tick infestation in cattle in Zaria abattoir Nigeria. J Vet Adv. 2012;2:81–7.

[230] Obeta SS, Ibrahim B, Lawal IA, Natala JA, Ogo NI, Balogun EO. Prevalence of canine babesiosis and their risk factors among asymptomatic dogs in the federal capital territory, Abuja, Nigeria. Parasite Epidemiol Control. 2020;11:e00186. 33102824 10.1016/j.parepi.2020.e00186 PMC7575870

[231] Oduguwa BO, Oloyo OO, Talabi AD, Sogunle OA, Okwelum N, Oloyo RA. Assessment of tick infestation and its effects on growth of extensively managed cattle in Ogun state, Nigeria. Niger Vet J. 2013;34:701–8.

[232] Ofukwu RA, Ogbaje CI, Akwuobu CA. Preliminary study of the epidemiology of ectoparasite infestation of goats and sheep in Makurdi, North Central Nigeria. Sokoto J Vet Sci. 2008;7:2.

[233] Ogo N, de Mera IG, Okubanjo O, de la Fuente J. Genetic characterization of *Coxiella burnetii* in *Amblyomma varigatum* ticks from North-central Nigeria: Public health importance. Infection. 2013;5:6.

[234] Ogo NI, de Mera IG, Galindo RC, Okubanjo OO, Inuwa HM, Agbede RI, et al. Molecular identification of tick-borne pathogens in Nigerian ticks. Vet Parasitol. 2012;187:572–7. 22326937 10.1016/j.vetpar.2012.01.029

[235] Ojeh CK, Dipeolu OO. The occurrence of *Aponoma ochraceum* (Acarina: Ixodidae) on a royal python in Ibadan, Nigeria. Niger Ent Mag. 1983;4:6–37.

[236] Okaeme AN. Ectoparasites and gastrointestinal parasites of nomadic cattle infiltrating into Kainji lake national park Nigeria. Int J Zoonoses. 1986;13:40–4. 3759355

[237] Okaeme AN. Ectoparasites of guinea fowl (*Numida meleagris galeata* Pallas) and local domestic chicken (*Gallus gallus*) in Southern Guinea Savanna, Nigeria. Vet Res Commun. 1988;12:277–80. 3195043 10.1007/BF00343245

[238] Okaeme AN. Lameness associated with heavy ectoparasitic infestation in *Numidia meleagris galeata*, *Gallus domestica*, *Pavo multicus*. Bull Anim Health Prod Afr. 1989;3:189–90.

[239] Okaeme AN, Osakwe ME. Ectoparasites of the African hedgehog *Atelerix albiventris*,(Wagner) in the Kainji lake area of Nigeria. Afr J Ecol. 1985;23:167–9.

[240] Okeke JJ, Ikegbunam NM, Umeaniebue AC, Ezeonyejiaku DC, Ezeadila JO. A survey on the ectoparasites and haemoparasites of grasscutter (*Thryonomys swinderianus*) reared under captive conditions. J Nat Sci Res. 2013;3:57–60.

[241] Okewole E, Adejinmi J. Comparison of two clinic-based immunoassays with the immunofluorescence antibody test for the field diagnosis of canine monocytic ehrlichiosis. Acta Microbiol Immunol Hung. 2009;56:145–55. 19621766 10.1556/AMicr.56.2009.2.3

[242] Okiwelu SN, Ikpamii T, Umeozor OC. Arthropods associated with mammalian carcasses in Rivers state, Nigeria. Afr J Biomed Res. 2008;11:339–42.

[243] Okoh AE, Oyetunde IL, Ibu JO. Fatal heartwater in a captive Sitatunga. Vet Rec. 1986;118:696. 3739182 10.1136/vr.118.25.696-a

[244] Okoli IC, Okoli CG, Opara M. Environmental and multi-host infestation of the brown dog tick, *Rhipicephalus sanguineus* in Owerri, South-east Nigeria- a case report. Vet Arh. 2006;76:93–100.

[245] Okon EO, Obiekazie AI. Parasites of cattle in Obudu cattle ranch. Niger Vet J. 1981;10:1–4.

[246] Okorafor KA, Odaibo AB, Eleng I, Okete JA. Occurrence and prevalence of ecto and gastrointestinal parasites in wild cane rats (*Tryonomys swinderianus*) from Oyo state, South-Western Nigeria. Eur J Res. 2012;1:70–6.

[247] Okwuonu ES, Andong FA, Ugwuanyi IK. Association of ticks with seasons, age, and cattle color of North-Western region of Nigeria. Sci Afr. 2021;12:e00832.

[248] Okwuonu ES, Bala AY, Ikpeze OO. Ixodid ticks infestation of zebu cattle crosses in Sokoto state Nigeria. Bioscientist. 2017;5:50–6.

[249] Olabode HO, Silas PM, Agbede RI. Survey of ectoparasites and their predilection sites on cattle in Bukuru Market. J Agric Vet Sci. 2010;2:70–4.

[250] Omonijo AO, Sowemimo OA. Prevalence of ectoparasites of dogs and cats in Ijero and Moba LGAs, Ekiti State, Nigeria. Niger J Parasitol. 2017;38:278–83.

[251] Omonona A, Adeyanju T, Eke F. Parasite prevalence among wildbirds in two sites in Ibadan, South Western Nigeria. J Prod Agric. 2014;10:65–77.

[252] Omudu EA, Amuta EU. Parasitology and urban livestock farming in Nigeria: prevalence of ova in faecal and soil samples and animal ectoparasites in Makurdi. J S Afr Vet Med Assoc. 2007;78:40–5. 10.4102/jsava.v78i1.285 17665765

[253] Omudu EA, Iorlaha GT, Adelusi S. Medically important arthropods infesting some exotic birds and mammals in the Makurdi zoological garden in Benue State, Nigeria. Sci J King Faisal Univ. 2011;12:1432.

[254] Onyali IO, Oluigbo FO, Ajayi ST. Dry season outbreaks of *Ornithodoros savignyi* in Gashu’a, Borno State: a case report. Trop Vet. 1989;7:101–3.

[255] Onyiche TE, Taioe MO, Ogo NI, Sivakumar T, Biu AA, Mbaya AW, et al. Molecular evidence of *Babesia caballi* and *Theileria equi* in equines and ticks in Nigeria: prevalence and risk factors analysis. Parasitology. 2020;147:1238–48. 32605687 10.1017/S0031182020000992 PMC10317755

[256] Onyiche TE, Ogo NI, Thekisoe O. Species distribution, prevalence, and risk factors associated with tick infestations of equines in Nigeria. Int J Acarol. 2022;48:201–6.

[257] Onyiche TE, Răileanu C, Tauchmann O, Fischer S, Vasić A, Schäfer M, et al. Prevalence and molecular characterization of ticks and tick-borne pathogens of one-humped camels (*Camelus dromedarius*) in Nigeria. Parasit Vectors. 2020;13:1–6. 32838795 10.1186/s13071-020-04272-2 PMC7445909

[258] Opara MN, Ezeh NO. Ixodid ticks of cattle in Borno and yours truly, Obe states of Northeastern Nigeria: breed and coat colour preference. Anim Res Int. 2011;8:1359–65.

[259] Opara MN, Abdu Y, Okoli IC. Survey of ticks of veterinary importance and tick-borne protozoa of cattle grazed in very hot months in Sokoto Municipality, Nigeria. Int J Agr Rural Dev. 2005;6:168–74.

[260] Opasina BA. Disease constraints on village goat production in Southwest Nigeria. Rev Elev Med Vet Pays Trop. 1985;38:284–94. 3841965

[261] Opasina BA, Dipeolu OO, Fagbemi BO. Some ectoparasites of veterinary importance on dwarf sheep and goats under traditional system of management in the humid forest and derived savanna zones of Nigeria. Rev Elev Méd Vét Pays Trop. 1983. 10.5555/19842243477. 6675076

[262] Orhierhor M, Okaka CE, Okonkwo VO. A survey of the parasites of the African white-bellied pangolin, *Phataginus tricuspis*, in Benin City, Edo State, Nigeria. Niger J Parasitol. 2017;38:266–70.

[263] Paul BT, Bello AM, Haruna NM, Dauda J, Ojo DT, Gadzama MA. Infestation of zebu cattle (*Bos indicus Linnaeus*) by hard ticks (Acari: Ixodidae) in Maiduguri, Northeastern Nigeria. Persian J Acarol. 2017;6:213–24.

[264] Pearse AS. Ecology of the ectoparasites of Nigerian rodents and insectivores. J Mammal. 1929;10:229–39.

[265] Philip CB. Discovery in West Africa of *Hunterbllus hookeri howard,* parasite of ixodids. Annls Parasitol. 1931;9:276.

[266] Philip CB. Occurrence of a colony of the tick parasite *Hunterellus hookeri Howard* in West Africa. Public Health Rep. 1931. 10.2307/4580173.

[267] Pullan NB. Productivity of white Fulani cattle on the Jos Plateau, Nigeria III Disease and management factors. Trop Anim Health Pro. 1980;12:77–84. 10.1007/BF02242612 7414701

[268] Reye AL, Arinola OG, Hübschen JM, Muller CP. Pathogen prevalence in ticks collected from the vegetation and livestock in Nigeria. Appl Environ Microbiol. 2012;78:2562–8. 22327584 10.1128/AEM.06686-11 PMC3318832

[269] Sadiq NA, Adejinmi JO, Adedokun OA, Fashanu SO, Alimi AA, Sofunmade YT. Ectoparasites and haemoparasites of indigenous chicken (*Gallus domesticus*) in Ibadan and environs. Trop Vet. 2003;21:187–91.

[270] Sadiq NA, Adejinmi JO, Adedokun AO. Anthropophilic nature of the brown dog tick, *Rhipicephalus sanguineus* in Ibadan, Nigeria. Trop Vet. 2001;19:58–9.

[271] Sa’idu L, Agbede RI, Abdu AP. Prevalence of avian spirochaetosis in Zaria (1980–1989). Isr J Vet Med. 1995;50:39–40.

[272] Sambo SJ, Ibrahim ND, Esievo KA, Hambolu JO, Oladele SB, Sackey AK, et al. Co-existence of besnoitiosis and dermatophilosis in indigenous cattle slaughtered at Zaria abattoir. J Anim Vet Adv. 2007;2:617–20.

[273] Sanusi M, Ahmed IA, Tahir I, Mai HM, Kalla DJ, Shuaibu I. Survey of equine piroplasmosis in the savanna areas, Bauchi state, north-eastern Nigeria. Ippologia. 2014;25:3–8.

[274] Shitta KB, James-Rugu NN, Badaki JA. Prevalence of ticks on dogs in Jos, Plateau state, Nigeria. Bayero J Pure Appl Sci. 2018;11:451–4.

[275] Simpson JJ. Entomological research in British West Africa II Northern Nigeria. Bull Ent Res. 1914;2:301–56.

[276] Simpson JJ. Entomological research in British West Africa III Southern Nigeria. Bull Ent Res. 1914;3:137–93.

[277] Tags SZ, Agbede RI, Mohammed BR. First incidence of ectoparasites in Abuja zoological parks, Abuja, Nigeria. Ann Parasitol. 2020;66:533–7. 33789025 10.17420/ap6604.295

[278] Takeet MI, Oyewusi IK, Ganiyu AI, Anifowose IO, Famuyide MI, Talabi OA, et al. Molecular detection of protozoan parasites in ticks infesting cattle entering Nigeria through a major trans-boundary route in Ogun state. Bull Anim Hlth Prod Afr. 2017;65:175–80.

[279] Tomlinson JA, Apanaskevich DA. Two new species of *Haemaphysalis* Koch, 1844 (Acari: Ixodidae) in the *H. (Rhipistoma*)* spinulosa* subgroup, parasites of carnivores and hedgehogs in Africa. Syst Parasitol. 2019;96:485–509. 31123875 10.1007/s11230-019-09860-0

[280] Tongjura JD, Amuga GA, Ombugadu RJ, Azamu Y, Mafuiya HB. Ectoparasites infesting livestock in three local government areas (LGAs) of Nasarawa State. Nigeria Sci World J. 2012;7:15–7.

[281] Ubah AS, Abalaka SE, Idoko IS, Obeta SS, Ejiofor CE, Mshelbwala PP, et al. Canine babesiosis in a male Boerboel: hematobiochemical and anatomic pathological changes in the cardiorespiratory and reproductive organs. Vet Anim Sci. 2019;7:100049. 32734071 10.1016/j.vas.2019.100049 PMC7386722

[282] Ugbomoiko US, Ariza L, Heukelbach J. Parasites of importance for human health in Nigerian dogs: high prevalence and limited knowledge of pet owners. BMC Vet Res. 2008;4:1–9. 19068110 10.1186/1746-6148-4-49 PMC2615757

[283] Ugbomoiko US, Obiamiwe BA. Distribution and incidence of ectoparasites on small mammals in a rainforest belt of Southern Nigeria. Angew Parasitol. 1991;32:143–8. 1928798

[284] Ugochukwu EI, Apeh AO. Prevalence of ectoparasites of small ruminants in Nsukka, Nigeria. Int J Zoonoses. 1985;12:313–7. 3836218

[285] Ugochukwu EI, Nnadozie CC. Ectoparasitic infestation of dogs in Bendel State Nigeria. Int J Zoonoses. 1985;12:308–12. 3836217

[286] Ugochukwu EI, Omije FA. Ectoparasitic fauna of poultry in Nsukka Nigeria. Int J Zoonoses. 1986;13:93–7. 3793395

[287] Umar YA, George BD, Ajanusi OJ. Survey of hard ticks (Ixodidae) infesting one-humped camels (*Camelus dromedarius*) in Kano State-Nigeria. Niger J Parasitol. 2011;32:61–6.

[288] Unsworth K. The ixodid parasites of cattle in Nigeria, with particular reference to the Northern territories. Ann Trop Med Parasitol. 1952;46:331–6. 13008364 10.1080/00034983.1952.11685538

[289] Whitaker JJO, Matthysse JG. Records of some ectoparasites from Nigeria. Entomol News. 1982;93:95–102.

[290] Williams RW, Causey OR, Kemp GE. Ixodid ticks from domestic livestock in Ibadan, Nigeria as carriers of viral agents. J Med Entomol. 1972;9:443–5. 5080431 10.1093/jmedent/9.5.443

[291] Aeschlimann A, Morel PC. *Boophilus geigyi* n. sp. (Acarina Ixodea) une nouvelle tique du bétail de l’Ouest Africain. Acta Trop. 1965;22:162–8. 14319774

[292] Balt DK, Chitimia-Dobler L, Matloa D, Oosthuysen M, Mumcuoglu KY, Mans BJ, et al. Integrative taxonomy and species delimitation of *Rhipicephalus turanicus* (Acari: Ixodida: Ixodidae). Int J Parasitol. 2020;50:577–94. 32592812 10.1016/j.ijpara.2020.04.005

[293] Baltazard M, Bahmanyar M, Mopidi G. *Ornithodorus erraticus* et fièvres récurrentes. Bull Soc Pathol Exot. 1950;43:595–601.

[294] Baylet R, Gilbert-Desvallons Y, Fichez, Berton, Vaillant. Syndromes pseudo-grippaux à Dakar fièvre Q. Bull Société de pathologie exotique et de ses filiales. 1958;66:289–95. 13585048

[295] Brès P, Cornet M, Robin Y. Bandia virus (IPD/A 611), a new arbovirus isolated in Senegal. Ann Inst Pasteur. 1967;113:739–47. 6062793

[296] Camicas JL. *Argas* (*Persicargas*) *streptopelia* (Ixodoidea: Argasidae) on migrating and resident doves in Senegal. Ann Entomol Soc Am. 1970;63:910. 5462282 10.1093/aesa/63.3.910

[297] Camicas JL, Wilson ML, Cornet JP, Digoutte JP, Calvo MA, Adam F, et al. Ecology of ticks as potential vectors of Crimean-Congo hemorrhagic fever virus in Senegal: epidemiological implications. In: Hemorrhagic fever with renal syndrome, tick-and mosquito-borne viruses. Cham: Springer; 1991.

[298] Chapman LE, Wilson ML, Hall DB, LeGuenno B, Dykstra EA, Ba K, et al. Risk factors for Crimean-Congo hemorrhagic fever in rural Northern Senegal. J Infect Dis. 1991;164:686–92. 1910069 10.1093/infdis/164.4.686

[299] Cornet JP. Contribution à l’étude des tiques (Acarina: Ixodina) vectrices du virus de la Fièvre Hémorragique de Crimee-Congo (CCHF), au Sénégal. 3-*Rhipicephalus guilhoni* Morel et Vassilliades, variation de la taille en fonction de la charge parasitaire. Conséquences épidémiologiques. Acarologia. 1997;38:39–41.

[300] Cornet JP, Zeller H, Camicas JL. Contribution à l’étude des tiques (Acarina: Ixodina) vectrices du virus de la fièvre hémorragique Crimée-Congo (CCHF) au Sénégal II: Biologie, aux stases preimaginales, de *Hyalomma marginatum rufipes*. Acarologia. 1995;36:293–5.

[301] Cornet JP, Zeller H, Ba K, Camicas JL, Gonzalez JP, Wilson ML. Contribution a l’études des tiques (Acarina: Ixodina) vectrices du virus de la fièvre hémorraggique Crimée-Congo (CCHF) au Sénégal. I: Analyse du parasitisme chez les petits rongeurs. Acarologia. 1995;36:287–92.

[302] Dahmani M, Davoust B, Sambou M, Bassene H, Scandola P, Ameur T, et al. Molecular investigation and phylogeny of species of the Anaplasmataceae infecting animals and ticks in Senegal. Parasit Vectors. 2019;12:1–5. 31640746 10.1186/s13071-019-3742-y PMC6805679

[303] Diatta G, Mediannikov O, Boyer S, Sokhna C, Bassène H, Fenollar F, et al. An alternative strategy of preventive control of tick-borne relapsing fever in rural areas of Sine-Saloum, Senegal. Am J Trop Med Hyg. 2016;95:537–45. 27430543 10.4269/ajtmh.15-0776 PMC5014255

[304] Duron O, Noël V, McCoy KD, Bonazzi M, Sidi-Boumedine K, Morel O, et al. The recent evolution of a maternally-inherited endosymbiont of ticks led to the emergence of the Q fever pathogen, *Coxiella burnetii*. PLoS Pathog. 2015;11:e1004892. 25978383 10.1371/journal.ppat.1004892 PMC4433120

[305] Faye O, Cornet JP, Camicas JL, Fontenille D, Gonzalez JP. Transmission expérimentale du virus de la fièvre hémorragique de Crimée-Congo: Place de trois espèces vectrices dans les cycles de maintenance et de transmission au Sénégal. Parasite. 1999;6:27–32. 10229934 10.1051/parasite/1999061027

[306] Grard G, Lemasson JJ, Sylla M, Dubot A, Cook S, Molez JF, et al. Ngoye virus: a novel evolutionary lineage within the genus *Flavivirus*. J Gen Virol. 2006;87:3273–7. 17030860 10.1099/vir.0.82071-0

[307] Gueye A, Mbengue M, Dieye T, Diouf A, Seye M, Seye MH. Cowdriosis in Senegal: some epidemiological aspects. Rev Elev Med Vet Pays Trop. 1993;46:217. 8134635

[308] Gueye A, Mbengue M, Diouf A. Tiques et hemoparasitoses du betail au Senegal. III. La zone nord-soudanienne. Rev Elev Med Vet Pays Trop. 1989;42:411–20. 2485548

[309] Gueye A, Mbengue M, Diouf A. Tiques et hemoparasitoses du betail au Senegal IV La zone sud-soudanienne. Rev Élev Méd Vét Pays Trop. 1989;42:517–28. 2218038

[310] Gueye A, Mbengue MB, Diouf A. Tiques et hémoparasitoses du bétail au Sénégal. VI. La zone soudano-sahélienne. Rev Elev Med Vet Pays Trop. 1994;47:39–46. 7991897

[311] Gueye A, Mbengue M, Diouf A, Sonko ML. Tiques et hémoparasitoses du bétail au Sénégal. V. La zone nord-guinéenne. Rev Elev Med Vet Pays Trop. 1993;46:551–61. 8073170

[312] Gueye A, Mbengue M, Diouf A, Seye M. Tiques et hemoparasitoses du betail au Senegal. I. La region des Niayes. Rev Elev Med Vet Pays Trop. 1986;39:381–93. 3659490

[313] Gueye A, Mbengue M, Diouf A, Vassiliades G. Prophylaxie de la cowdriose et observations sur la pathologie ovine dans la région des Niayes au Sénégal. Rev Elev Med Vet Pays Trop. 1989;42:497–503. 2218032

[314] Hoogstraal H, El Kammah KM. Notes on African *Haemaphysalis *ticks. X. H. (Kaiseriana) aciculifer Warburton and H. (K.) rugosa Santos Dias, the African representatives of the spinigera subgroup (Ixodea: Ixodidae). J Parasitol. 1972;58:960–78. 5078603

[315] Keita AK, Mediannikov O, Ratmanov P, Diatta G, Bassene H, Roucher C, et al. Looking for *Tropheryma whipplei* source and reservoir in rural Senegal. Am J Trop Med Hyg. 2013;88:339. 23249690 10.4269/ajtmh.2012.12-0614 PMC3583327

[316] Logan TM, Wilson ML, Cornet JP. Association of ticks (Acari: Ixodoidea) with rodent burrows in northern Senegal. J Med Entomol. 1993;30:799–801. 8360905 10.1093/jmedent/30.4.799

[317] Main AJ, Kloter KO, Camicas JL, Robin Y, Sarr M. Wad medani and soldado viruses from ticks (Ixodoidea) in West Africa. J Med Entomol. 1980;17:380–2.

[318] Mangombi JB, Roqueplo C, Sambou M, Dahmani M, Mediannikov O, Comtet L, et al. Seroprevalence of Crimean-Congo hemorrhagic fever in domesticated animals in northwestern Senegal. Vector-Borne Zoonotic Dis. 2020;20:797–9. 32429789 10.1089/vbz.2019.2592

[319] Mediannikov O, Diatta G, Fenollar F, Sokhna C, Trape JF, Raoult D. Tick-borne rickettsioses, neglected emerging diseases in rural Senegal. PLoS Negl Trop Dis. 2010;4:e821. 20856858 10.1371/journal.pntd.0000821 PMC2939048

[320] Mediannikov O, Diatta G, Kasongo K, Raoult D. Identification of Bartonellae in the soft tick species *Ornithodoros sonrai* in Senegal. Vector-Borne Zoonotic Dis. 2014;14:26–32. 24359424 10.1089/vbz.2013.1326 PMC3880920

[321] Mediannikov O, Karkouri KE, Diatta G, Robert C, Fournier PE, Raoult D. Non-contiguous finished genome sequence and description of *Bartonella senegalensis* sp. nov. Stand Genom Sci. 2013;8:279–89. 10.4056/sigs.3807472 PMC3746424 23991259

[322] Mediannikov O, Fenollar F, Socolovschi C, Diatta G, Bassene H, Molez JF, et al. *Coxiella burnetii* in humans and ticks in rural Senegal. PLoS Negl Trop Dis. 2010;4:e654. 20386603 10.1371/journal.pntd.0000654 PMC2850317

[323] Mediannikov O, Nguyen TT, Bell-Sakyi L, Padmanabhan R, Fournier PE, Raoult D. High quality draft genome sequence and description of *Occidentia massiliensis* gen. nov., sp. nov., a new member of the family Rickettsiaceae. Stand Genomic Sci. 2014;9:1–8. 25780502 10.1186/1944-3277-9-9 PMC4334944

[324] Mediannikov O, Socolovschi C, Edouard S, Fenollar F, Mouffok N, Bassene H, et al. Common epidemiology of *Rickettsia felis* infection and malaria, Africa. Emerg Infect Dis. 2013;19:1775. 24188709 10.3201/eid1911.130361 PMC3837673

[325] Mezo RC, García JL, Arroyo M, Fleury A. Relevance of 3D magnetic resonance imaging sequences in diagnosing basal subarachnoid neurocysticercosis. Acta Trop. 2015;152:60–5. 26327445 10.1016/j.actatropica.2015.08.017

[326] Ndiaye EH, Diouf FS, Ndiaye M, Bassene H, Raoult D, Sokhna C, et al. Tick-borne relapsing fever Borreliosis, a major public health problem overlooked in Senegal. PLoS Negl Trop Dis. 2021;15:e0009184. 33886571 10.1371/journal.pntd.0009184 PMC8096072

[327] Parent R, Alogninouwa T. Amélioration de la productivité de l’élevage en zone tropicale. Traitement systématique des vaches gestantes à l’Ivermectine dans les mois précédant la mise bas. Rev Elev Med Vet Pays Trop. 1984;37:341–54.

[328] René-Martellet M, Minard G, Massot R, Van Tran V, Valiente Moro C, Chabanne L, et al. Bacterial microbiota associated with *Rhipicephalus sanguineus* (sl) ticks from France Senegal and Arizona. Parasit Vect. 2017;10:1. 10.1186/s13071-017-2352-9 PMC5591579 28886749

[329] Robin Y, Camicas JL, Bres P, Hery G. International symposium on tick-borne arboviruses (excluding group B). Observations on some viruses isolated from ticks in Senegal. Folia Parasitol. 1970;17:345–8.

[330] Sambou M, Faye N, Bassène H, Diatta G, Raoult D, Mediannikov O. Identification of rickettsial pathogens in ixodid ticks in northern Senegal. Ticks Tick-borne Dis. 2014;5:552–6. 24908548 10.1016/j.ttbdis.2014.04.002

[331] Sylla M, Pourrut X, Faye N, Ba K, Cornet JP, Camicas JL. Argasidae (Acari: Ixodida) parasites of wild and domestic animals in Senegal: 1-review and distribution. Acarologia. 2004;44:137–49.

[332] Sylla M, Thonnon J. Argasidae (Acari: Ixodida) parasites of wild and domestic animals in Senegal: 2-arboviruses isolation and epidemiological implications. Acarologia. 2004;44:151–6.

[333] Sylla M, Molez JF, Cornet JP, Camicas JL. Climate change and distribution of ticks (Acari: Ixodida) in Senegal and Mauritania. Acarologia. 2009;48:137–53.

[334] Sylla M, Molez JF, Cornet JP, Mondet B, Camicas JL. Les tiques (Acari: Ixodida) du Sénégal: Fréquence des hôtes répertoriés, dynamique saisonnière et chorologie d’*Amblyomma* (Xiphiastor) *variegatum* (Fabricius, 1794). Acarologia. 2006;47:13–23.

[335] Trape JF, Godeluck B, Diatta G, Rogier C, Legros F, Albergel J, et al. The spread of tick-borne borreliosis in West Africa and its relationship to sub-Saharan drought. Am J Trop Med Hyg. 1996;54:289–93. 8600768 10.4269/ajtmh.1996.54.289

[336] Trape JF, Diatta G, Arnathau C, Bitam I, Sarih MH, Belghyti D, et al. The epidemiology and geographic distribution of relapsing fever borreliosis in West and North Africa, with a review of the *Ornithodoros erraticus* complex (Acari: Ixodida). PLoS ONE. 2013;8:e7847. 10.1371/journal.pone.0078473 PMC3817255 24223812

[337] Vial L, Diatta G, Tall A, Bouganali H, Durand P, Sokhna C, et al. Incidence of tick-borne relapsing fever in west Africa: longitudinal study. Lancet. 2006;368:37–43. 16815378 10.1016/S0140-6736(06)68968-X

[338] Vial L, Durand P, Arnathau C, Halos L, Diatta G, Trape JF, et al. Molecular divergences of the *Ornithodoros sonrai* soft tick species, a vector of human relapsing fever in West Africa. Microbes Infect. 2006;8:2605–11. 16962358 10.1016/j.micinf.2006.07.012

[339] Vial L, Wieland B, Jori F, Etter E, Dixon L, Roger F. African swine fever virus DNA in soft ticks, Senegal. Emerg Infect Dis. 2007;13:1928. 18258050 10.3201/eid1312.071022 PMC2876773

[340] Zeller HG, Cornet JP, Camicas JL. Experimental transmission of Crimean-Congo hemorrhagic fever virus by west African wild ground-feeding birds to *Hyalomma marginatum rufipes* ticks. Am J Trop Med Hyg. 1994;50:676–81. 8024058 10.4269/ajtmh.1994.50.676

[341] Zeller HG, Cornet JP, Diop A, Camicas JL. Crimean—congo hemorrhagic fever in ticks (Acari: Ixodidae) and ruminants: field observations of an epizootic in Bandia, Senegal (1989–1992). J Med Entomol. 1997;34:511–6. 9379454 10.1093/jmedent/34.5.511

[342] Kamara JA. Some parasites of wild animals in Sierra Leone. Bull Animal Health Production Afr. 1973;2:265–8.

[343] Myers BJ, Kuntz RE, Kamara JA. Parasites and commensals of chimpanzees captured in Sierra-Leone West-Africa. P Helm Soc Wash. 1973;40:298–9.

[344] Nuttall GH, Cuncliffe N. Notes on ticks. III: on four new species of *Ixodes*. Parasitology. 1913;6:131–8.

[345] Simpson JJ. Entomological research in British West Africa. IV. Sierra Leone. Bull Entomol Res. 1913;4:151–90.

[346] Walton GA. The *Ornithodorus moubata* superspecies problem in relation to human relapsing fever epidemiology. Symp Zool Soc London. 1962;6:83–156.

[347] Yorke W, Blacklock B. Notes on certain animal parasites of domestic stock in Sierra Leone. Ann Trop Med Parasitol. 1915;9:413–20.

[348] Alimi D, Hajri A, Jallouli S, Sebai H. In vitro acaricidal activity of essential oil and crude extracts of *Laurus nobilis*, (Lauraceae) grown in Tunisia, against arthropod ectoparasites of livestock and poultry: *Hyalomma scupense* and *Dermanyssus gallinae*. Vet Parasitol. 2021;298:109507. 34388421 10.1016/j.vetpar.2021.109507

[349] Alimi D, Hajri A, Jallouli S, Sebai H. Phytochemistry, anti-tick, repellency and anti-cholinesterase activities of *Cupressus sempervirens L.* and *Mentha pulegium L.* combinations against *Hyalomma scupense* (Acari: Ixodidae). Vet Parasitol. 2022;303:109665. 35134594 10.1016/j.vetpar.2022.109665

[350] Belkahia H, Said MB, El Hamdi S, Yahiaoui M, Gharbi M, Daaloul-Jedidi M, et al. First molecular identification and genetic characterization of *Anaplasma ovis* in sheep from Tunisia. Small Rumin Res. 2014;121:404–10.

[351] Bensaoud C, Abdelkafi-Koubaa Z, Ben Mabrouk H, Morjen M, Hmila I, Rhim A, et al. *Hyalomma dromedarii* (Acari: Ixodidae) salivary gland extract inhibits angiogenesis and exhibits in vitro antitumor effects. J Med Entomol. 2017;54:1476–82. 29029126 10.1093/jme/tjx153

[352] Benyedem H, Lekired A, Mhadhbi M, Dhibi M, Romdhane R, Chaari S, et al. First insights into the microbiome of Tunisian *Hyalomma* ticks gained through next-generation sequencing with a special focus on *H. scupense*. PLoS ONE. 2022;17:e0268172. 35587930 10.1371/journal.pone.0268172 PMC9119559

[353] Bouattour A, Darghouth MA, Miled LB. Cattle infestation by *Hyalomma* ticks and prevalence of *Theileria* in *H. detritum* species in Tunisia. Vet Parasitol. 1996;65:233–45. 8983149 10.1016/s0304-4017(96)00951-x

[354] Chastel C, Bach-Hamba D, Karabatsos N, Bouattour A, Le Lay G, Le Goff F, et al. Tunis virus: a new Phlebovirus from *Argas reflexus hermanni* ticks in Tunisia. Acta Virol. 1994;38:285–9. 7726003

[355] Demoncheaux JP, Socolovschi C, Davoust B, Haddad S, Raoult D, Parola P. First detection of *Rickettsia aeschlimannii* in *Hyalomma dromedarii* ticks from Tunisia. Ticks Tick-borne Dis. 2012;3:398–402. 23182544 10.1016/j.ttbdis.2012.10.003

[356] Dsouli N, Younsi-Kabachii H, Postic D, Nouira S, Gern L, Bouattour A. Reservoir role of lizard *Psammodromus algirus* in transmission cycle of *Borrelia burgdorferi* sensu lato (Spirochaetaceae) in Tunisia. J Med Entomol. 2006;43:737–42. 16892633 10.1603/0022-2585(2006)43[737:rrolpa]2.0.co;2

[357] Elati K, Bouaicha F, Dhibi M, Smida BB, Mhadhbi M, Obara I, et al. Phenology and phylogeny of *Hyalomma *spp. ticks infesting one-humped camels (*Camelus dromedarius*) in the Tunisian Saharan bioclimatic zone. Parasite. 2021;28:44. 34009121 10.1051/parasite/2021038 PMC8132600

[358] Fares W, Dachraoui K, Cherni S, Barhoumi W, Slimane TB, Younsi H, et al. Tick-borne encephalitis virus in *Ixodes ricinus* (Acari: Ixodidae) ticks. Tunisia Ticks Tick-Borne Dis. 2021;12:101606. 33189912 10.1016/j.ttbdis.2020.101606

[359] Fares W, Dachraoui K, Najjar C, Younsi H, Findlay-Wilson S, Petretto M, et al. Absence of Crimean-Congo haemorrhagic fever virus in the tick *Hyalomma aegyptium* parasitizing the spur-thighed tortoise (*Testudo graeca*) in Tunisia. Parasite. 2019;26:35. 31198174 10.1051/parasite/2019036 PMC6568017

[360] Gharbi M, Drissi G, Darghouth MA. Population dynamics of ticks infesting horses in North-west Tunisia Revue Scientifique et Technique (International Office of Epizootics). Vet Parasitol. 2018;37:837–41. 10.20506/rst.37.3.2890 30964464

[361] Gharbi M, Hayouni ME, Sassi L, Dridi W, Darghouth MA. *Hyalomma scupense* (Acari, Ixodidae) in Northeast Tunisia: seasonal population dynamics of nymphs and adults on field cattle. Parasite. 2013;20:12. 23547915 10.1051/parasite/2013012 PMC3718523

[362] Gharbi M, Moussi N, Jedidi M, Mhadhbi M, Sassi L, Darghouth MA. Population dynamics of ticks infesting the one-humped camel (*Camelus dromedarius*) in central Tunisia. Ticks Tick-borne Dis. 2013;4:488–91. 23999226 10.1016/j.ttbdis.2013.06.004

[363] Gharbi M, Rjeibi MR, Rouatbi M, Mabrouk M, Mhadhbi M, Amairia S, et al. Infestation of the spur-thighed tortoise (*Testudo graeca)* by *Hyalomma aegyptium* in Tunisia. Ticks Tick-borne Dis. 2015;6:352-5. 10.1016/j.ttbdis.2015.02.009. 25791981 10.1016/j.ttbdis.2015.02.009

[364] Gharbi M, Sassi L, Dorchies P, Darghouth MA. Infection of calves with *Theileria annulata* in Tunisia: economic analysis and evaluation of the potential benefit of vaccination. Vet Parasitol. 2006;137:231–41. 16481113 10.1016/j.vetpar.2006.01.015

[365] Khrouf F, M’Ghirbi Y, Znazen A, Ben Jemaa M, Hammami A, Bouattour A. Detection of *Rickettsia* in *Rhipicephalus sanguineus* ticks and *Ctenocephalides felis* fleas from southeastern Tunisia by reverse line blot assay. J Clin Microbiol. 2014;52:268–74. 24226919 10.1128/JCM.01925-13 PMC3911462

[366] Meeüs TD, Béati L, Delaye C, Aeschlimann A, Renaud F. Sex-biased genetic structure in the vector of Lyme disease, *Ixodes ricinus*. Evolution. 2002;56:1802–7. 12389725 10.1111/j.0014-3820.2002.tb00194.x

[367] Rjeibi MR, Amairia S, Mhadhbi M, Rekik M, Gharbi M. Detection and molecular identification of *Anaplasma phagocytophilum* and *Babesia* spp. infections in *Hyalomma aegyptium* ticks in Tunisia. Arch Microbiol. 2022;204:385. 35689686 10.1007/s00203-022-02995-7

[368] Rjeibi MR, Darghouth MA, Rekik M, Amor B, Sassi L, Gharbi M. First molecular identification and genetic characterization of *Theileria lestoquardi* in sheep of the Maghreb region. Transbound Emerg Dis. 2016;63:278–84. 25208526 10.1111/tbed.12271

[369] Rjeibi MR, Gharbi M, Mhadhbi M, Mabrouk W, Ayari B, Nasfi I, et al. Prevalence of piroplasms in small ruminants in North-West Tunisia and the first genetic characterization of *Babesia ovis* in Africa. Parasite. 2014;21:23. 24849588 10.1051/parasite/2014025 PMC4029076

[370] Ros-García A, M’ghirbi Y, Bouattour A, Hurtado A. First detection of *Babesia occultans* in *Hyalomma* ticks from Tunisia. Parasitology. 2011;138:578–82. 21284911 10.1017/S0031182011000060

[371] Said MB, Belkahia H, Alberti A, Zobba R, Bousrih M, Yahiaoui M, et al. Molecular survey of *Anaplasma* species in small ruminants reveals the presence of novel strains closely related to *A. phagocytophilum* in Tunisia. Vector-Borne Zoonot Dis. 2015;15:580–90. 10.1089/vbz.2015.1796 PMC4593892 26394065

[372] Said MB, Galai Y, Canales M, Nijhof AM, Mhadhbi M, Jedidi M, et al. Hd86, the Bm86 tick protein ortholog in *Hyalomma scupense* (syn. *H. detritum*): expression in *Pichia pastoris* and analysis of nucleotides and amino acids sequences variations prior to vaccination trials. Vet Parasitol. 2012;183:215–23. 21871736 10.1016/j.vetpar.2011.07.049

[373] Said MB, Galai Y, Mhadhbi M, Jedidi M, de la Fuente J, Darghouth MA. Molecular characterization of Bm86 gene orthologs from *Hyalomma excavatum*, *Hyalomma dromedarii* and *Hyalomma marginatum marginatum* and comparison with a vaccine candidate from *Hyalomma scupense*. Vet Parasitol. 2012;190:230–40. 22683299 10.1016/j.vetpar.2012.05.017

[374] Said Y, Lahmar S, Dhibi M, Rjeibi MR, Jdidi M, Gharbi M. First survey of ticks, tick-borne pathogens (*Theileria*, *Babesia*, *Anaplasma* and *Ehrlichia*) and *Trypanosoma evansi* in protected areas for threatened wild ruminants in Tunisia. Parasitol Int. 2021;81:102275. 33348021 10.1016/j.parint.2020.102275

[375] Seddik MM, Cuaquil L, Driot C, Khorchani T. Effects of herding methods on the kinetics of tick infestation of dromedary camel in southern Tunisia. Bull De La Soc Zool De France. 2011;136:299–311.

[376] Selmi R, Said MB, Mamlouk A, Yahia HB, Messadi L. Molecular detection and genetic characterization of the potentially pathogenic *Coxiella burnetii* and the endosymbiotic *Candidatus Midichloria mitochondrii* in ticks infesting camels (*Camelus dromedarius*) from Tunisia. Microb Pathog. 2019;136:103655. 31398530 10.1016/j.micpath.2019.103655

[377] Sfar N, M’ghirbi Y, Letaief A, Parola P, Bouattour A, Raoult D. First report of *Rickettsia monacensis* and *Rickettsia helvetica* from Tunisia. Ann Trop Med Parasitol. 2008;102:561–4. 18782496 10.1179/136485908X311795

[378] Younsi H, Fares W, Cherni S, Dachraoui K, Barhoumi W, Najjar C, et al. *Ixodes inopinatus* and *Ixodes ricinus* (Acari: Ixodidae) are sympatric ticks in North Africa. J Med Entomol. 2020;57:952–6. 31751458 10.1093/jme/tjz216

[379] Younsi H, Postic D, Baranton G, Bouattour A. High prevalence of *Borrelia lusitaniae* in *Ixodes ricinus* ticks in Tunisia. Eur J Epidemiol. 2001;17:53–6. 11523576 10.1023/a:1010928731281

[380] Zhioua E, Bouattour A, Hu CM, Gharbi M, Aeschliman A, Ginsberg HS, et al. Infection of *Ixodes ricinus* (Acari: Ixodidae) by *Borrelia burgdorferi* sensu lato in North Africa. J Med Entomol. 1999;36:216–8. 10083761 10.1093/jmedent/36.2.216

[381] Zhioua E, Gern L, Aeschlimann A. Isolement d’un spirochète à partir d’*Ixodes ricinus* de Tunisie. Soc Fr Parasitol. 1989;7:107–10.

[382] Bencherif S, Dahmani MB, Burgas D, Manzano P. Current social and rangeland access trends among pastoralists in the western Algerian steppe. Land. 2021;26:674.

[383] Bencherif, S., & Manzano, P. Intensification of pastoralism as a driver of degradation in the Algerian steppe. International Grassland Congress Proceedings. 2021.

[384] Siad O, Deghnouche K, Andrighetto I, Contiero B, Marchesini G, Bedjaoui H, et al. Traits of intensive livestock systems in Algerian steppe territories. Ital J Anim Sci. 2022;21:41–50.

[385] International Trade Administration. Morocco - Country Commerical Guides. 2024. https://www.trade.gov/country-commercial-guides/morocco-agricultural-sector Accessed 3 May 2024.

[386] Marx E. The ecology and politics of nomadic pastoralists in the middle east. De Gryter Mouton. 1978. 10.1515/9783110810233.41.

[387] Eljechtimi A. Morocco set to hit record tourism arrivals despite quake and Gaza war. 2023. https://www.reuters.com/world/africa/morocco-hit-record-tourism-arrivals-despite-headwinds-2023-12-18/ Accessed: 3 May 2024.

[388] Yuasa Y, Tsai YL, Chang CC, Hsu TH, Chou CC. The prevalence of *Anaplasma platys* and a potential novel *Anaplasma* species exceed that of *Ehrlichia canis* in asymptomatic dogs and *Rhipicephalus sanguineus* in Taiwan. J Vet Med Sci. 2017;79:1494–502. 28781347 10.1292/jvms.17-0224 PMC5627318

[389] Ullah R, Shams S, Khan MA, Ayaz S, Akbar NU, Din QU, et al. Epidemiology and molecular characterization of *Theileria annulata* in cattle from Central Khyber Pakhtunkhwa, Pakistan. PLoS ONE. 2021;16:e0249417. 34529664 10.1371/journal.pone.0249417 PMC8445462

[390] Muhammad KA, Gadzama UN, Onyiche TE. Distribution and prevalence of *Coxiella burnetii* in animals, humans, and ticks in Nigeria: a systematic review. Infect Dis Rep. 2023;15:576–88. 37888137 10.3390/idr15050056 PMC10606657

[391] Temur AI, Kuhn JH, Pecor DB, Apanaskevich DA, Keshtkar-Jahromi M. Epidemiology of Crimean-Congo hemorrhagic fever (CCHF) in Africa—underestimated for decades. Am J Trop Med Hyg. 2021;104:1978–90. 33900999 10.4269/ajtmh.20-1413 PMC8176481

[392] Blench R, Chapman R, & Slaymaker T. A study of the role of livestock in Poverty Reduction Strategy Papers (PRSPs). 2003. 10.22004/ag.econ.23776

[393] FAO. FAO in Nigeria: Nigeria at a glance. (n.d) https://www.fao.org/nigeria/fao-in-nigeria/nigeria-at-a-glance/en/​ Accessed 3 May 2024.

[394] Eeswaran R, Nejadhashemi AP, Faye A, Min D, Prasad PV, Ciampitti IA. Current and future challenges and opportunities for livestock farming in West Africa: perspectives from the case of Senegal. Agronomy. 2022;12:1818.

[395] Sambou M, Faye N, Bassène H, Diatta G, Raoult D, Mediannikov O. Identification of rickettsial pathogens in ixodid ticks in northern Senegal. Ticks Tick Borne Dis. 2015;5:552–6. 10.1016/j.ttbdis.2014.04.002 24908548

[396] Spickler AR. *Amblyomma variegatum*. 2022. http://www.cfsph.iastate.edu/DiseaseInfo/ factsheets.php Accessed 4 May 2024. ​

[397] Spickler AR. *Rhipicephalus* (Boophilis) *annulatus*. 2022. http://www.cfsph.iastate.edu/DiseaseInfo/factsheets.php Accessed 4 May 2024. ​​

[398] Dantas-Torres F. Biology and ecology of the brown dog tick, *Rhipicephalus sanguineus*. Parasit Vectors. 2010;3:26. 20377860 10.1186/1756-3305-3-26 PMC2857863

[399] Hassan IC. Animal health survey of cattle for the Bombali region, Sierra Leone, 1977. Beitrage zur Trop Land Vet. 1979;17:91–4. 533522

[400] O’Hearn AE, Voorhees MA, Fetterer DP, Wauquier N, Coomber MR, Bangura J, et al. Serosurveillance of viral pathogens circulating in West Africa. Virol J. 2016;13:163. 27716429 10.1186/s12985-016-0621-4 PMC5048616

[401] Redus MA, Parker RA, McDade JE. Prevalence and distribution of spotted fever and typhus infections in Sierra Leone and Ivory Coast. Int J Zoonoses. 1986;13:104–11. 3025125

[402] Rahman Sesay A. Review of the livestock/meat and milk value chains and policy influencing them in Sierra Leone. Food and Agriculture Organization of the United Nations and Economic Community of West African States. 2016. https://www.fao.org/documents/card/fr/c/87ed4679-429f-4d1f-958a-6a0ed5ce7a63/ Accessed 3 May 2024.

[403] FAO. FAO in Sierra Leone. (n.d.) https://www.fao.org/sierra-leone/fao-in- Accessed 2 May 2024.

[404] Elati K, Salhi I, Kodia R, Rekik M, Gharbi M. Epidemiological situation of bovine tropical theileriosis in an arid region in Central Tunisia with a phylogenetic analysis of *Theileria annulata*. Vet Med Sci. 2023;9:2862–70. 37725348 10.1002/vms3.1276 PMC10650360

[405] Selmi R, Mamlouk A, Ben Yahia H, Abdelaali H, Ben Said M, Sellami K, et al. *Coxiella burnetii* in Tunisian dromedary camels (*Camelus dromedarius*): seroprevalence, associated risk factors and seasonal dynamics. Acta Trop. 2018;188:234–9. 30219555 10.1016/j.actatropica.2018.09.008

[406] Zouaghi K, Bouattour A, Aounallah H, Surtees R, Krause E, Michel J, et al. First serological evidence of Crimean-Congo hemorrhagic fever virus and Rift Valley fever virus in ruminants in Tunisia. Pathogens. 2021;10:769. 34207423 10.3390/pathogens10060769 PMC8234966

[407] Bouaicha F, Eisenbarth A, Elati K, Schulz A, Ben Smida B, Bouajila M, et al. Epidemiological investigation of Crimean-Congo haemorrhagic fever virus infection among the one-humped camels (*Camelus dromedarius*) in southern Tunisia. Ticks Tick Borne Dis. 2021;12:101601. 33176235 10.1016/j.ttbdis.2020.101601

[408] Khamassi Khbou M, Romdhane R, Bouaicha Zaafouri F, Bouajila M, Sassi L, Appelberg SK, et al. Presence of antibodies to Crimean Congo haemorrhagic fever virus in sheep in Tunisia, North Africa. Vet Med Sci. 2021;7:2323–9. 34390548 10.1002/vms3.597 PMC8604105

[409] Guglielmone AA, Robbins RG, Apanaskevich DA, Petney TN, Estrada-Pena A, Horak IG. The hard ticks of the world. Acari: Ixodida: Ixodidae. Dordrecht: Springer; 2014.

[410] Estrada-Pena A, Mangold AJ, Nava S, Venzal JM, Labruna M, Guglielmone AA. A review of the systematic of the tick family Argasidae (Ixodida). Acarologia. 2010;50:317–33.

