# Supplementary material for: A compilation of ticks and tick-borne pathogen distributions in seven countries within North and West Africa from 1901 to 2022: a systematic literature review
Source: Parasit Vectors. 2025 Dec 4;19:13. doi: 10.1186/s13071-025-07153-8 (PMC12781586; doi:10.1186/s13071-025-07153-8)
Supplement: Supplementary file 3 — Additional file 3. Article List.docx [file 13071_2025_7153_MOESM3_ESM.docx]

**Additional file 3: Table S1: Comprehensive list of all articles extracted from this study.**

| Citation | Country/Countries |
| --- | --- |
| Abdulkareem BO, Christy AL, & Samuel UU. Prevalence of ectoparasite infestations in owned dogs in Kwara State, Nigeria. Parasite Epidemiology Control. 2019;4:e00079. | Nigeria |
| Abdullahi YM, Magami IM, Audu A & Mainasara MM. Prevalence of Ticks on Camels and Cattle Brought to Dodoru Market Kebbi State, Nigeria. Path of Science. 2018;4:3001-3004. | Nigeria |
| Abiola FA, Karimou M, & Houeto P. Cholinesterase activity in bovine ticks and its inhibition in vitro by organophosphate acaricides. Revue de Médecine Vétérinaire. 1991;142:147-152. | Niger |
| Adamu M, Troskie M, Oshadu DO, Malatji DP, Penzhorn BL, & Matjila PT. Occurrence of tick-transmitted pathogens in dogs in Jos, Plateau State, Nigeria. Parasites & Vectors. 2014;7:1-8. | Nigeria |
| Adamu NB, Adamu JY, & Salisu L. Prevalence of ecto-, endo-and haemoparasites in slaughtered dogs in Maiduguri, Nigeria. Revue de Médecine Vétérinaire. 2012;163:178-82. | Nigeria |
| Adang KL, Ayuba J, & Yoriyo KP. Ectoparasites of sheep (*Ovis aries* L.) and goats (*Capra hirus* L.) in Gombe, Gombe State, Nigeria. Pakistan Journal of Biological Sciences. 2015;18:224-31. | Nigeria |
| Adang LK, Oniye SJ, Ezealor AU, Abdu PA, Ajanusi JO, & Yoriyo KP. Ectoparasites of the Laughing Dove *Streptopelia senegalensis* (Linnaeus, 1766)(Aves: Columbidae) in Zaria, Nigeria. Lundiana International Journal of Biodiversity. 2008;9:67-71. | Nigeria |
| Adejoh VA, Pam VA, Uzoigwe NR, Naphtali RS, Yohanna JA, Pam RG, et al. Prevalence of hard ticks infesting cattle in Lafia, Nasarawa State, North Central Nigeria. Journal of Agricultural Research and Natural Resources. 2019;3:1-19. | Nigeria |
| Adelusi SM, Vajime CG, Omudu EA, Okpotu RO, Onazi FO. Avian Ectoparasitism in Makurdi, Nigeria: Do Wild Birds Serve as Reservior for Domestic Birds?. Nigerian Annals of Pure and Applied Sciences. 2015;6:11-5. | Nigeria |
| Ademola IO, & Akinboade OA. Increase exposure of *Rhipicephalus* (Boophilus) *decoloratus* (Koch, 1844)(Acarina: Ixodidae) to ultraviolet radiation affects its reproductive capacity. International Journal of Acarology. 2016;42:412-5. | Nigeria |
| Adenubi OT, Abolaji AO, Salihu T, Akande FA, & Lawal H. Chemical composition and acaricidal activity of Eucalyptus globulus essential oil against the vector of tropical bovine piroplasmosis, *Rhipicephalus* (Boophilus) *annulatus*. Experimental & Applied Acarology. 2021;83:301-12. | Nigeria |
| Adeyefa CA, & Dipeolu OO. Ectoparasites of horses in south-western Nigeria. International Journal of Tropical Insect Science. 1986;7:511-3. | Nigeria |
| Aeschlimann A, & Morel PC. *Boophilus geigyi* n. sp.(Acarina: Ixodoidea) une nouvelle tique du bétail de l’Ouest Africain. Acta Tropica. 1965;22:162-8. | Senegal |
| Agbede RI. A survey of ectoparasites and ectoparasitic conditions of animals in Zaria, Nigeria. Journal of Animal Production & Research. 1981;1:179-80. | Nigeria |
| Agbolade OM, Soetan EO, Awesu A, Ojo JA, Somoye OJ, & Raufu ST. Ectoparasites of domestic dogs in some Ijebu communities, Southwest Nigeria. World Applied Sciences Journal. 2008;3:916-20. | Nigeria |
| Agu NG, Okoye IC, Nwosu CG, Onyema I, Iheagwam CN, & Anunobi TJ. Prevalence of ectoparasites infestation among companion animals in Nsukka Cultural Zone. Annals of Medical & Health Sciences Research. 2020;10:1050-7. | Nigeria |
| Ahmed A, & George BD. Incidence of hard ticks (Ixodidae) on horses around Zaria, Nigeria. Nigerian Veterinary Journal. 2002;23:70-4. | Nigeria |
| Ait Lbacha H, Zouagui Z, Alali S, Rhalem A, Petit E, Ducrotoy MJ, et al. “Candidatus anaplasma camelii” in one-humped camels (*Camelus dromedarius*) in Morocco: a novel and emerging *Anaplasma* species?. Infectious Diseases of Poverty. 2017;6:1-8. | Morocco |
| Ajuwape AT, Sonibare AO, Adedokun RA, Adedokun OA, Adejinmi JO, & Akinboye DG. Infestation of royal python (Python regius) with ticks *Amblyomma hebraeum* in Ibadan Zoo, Nigeria. Tropical Veterinarian. 2003;21:38-41. | Nigeria |
| Akande FA, Adebowale AF, Idowu OA, & Sofela OO. Prevalence of ticks on indigenous breed of hunting dogs in Ogun State, Nigeria. Sokoto Journal of Veterinary Sciences. 2018;16:66-71. | Nigeria |
| Akinboade OA, & Dipeolu OO. Detection of *Babesia bovis* infections in *Boophilus geigyi* with egg crushings, larval smears, and haemolymph puncture. The Veterinary Quarterly. 1981;3:143-7. | Nigeria |
| Akinboade OA, & Dipeolu OO. Bovine babesiosis in Nigeria: Detection of *Babesia* organisms in salivary glands of *Boophilus decoloratus* collected on trade cattle. Zentralblatt für Veterinärmedizin Reihe B. 1983;30:153-5. | Nigeria |
| Alayande MO, Mayaki AM, Lawal MD, Abubakat A, Kassu M, & Talabi AO. Pattern of ticks and lice infestation on small ruminants in Sokoto, Sokoto State. Nigerian Journal of Animal Science. 2016;18:183-9. | Nigeria |
| Alayande MO, Mayaki AM, Lawal MD, Bandi NI, Ibrahim DD, & Talabi AO. Pattern of tick infestation on one humped camels (*Camelus dromedarius*) in Sokoto, Nigeria. Bulletin of Animal Health & Production in Africa. 2015;63:349-54. | Nigeria |
| Alimi D, Hajri A, Jallouli S, &  Sebai H. In vitro acaricidal activity of essential oil and crude extracts of *Laurus nobilis*, (Lauraceae) grown in Tunisia, against arthropod ectoparasites of livestock and poultry: *Hyalomma scupense* and *Dermanyssus gallinae*. Veterinary Parasitology. 2021; 298:109507. | Tunisia |
| Alimi D, Hajri A, Jallouli S, & Sebai H. Phytochemistry, anti-tick, repellency and anti-cholinesterase activities of *Cupressus sempervirens L.* and *Mentha pulegium L.* combinations against *Hyalomma scupense* (Acari: Ixodidae). Veterinary Parasitology. 2022;303:109665. | Tunisia |
| Ameen SA, Odetokun IA, Ghali-Muhammed LI, Azeez OM, Raji LO, Kolapo TU, et al. Status of ticks infestation in ruminant animals in Ogbomoso area of Oyo State, Nigeria. Journal of Environmental Issues and Agriculture in Developing Countries. 2014;6:48-53. | Nigeria |
| Ameh IG. A description of some ectoparasites of the wall gecko. Journal of Entomology. 2005;2:21-4. | Nigeria |
| Amoo AO, Dipeolu OO, Akinboade AO, & Adeyemi A. Bacterial isolation from and transmission by *Boophilus decoloratus* and *Boophilus geigyi*. Folia Parasitologica. 1987;34:69-74. | Nigeria |
| Amuta E, Atu B, Houmsou R, & Ayashar J. Prevalence of *Rhipicephalus sanguineus* infestation and Babesia canis infection in dogs with respect to breed type and degree of freedom in Makurdi, Benue State, Nigeria. The Internet Journal of Parasitic Diseases. 2010;4:247-9. | Nigeria |
| Amuta EU, Houmsou RS, & Ogabiela M. Tick infestation of dogs in Makurdi metropolis, Benue State-Nigeria. The Internet Journal of Veterinary Medicine. 2010;7:15. | Nigeria |
| Anifowose OI, Takeet MI, Talabi AO, & Otesile EB. Molecular detection of *Ehrlichia ruminantium* in engorged *Amblyomma variegatum* and cattle in Ogun State, Nigeria. Journal of Parasitic Diseases. 2020;44:403-10. | Nigeria |
| Anyaegbunam LC, Obi ZC, & Ezeoke CM. Ectoparasitosis and endoparasites in local goats (*Capra hircus*) in Onitsha, Anambra State, Nigeria. International Journal of Fauna and Biological Studies. 2013;1:1-3. | Nigeria |
| Aouragh H, Chaibi R & Bachir AS. Infestation modalities of *Hyalomma aegyptium* (Acari, Oxydidae) on the spur-thighed tortoise *Testudo graeca* in semiarid areas of Algeria. Vie et Milieu. 2020;70:99-105. | Algeria |
| Aquino LC, Kamani J, Haruna AM, Paludo GR, Hicks CA, Helps CR, et al. Analysis of risk factors and prevalence of *haemoplasma* infection in dogs. Veterinary Parasitology. 2016;221:111-7. | Nigeria |
| Arong GA, Adetunji BA, Mowang DA, & Odu AE. Comparative distribution of ticks on dogs in the Calabar Metropolis, South-South Nigeria. European Journal of Zoological Research. 2013;2:14-8. | Nigeria |
| Arong GA, Okon OE, Obhiokhenan AA, Esekhagbe OR, Okorafor KA, & Emevatha O. The infestation rates and predilection sites of ticks on cattles and dogs in Calabar, Nigeria. International Journal of Current Research. 2012;4,73-76. | Nigeria |
| Arong GA, Shitta KB, James-Rugu NN, & Effanga EO. Seasonal variation in the abundance and distribution of Ixodid ticks on Mongrel, alsatian and mixed breeds of dogs (*Canis familiaris)* in Jos, in plateau state, North-central Nigeria. World Journal of Science and Technology. 2011;1:24-9. | Nigeria |
| Natala AJ, Balogun EO, Balogun JA, Inuwa HM, Nok AJ, Shiba T, et al. Identification and characterization of sialidase-like activity in the developmental stages of *amblyomma variagatum.* Journal of Medical Entomology. 2013;50:85-93. | Nigeria |
| Ayeni JS, Dipeolu OO, & Okaeme AN. Parasitic infections of the grey-breasted helmet guinea-fowl (*Numida meleagris galeata*) in Nigeria. Veterinary Parasitology. 1983;12:59-63. | Nigeria |
| Bakkes DK, Chitimia-Dobler L, Matloa D, Oosthuysen M, Mumcuoglu KY, Mans BJ, et al. Integrative taxonomy and species delimitation of *Rhipicephalus turanicus* (Acari: Ixodida: Ixodidae). International Journal for Parasitology. 2020;50:577-94. | Senegal |
| Baltazard M, Bahmanyar M, & Mopidi G. *Ornithodorus erraticus* et fièvres récurrentes. Bulletin de la Societe de Pathologie Exotique. 1950;43:595-601. | Senegal |
| Bayer W, & Maina JA. Seasonal pattern of tick load in Bunaji cattle in the subhumid zone of Nigeria. Veterinary Parasitology. 1984;15:301-7. | Nigeria |
| Baylet R, Gilbert-Desvallons Y, Fichez, Berton, & Vaillant. Syndromes pseudo-grippaux à Dakar fièvre Q. Bulletin de la Société de pathologie exotique et de ses filiales. 1958;66:289-95. | Senegal |
| Beati L, Meskini M, Thiers B, & Raoult D. *Rickettsia aeschlimannii* sp. nov., a new spotted fever group rickettsia associated with *Hyalomma marginatum* ticks. International Journal of Systematic & Evolutionary Microbiology. 1997;47:548-54. | Morocco |
| Belabed AI, Zediri H, Shehab A & Bouslama Z. The effect of altitude on seasonal dynamics of ticks (Acari: Ixodida) in Northeastern Algeria. Advances in Environmental Biology. 2015;9:169-84. | Algeria |
| Belkahia H, Said MB, El Hamdi S, Yahiaoui M, Gharbi M, Daaloul-Jedidi M, et al. First molecular identification and genetic characterization of *Anaplasma ovis* in sheep from Tunisia. Small Ruminant Research. 2014;121:404-10. | Tunisia |
| Benchikh-Elfegoun MC, Benakhla A, Bentounsi B, Bouattour A & Piarroux R. Identification et cinétique saisonnière des tiques parasites des bovins dans la région de Taher (Jijel) Algérie. Annales de médecine vétérinaire. 2007;151:209-14. | Algeria |
| Bendjeddou ML, Bouslama Z, Amr ZS & BaniHani R. Infestation and seasonal activity of *Ixodes vespertilionis* Koch, 1844 (Acari: Ixodidae) on the Maghreb mouse-eared bat, *Myotis punicus* Felten, 1977, in Northeastern Algeria. Journal of Vector Ecology. 2016;41:110-3. | Algeria |
| Benredjem W, Leulmi H, Bitam I, Raoult D & Parola P. *Borrelia garinii* and *Rickettsia monacensis* in *Ixodes ricinus* ticks, Algeria. Emerging Infectious Diseases. 2014;20:1776-7. | Algeria |
| Bensaoud C, Abdelkafi-Koubaa Z, Ben Mabrouk H, Morjen M, Hmila I, Rhim A, et al. *Hyalomma dromedarii* (Acari: Ixodidae) salivary gland extract inhibits angiogenesis and exhibits in vitro antitumor effects. Journal of Medical Entomology. 2017;54:1476-82. | Tunisia |
| Benyahia H, Diarra AZ, Gherissi DE, Bérenger JM, Benakhla A & Parola P. Molecular and MALDI-TOF MS characterization of *Hyalomma aegyptium* ticks collected from turtles and their associated microorganisms in Algeria. Ticks & Tick Borne Diseases. 2022;13:101858. | Algeria |
| Benyedem H, Lekired A, Mhadhbi M, Dhibi M, Romdhane R, Chaari S, et al. First insights into the microbiome of Tunisian *Hyalomma* ticks gained through next-generation sequencing with a special focus on *H. scupense*. PLoS One. 2022;17:e0268172. | Tunisia |
| Bida SA, & Adams EW. Heartwater in a Bunaji calf: a case report. Veterinary Record. 1973;92:200-1. | Nigeria |
| Bitam I, Kernif T, Harrat Z, Parola P & Raoult D. First detection of *Rickettsia aeschlimannii* in *Hyalomma aegyptium* from Algeria. Clinical Microbiology & Infection. 2009;15:253-4. | Algeria |
| Bitam I, Parola P, Matsumoto K, Rolain JM, Baziz B, Boubidi SC, et al. First molecular detection of *R. conorii*, *R. aeschlimannii*, and *R. massiliae* in ticks from Algeria. Annals of the New York Academy of Sciences. 2006;1078:368-72. | Algeria |
| Biu AA & Konto M. Survey of tick species infesting the one humped camel C*amelus dromedarius* in Borno state, Nigeria. Journal of Agriculture & Veterinary Sciences. 2011;4:1-6. | Nigeria |
| Black F, Eley SM, Nuttall PA, & Moore NF. Characterisation of orbiviruses of the Kemerovo serogroup: comparison of protein and RNA profiles. Acta Virologica. 1986;30:320-4. | Morocco |
| Bouattour A, Darghouth MA, & Miled LB. Cattle infestation by *Hyalomma* ticks and prevalence of *Theileria* in *H. detritum* species in Tunisia. Veterinary Parasitology. 1996;65:233-45. | Tunisia |
| Bouchama B, Dik B, Benia F & Mouffok C. Dynamique saisonnière des tiques (Acari: Ixodidae) parasites des bovins dans la région semi-aride de la wilaya de Sétif Algérie. Bulletin de la Société Zoologique de France. 2020;145:71-81. | Algeria |
| Boudebouch N, Sarih M, Socolovschi C, Amarouch H, Hassar M, Raoult D, et al. Molecular survey for spotted fever group rickettsiae in ticks from Morocco. Clinical Microbiology & Infection. 2009;15:259-60. | Morocco |
| Bouhous A, Aissi M & Harhoura K. Prevalence of Ixodidae in sheep brought for slaughter in Adrar municipal abattoir, Southwest Algeria. Scientia Parasitologica. 2011;12:197-201. | Algeria |
| Boularias G, Azzag N, Galon C, Šimo L, Boulouis HJ & Moutailler S. High-throughput microfluidic real-time PCR for the detection of multiple microorganisms in Ixodid cattle ticks in Northeast Algeria. Pathogens. 2021;10:362. | Algeria |
| Boularias G, Azzag N, Gandoin C, Bouillin C, Chomel B, Haddad N, et al. *Bartonella bovis* and *Bartonella chomelii* infection in dairy cattle and their ectoparasites in Algeria. Comparative Immunology, Microbiology & Infectious Diseases. 2020;70:101450. | Algeria |
| Boulkaboul A. Parasitisme des tiques (Ixodidae) des bovins à Tiaret, Algérie. Revue d’ élevage et de médecine vétérinaire des pays tropicaux. 2003;56:157-62. | Algeria |
| Bouslama Z, Soualah-Alila H, Belabed A & Ouali K. Etude du système Tiques-lézard dans le parc national d’El Kala (Nord-Est algérie). Mésogée. 2009;65:73-83. | Algeria |
| Brès P, Cornet M, & Robin Y. Le virus de la forét de bandia (IPD-A 611), nouveau prototype d'arbovirus isolé au Sénégal.. Annales de l’Institut Pasteur. 1967;113:739-47. | Senegal |
| Bunza MD, Yahaya MM, Muhammad AS, Saidu AR. A survey on tick species infesting domestic birds sold at Sokoto central market, Nigeria. Sokoto Journal of Veterinary Sciences. 2008;7:52-54. | Nigeria |
| Buysse M, & Duron O. Two novel *Rickettsia* species of soft ticks in North Africa:‘Candidatus Rickettsia africaseptentrionalis’ and ‘Candidatus Rickettsia mauretanica’. Ticks & Tick-Borne Diseases. 2020;11:101376. | Morocco |
| Cafiso A, Bazzocchi C, De Marco L, Opara MN, Sassera D, & Plantard O. Molecular screening for *Midichloria* in hard and soft ticks reveals variable prevalence levels and bacterial loads in different tick species. Ticks & Tick-Borne Diseases. 2016;7:1186-92. | Nigeria |
| Camicas JL. *Argas* (Persicargas) *streptopelia* (Ixodoidea: Argasidae) on migrating and resident doves in Senegal. Annals of the Entomological Society of America. 1970;63:910. | Senegal |
| Camicas JL, Wilson ML, Cornet JP, Digoutte JP, Calvo MA, Adam F, et al. Ecology of ticks as potential vectors of Crimean-Congo hemorrhagic fever virus in Senegal: epidemiological implications. Hemorrhagic fever with renal syndrome, tick- and mosquito-borne viruses. Springer-Verlag. 1991;1:303-22. | Senegal |
| Causey OR, Kemp GE, Madbouly MH, David-West TS. Congo virus from domestic livestock, African hedgehog, and arthropods in Nigeria. American Journal of Tropical Medicine and Hygiene. 1970;19:846-50. | Nigeria |
| Chapman LE, Wilson ML, Hall DB, LeGuenno B, Dykstra EA, Ba K, et al. Risk factors for Crimean-Congo hemorrhagic fever in rural northern Senegal. Journal of Infectious Diseases. 1991;164:686-92. | Senegal |
| Chastel C, & Lay L. Pouvoir pathogène naturel pour l'homme d'un variant antigénique du virus Soldado isolé au Maroc. Bulletin de la Societe de Pathologie Exotique.1981;74;499-505 | Morocco |
| Chastel C, Bach-Hamba D, Karabatsos N, Bouattour A, Le Lay G, Le Goff F, et al. Tunis virus: a new Phlebovirus from *Argas reflexus hermanni* ticks in Tunisia. Acta Virologica. 1994;38:285-9. | Tunisia |
| Chastel C, Bailly-Choumara H, Bach-Hamba D, Le Lay G, Legrand MC, Le Goff F, et al. Tick-transmitted arbovirus in Maghreb. Bulletin de la Societe de Pathologie Exotique. 1995;88:81-5. | Algeria, Morocco |
| Chastel C, Main AJ, Bailly-Choumara H, Le Goff F, & Le Lay G. Essaouira and Kala iris: two new orbiviruses of the Kemerovo serogroup, Chenuda complex, isolated from *Ornithodoros* (Alectorobius) *maritimus* ticks in Morocco. Acta Virologica. 1993;37:484-92. | Morocco |
| Chiejina SN. Some parasitic diseases of intensively managed West African Dwarf sheep and goats in Nsukka, Eastern Nigeria. British Veterinary Journal. 1987;143:264-72. | Nigeria |
| Choudhury MK. Toxicity of neem seed oil against the larvae of *Boophilus decoloratus*, a one-host tick in cattle. Indian Journal of Pharmaceutical Sciences. 2009;71:562. | Nigeria |
| Cornet JP. Contribution à l'étude des tiques (Acarina: Ixodina) vectrices du virus de la Fièvre Hémorragique de Crimee-Congo (CCHF), au Sénégal. 3-*Rhipicephalus guilhoni* Morel et Vassilliades, variation de la taille en fonction de la charge parasitaire. Conséquences épidémiologiques. Acarologia. 1997;38:39-41. | Senegal |
| Cornet JP, Zeller H, & Camicas JL. Contribution à l'étude des tiques (Acarina: Ixodina) vectrices du virus de la fièvre hémorragique Crimée-Congo (CCHF) au Sénégal. II: Biologie, aux stases preimaginales, de *Hyalomma marginatum rufipes*. Acarologia. 1995;36:293-5. | Senegal |
| Cornet JP, Zeller H, Ba K, Camicas JL, Gonzalez JP, & Wilson ML. Contribution a l'études des tiques (Acarina: Ixodina) vectrices du virus de la fièvre hémorraggique Crimée-Congo (CCHF) au Sénégal. I: Analyse du parasitisme chez les petits rongeurs. Acarologia. 1995;36:287-92. | Senegal |
| Cutler SJ, Idris JM, Ahmed AO, Elelu N. *Ornithodoros savignyi,* the tick vector of “*Candidatus Borrelia kalaharica*” in Nigeria. Journal of Clinical Microbiology. 2018;56:e00532-18. | Nigeria |
| Dahmani M, Davoust B, Sambou M, Bassene H, Scandola P, Ameur T, et al. Molecular investigation and phylogeny of species of the Anaplasmataceae infecting animals and ticks in Senegal. Parasites & Vectors. 2019;12:1-5. | Senegal |
| Daodu OB, Eisenbarth A, Schulz A, Hartlaub J, Olopade JO, Oluwayelu DO, et al. Molecular detection of *Dugbe orthonairovirus* in cattle and their infesting ticks *Amblyomma and Rhipicephalus (Boophilus)* in Nigeria. PLoS Neglect Tropical Diseases. 2021;15:e0009905. | Nigeria |
| Davou KP, Dogo GA, Tanko J, Bialla M, Kogi CA. Epidemiology of Ectoparasites Infestation in Jos North, Plateau State, Nigeria. Saudi Journal of Medical and Pharmaceutical Sciences. 2017;3:206-10. | Nigeria |
| Deme GG, Malann YD, Olanrewaju CA, & Lumi EB. Ticks and tick-borne infections in some livestocks slaughtered at Gwagwalada Abattoir, federal capital territory, Abuja, Nigeria. Nigerian Journal of Parasitology. 2017;38:258-60. | Nigeria |
| Demoncheaux JP, Socolovschi C, Davoust B, Haddad S, Raoult D, & Parola P. First detection of *Rickettsia aeschlimannii* in *Hyalomma dromedarii* ticks from Tunisia. Ticks and Tick-Borne Diseases. 2012;3:398-402. | Tunisia |
| Agwunobi DO, Kamani J, Zheng H, Guo L, Yu Z, & Liu J. Bacterial diversity in *Rhipicephalus sanguineus* (Acari: Ixodidae) from two states in Nigeria. Journal of Entomology Science. 2021;56:256-71. | Nigeria |
| Diatta G, Mediannikov O, Boyer S, Sokhna C, Bassène H, Fenollar F, et al. An alternative strategy of preventive control of tick-borne relapsing fever in rural areas of Sine-Saloum, Senegal. The American Journal of Tropical Medicine and Hygiene. 2016;95:537. | Senegal |
| Diatta G, Duplantier JM, Granjon L, Ba K, Chauvancy G, Ndiaye M, et al. Borrelia infection in small mammals in West Africa and its relationship with tick occurrence inside burrows. Acta Tropica. 2015;152:131-40. | Senegal |
| Dib L, Bitam I, Bensouilah M, Parola P & Raoult D. First description of *Rickettsia monacensis* in *Ixodes ricinus* in Algeria. Clinical Microbiology & Infectection. 2009;15:261-2. | Algeria |
| Dib L, Lafri I, Boucheikhchoukh M, Dendani Z, Bitam I & Benakhla A. Seasonal distribution of *Rickettsia* spp. in ticks in northeast Algeria. New Microbes and New Infections. 2018;27:48-52. | Algeria |
| Dipeolu OO. Studies on ticks of veterinary importance in Nigeria. 1983. International Journal of Acarology. 1983;9:55-61. | Nigeria |
| Dipeolu OO. Studies on ticks of veterinary importance in Nigeria: XIV. Seasonal variation in the population of ticks on experimentally and naturally infested pastures in the forest zone of Nigeria. International Journal of Acarology. 1983;9:55-61. | Nigeria |
| Dipeolu OO. A survey of the ectoparasitic infestations of dogs in Nigeria. Journal of Small Animal Practice. 1975;16:123-9. | Nigeria |
| Dipeolu OO. Survey of tick infestation in the trade cattle and sheep and goats in Nigeria. Bulletin of Animal Health and Production in Africa. 1975;23:165-72. | Nigeria |
| Dipeolu OO. The incidence of ticks of *Boophilus* species on cattle, sheep, and goats in Nigeria. Tropical Animal Health and Production. 1975;7:35-9 | Nigeria |
| Dipeolu OO. The occurrence of ticks on a baby African elephant in Nigeria. African Journal of Ecology. 1976;14:227. | Nigeria |
| Dipeolu OO. Tick paralysis in a sheep caused by nymphs of *Amblyomma variegatum*: A preliminary report. Parasitology Research. 1976;49:293-5. | Nigeria |
| Dipeolu OO. Studies on ticks of veterinary importance in Nigeria. VII. Comparison of some aspects of the biology of *Boophilus decoloratus* and *Boophilus geigyi.* Tropical Veterinarian. 1984;2:22-32. | Nigeria |
| Dipeolu OO. Studies on ticks of veterinary importance in Nigeria. X. Notes on the biology of ticks of dogs *Rhipicephalus sanguineus* and *Haemaphysalis leachi leachi*. Bulletin of Animal Health and Production in Africa. 1984;32:1-15. | Nigeria |
| Dipeolu OO & Adeyefa CAO. Studies on ticks of veterinary importance in Nigeria. VIII. Differences observed in the biology of ticks which fed on different domestic animal hosts. Folia Parasitologica. 1984;31:53-61. | Nigeria |
| Dipeolu OO, Ajayi SS. Parasites of the African giant rat (*Cricetomys gambianus* Waterhouse) in Ibadan, Nigeria. Journal of East African Wildlife.1976;14:85-89. | Nigeria |
| Dipeolu OO, & Oduye OO. Survey of blood parasites of horses in Ibadan Western Nigeria. Annals of Parasitology. 1976;22:155-9. | Nigeria |
| Dipeolu OO, & Ogunji FO. The transmission of *Theileria annulata* to a rabbit by the larvae of the tick *Hyalomma rufipes*. Laboratory Animals. 1977;11:39-40. | Nigeria |
| Dipeolu OO, Akinboade OA, & Ogunji FO. Observations on the epidemiology of house infesting *Rhipicephalus sanguineus* in a household in Lagos, Nigeria. Bulletin of Animal Health and Production in Africa. 1982;30:29-30. | Nigeria |
| Dipeou OO, & Akinboade OA. Scavenging dogs and the spread of tick infestation in Nigeria. International Journal of Zoonoses. 1982;9:90-6. | Nigeria |
| Djerbouh A, Kernif T, Beneldjouzi A, Socolovschi C, Kechemir N, Parola P, et al. The first molecular detection of *Rickettsia aeschlimannii* in the ticks of camels from southern Algeria. Ticks & Tick Borne Diseases. 2012;3:374-6. | Algeria |
| Djerbouh A, Lafri I, Kechemir-Issad N, Bitam I. Endo-and ectoparasites (Ixodidae) of camels (*Camelus dromedarius*) from Southern Algeria. Livestock Research For Rural Development. 2018;30. | Algeria |
| Dsouli N, Younsi-Kabachii H, Postic D, Nouira S, Gern L, & Bouattour A. Reservoir role of lizard *Psammodromus algirus* in transmission cycle of *Borrelia burgdorferi* sensu lato (Spirochaetaceae) in Tunisia. Journal of Medical Entomology. 2006;43:737-42. | Tunisia |
| Duron O, Binetruy F, Noël V, Cremaschi J, McCoy KD, Arnathau C, et al. Evolutionary changes in symbiont community structure in ticks. Molecular ecology. 2017;26:2905-21. | Algeria, Morocco, Nigeria, Senegal, Tunisia |
| Duron O, Noël V, McCoy KD, Bonazzi M, Sidi-Boumedine K, Morel O, et al. The recent evolution of a maternally-inherited endosymbiont of ticks led to the emergence of the Q fever pathogen, *Coxiella burnetii*. PLoS Pathogens. 2015;11:e1004892. | Senegal |
| Edosomwan EU, Oke CO, Evbuomwan IO, & Imasuen AA. Prevalence of parasites of some wild birds in a tropical rainforest zone of Edo state, Nigeria. Nigerian Journal of Parasitology. 2018;39:259-60. | Nigeria |
| Ejima IA, & Ayegba AE. Relative abundance of hard tick on reared cattle (Family: Bovidae; Bos pp.) in Idah local government area (LGA), Kogi state, Nigeria. The Zoologist. 2011;9:9-16. | Nigeria |
| Ejima IA, Obayumi M, Olayemi IK, & Dangana MC. Tick infestations among cattle in Minna metropolis Niger state, Nigeria. Research Journal of Applied Sciences. 2014;9:126-32. | Nigeria |
| Ekanem MS, Mbagwu HO, Opara KN, & Agbata QC. Ticks infestation of domestic dogs (*Canis familiaris lupus*) in Uyo, Akwa Ibom state, Nigeria. World Journal of Applied Science and Technology. 2010;2:191-6. | Nigeria |
| Ekpenyong GD. The effect of climate on the seasonal activity and abundance of *Amblyomma variegatum* (Fabricius, 1794) (*Acarina: Ixodidae*) on trade cattle in Ibadan, Nigeria. Acarologia. 1996;37:165-72. | Nigeria |
| El Kammah KM, Hoogstraal H, & Camicas JL. Notes on African *Haemaphysalis* ticks: XI. H.(*Rhipistoma*) paraleachi (Ixodoidea: Ixodidae) distribution and hosts of adults. International Journal of Acarology. 1992;18:205-12. | Nigeria, Sierra Leone |
| El Mouden EH, Laghzaoui EM, Elbahi A, & Abbad A. A case of massive infestation of a female Spur-thighed tortoise *Testudo graeca* by blood-sucking ticks *Hyalomma aegyptium* (Acari: Ixodidae). Internation Journal of Acarology. 2020;46:63-5. | Morocco |
| Elati K, Bouaicha F, Dhibi M, Smida BB, Mhadhbi M, Obara I, et al. Phenology and phylogeny of *Hyalomma spp.* ticks infesting one-humped camels (*Camelus dromedarius*) in the Tunisian Saharan bioclimatic zone. Parasite. 2021;28:44. | Tunisia |
| Elbir H, FotsoFotso A, Diatta G, Trape JF, Arnathau C, Renaud F, et al. Ubiquitous bacteria *Borrelia crocidurae* in Western African ticks *Ornithodoros sonrai*. Parasites & Vectors. 2015;8:1-5. | Morocco, Senegal |
| Elelu N, Ola-Fadunsin SD, Bankole AA, Raji MA, Ogo NI, & Cutler SJ. Prevalence of tick infestation and molecular characterization of spotted fever *Rickettsia massiliae* in *Rhipicephalus* species parasitizing domestic small ruminants in North-central Nigeria. PLoS one. 2022;17:e0263843. | Nigeria |
| Elelu N, Bankole AA, Daphne HP, Rabiu M, Ola‐Fadunsin SD, Ambali HM, et al. Molecular characterisation of *Rhipicephalus sanguineus* sensu lato ticks from domestic dogs in Nigeria. Veterinary Medicine and Science. 2022;8:454-9. | Nigeria |
| Elhachimi L, Rogiers C, Casaert S, Fellahi S, Van Leeuwen T, Dermauw W, et al. Ticks and tick-borne pathogens abound in the cattle population of the Rabat-Sale Kenitra region, Morocco. Pathogens. 2021;10:1594. | Morocco |
| Elhamiani Khatat S, Daminet S, Kachani M, Leutenegger CM, Duchateau L, El Amri H, et al. *Anaplasma* spp. in dogs and owners in North-western Morocco. Parasites & Vectors. 2017;10:202. | Morocco |
| Elom MO, Nworie A, Ukwa BN, Uhuo CA, Nwele DE, & Ezeruigbo CF. Tick infestations and gastrointestinal helminthosis among goats and cattle at abattoirs in Abakaliki metropolis, Ebonyi state, Nigeria. Nigerian Journal of Parasitology. 2018;39:248-52. | Nigeria |
| Er-Rguibi O, Laghzaoui EM, Aglagane A, Kimdil L, Abbad A, & El Mouden EH. Determinants of prevalence and co-infestation by ecto-and endoparasites in the Atlas day gecko, *Quedenfeldtia trachyblepharus*, an endemic species of Morocco. Parasitology Research. 2021;120:2543-56. | Morocco |
| Estrada-Peña A, Nava S, & Petney T. Description of all the stages of *Ixodes inopinatus* n. sp.(Acari: Ixodidae). Ticks & Tick-Borne Diseases. 2014;5:734-43. | Algeria, Tunisia |
| Etim SE, Akpan PA & Okon VE. A survey of ectoparasites of dogs in Calabar. Nigerian Journal of Parasitology. 1996;17:153-155. | Nigeria |
| Eyo JE, Ekeh FN, Ivoke N, Atama CI, Onah IE, Ezenwaji NE, et al. Survey of tick infestation of cattle at four selected grazing sites in the tropics. Global Veterinaria. 2014;12:479-86. | Nigeria |
| Ezealor, AU. Parasites and diseases of Abdim's stork Ciconia abdimii. Malimbus. 1985;7:120. | Nigeria |
| Fabiyi JP. Arthropod parasites of domestic fowl and guineafowl on the Jos plateau, Northern Nigera. Tropical Animal Health and Production. 1980;12:193-4. | Nigeria |
| Fabiyi JP, Alayande MO, Lawal MD, Mahmuda A, & Usman M. Prevalence of ectoparasites attacking helmet guineafowl, *Numidamele agridis*, in Sokoto, North-western Nigeria. Nigerian Journal of Parasitology. 2016;37:113-6. | Nigeria |
| Fabiyi JP, Alayande MO, Lawal MD, Mahmuda A, & Usman M. Prevalence and significance of ectoparasites other than lice attacking chickens in Sokoto, North-west Nigeria. Nigerian Journal of Parasitology. 2017;38:125-7. | Nigeria |
| Fares W, Dachraoui K, Cherni S, Barhoumi W, Slimane TB, Younsi H, et al. Tick-borne encephalitis virus in Ixodes ricinus (Acari: Ixodidae) ticks, Tunisia. Ticks & Tick-Borne Diseases. 2021;12:101606. | Tunisia |
| Fares W, Dachraoui K, Najjar C, Younsi H, Findlay-Wilson S, Petretto M, et al. Absence of Crimean-Congo haemorrhagic fever virus in the tick *Hyalomma aegyptium* parasitizing the spur-thighed tortoise (*Testudo graeca*) in Tunisia. Parasite. 2019;26:35. | Tunisia |
| Faye O, Cornet JP, Camicas JL, Fontenille D, & Gonzalez JP. Transmission expérimentale du virus de la fièvre hémorragique de Crimée-Congo: Place de trois espèces vectrices dans les cycles de maintenance et de transmission au Sénégal. Parasite. 1999;6:27-32. | Senegal |
| Findlay GM, & Archer GT. The Occurrence of Tick-borne typhus in West Africa. Transactions of the Royal Society of Tropical Medicine and Hygiene.1948;41:815-8. | Nigeria |
| Flach EJ, Ouhelli H, Waddington D, & Hasnaoui ME. Prevalence of *Theileria* in the tick *Hyalomma detritum detritum* in the Doukkala region, Morocco. Medical and Veterinary Entomology. 1993;7:343-50. | Morocco |
| Foley H, & Parrot L. On the Occurrence of *Ornithodorus marocanus* Velu in Algeria. Bulletin de la Société de Pathologie Exotique. 1929;22:436. | Algeria |
| Gemel R, & Hörweg C. Zum Befall der Maurischen Landschildkröte *Testudo graeca* Linnaeus, 1758 durch Zecken, und deren Bedeutung als Vektoren. Ein Literaturüberblick samt eigenen Beobachtungen (Testudines: Testudinidae). Herpetozoa. 2011;23:21-30. | Morocco |
| Ghamba PE, Goje LJ, Sheriff AB. Viral detection in ticks and the level of ticks’infestations amongst small ruminants in Maiduguri, Borno state. Bima Journal Of Science And Technology. 2022;6:125-34. | Nigeria |
| Gharbi M, Drissi G, & Darghouth MA. Population dynamics of ticks infesting horses in North-west Tunisia. Revue Scientifique et Technique (International Office of Epizootics). 2018;37:837-41. | Tunisia |
| Gharbi M, Hayouni ME, Sassi L, Dridi W, & Darghouth MA. *Hyalomma scupense* (Acari, Ixodidae) in Northeast Tunisia: seasonal population dynamics of nymphs and adults on field cattle. Parasite. 2013;20:12. | Tunisia |
| Gharbi M, Moussi N, Jedidi M, Mhadhbi M, Sassi L, & Darghouth MA. Population dynamics of ticks infesting the one-humped camel (*Camelus dromedarius*) in central Tunisia. Ticks & Tick-Borne Diseases. 2013;4:488-91. | Tunisia |
| Gharbi M, Rjeibi MR, Rouatbi M, Mabrouk M, Mhadhbi M, Amairia S, et al. Infestation of the spur-thighed tortoise (*Testudo graeca)* by *Hyalomma aegyptium* in Tunisia. Ticks & Tick-Borne Disease. 2015;6:352-5. | Tunisia |
| Gharbi M, Sassi L, Dorchies P, & Darghouth MA. Infection of calves with *Theileria annulata* in Tunisia: Economic analysis and evaluation of the potential benefit of vaccination. Veterinary Parasitology. 2006;137:231-41. | Tunisia |
| Grard G, Lemasson JJ, Sylla M, Dubot A, Cook S, Molez JF, et al. Ngoye virus: a novel evolutionary lineage within the genus *Flavivirus*. Journal of General Virology. 2006;87:3273-7. | Senegal |
| Gueye A, Mbengue M, Dieye T, Diouf A, Seye M, & Seye MH. Cowdriosis in Senegal: some epidemiological aspects. Revue d’élevage et de médecine vétérinaire des pays tropicaux. 1993;46:217 | Senegal |
| Gueye A, Mbengue M & Diouf A. Tiques et hemoparasitoses du betail au Senegal. III. La zone nord-soudanienne. Revue d’élevage et de médecine vétérinaire des pays tropicaux. 1989:42;411-420. | Senegal |
| Gueye A, Mbengue M, & Diouf A. Tiques et hemoparasitoses du betail au Senegal. IV. La zone sud-soudanienne. Revue d’élevage et de médecine vétérinaire des pays tropicaux. 1989;42:517-28. | Senegal |
| Gueye A, Mbengue MB, Diouf A. Tiques et hémoparasitoses du bétail au Sénégal. VI. La zone soudano-sahélienne. Revue d’élevage et de médecine vétérinaire des pays tropicaux. 1994;47:39-46. | Senegal |
| Gueye A, Mbengue M, Diouf A, Sonko ML. Tiques et hémoparasitoses du bétail au Sénégal. V. La zone nord-guinéenne. Revue d’élevage et de médecine vétérinaire des pays tropicaux. 1993;46:551-61. | Senegal |
| Gueye A, Mbengue M, Diouf A, Seye M. Tiques et hemoparasitoses du betail au Senegal. I. La region des Niayes. Revue d’élevage et de médecine vétérinaire des pays tropicaux. 1986;39:381-93. | Senegal |
| Gueye A, Mbengue M, Diouf A, Vassiliades G. Prophylaxie de la cowdriose et observations sur la pathologie ovine dans la région des Niayes au Sénégal. Revue d’élevage et de médecine vétérinaire des pays tropicaux. 1989;42:497-503. | Senegal |
| Harris DJ, Graciá E, Jorge F, Maia JP, Perera A, Carretero MA, et al. Molecular detection of Hemolivia (Apicomplexa: Haemogregarinidae) from ticks of North African *Testudo graeca* (Testudines: Testudinidae) and an estimation of their phylogenetic relationships using 18S rRNA sequences. Comparative Parasitology. 2013;80:292-6. | Algeria, Morocco |
| Heylen D, Day M, Schunack B, Fourie J, Labuschange M, Johnson S, et al. A community approach of pathogens and their arthropod vectors (ticks and fleas) in dogs of African Sub-Sahara. Parasites & Vectors. 2021;14:1-20. | Nigeria |
| Hoffmann G, & Lindau M. Zecken an Nutz‐und Wildtieren in Niger. Zeitschrift für Angewandte Entomologie. 1971;69:72-82. | Niger |
| Hoogstraal H, & El Kammah KM. Notes on African *Haemaphysalis* ticks. XH (*Kaiseriana*) aciculifer Warburton and H.(K.) rugosa Santos Dias, the African representatives of the spinigera subgroup (*Ixodoidea: Ixodidae*). Journal of Parasitology. 1972;58:960-78. | Senegal |
| Hornok S, Kontschán J, Takács N, Chaber AL, Halajian A, Abichu G, et al. Molecular phylogeny of *Amblyomma exornatum* and *Amblyomma transversale*, with reinstatement of the genus *Africaniella* (*Acari: Ixodidae*) for the latter. Ticks & Tick-Borne Diseases. 2020;11:101494. | Nigeria |
| Hornok S, Sándor AD, Tomanović S, Beck R, D'Amico G, Kontschán J, et al. East and west separation of *Rhipicephalus sanguineus* mitochondrial lineages in the Mediterranean Basin. Parasites & Vectors. 2017;10:39. | Algeria |
| Idoko IS, Edeh RE, Adamu AM, Machunga-Mambula S, Okubanjo OO, Balogun EO, et al. Molecular and serological detection of piroplasms in horses from Nigeria. Pathogens. 2021;10:508. | Nigeria |
| Idris HS, & Umar H. Prevalence of ectoparasites in goats (*Capra aegagrus hircus*) brought for slaughter in the Gwagwalada area, Abuja, Nigeria. Entomological Research. 2007;37:25-8. | Nigeria |
| Ikeme MM. *Haemaphysalis hoodi hoodi* (Warburton and Nuttall, 1909) on domestic chickens in Eastern Nigeria. Veterinary Record. 1972;90:33. | Nigeria |
| Ikeme MM. The diagnostic characteristics and host distribution of the larvae of some economically important ticks in Nigeria: The larvae of *Amblyomma variegatum* and *Boophilus decoloratus* of cattle. The Nigerian Journal of Entomology. 1976;1-2:69-81. | Nigeria |
| Ikpeze OO, Eneanya CI, & Onyido AE. Abundance of ticks in pasture at Nnamdi Azikiwe University, Awka. Advances in Bioresearch. 2016;7:124-31. | Nigeria |
| Ikpeze OO. Distribution of parasitic ticks (*Acarina: Ixodidae*) on cattle at designated cattle markets in Enugu, Ugwuoba and Amansea, South-eastern Nigeria. International Journal of Biological Science. 2010;2:51-5. | Nigeria |
| Ikpeze OO, Eneanya CI, Chinweoke OJ, Aribodor DN, & Anyasodor AE. Species diversity, distribution and predilection sites of ticks (*Acarina: Ixodidae*) on trade cattle at Enugu and Anambra states, South-eastern Nigeria. The Zoologist. 2011;9:1-8. | Nigeria |
| Iwuala MO, & Okpala I. Studies on the ectoparasitic fauna of Nigerian livestock I: Types and distribution patterns on hosts'. Bulletin of Animal Health and Production in Africa. 1978;26:339-50. | Nigeria |
| Iwuala MO, & Okpala I. Studies on the ectoparasitic fauna of Nigerian livestock II: Seasonal infestation rates. Bulletin of Animal Health and Production in Africa. 1978;26:351-9. | Nigeria |
| Ja'afaru AI, Garba HS, Uko OJ, Lawal MD, Habibullah SA, Shehu SA, et al. Severe ectoparasitism and parasitic gastroenteritis in a two month old Sokoto red kid: a case report. Sokoto Journal of Veterinary Sciences. 2008;7:30-2. | Nigeria |
| James NN & Iwuala MOE. Seasonal abundance of Ixodid ticks from some livestock in Plateau state. Technology and Development. 1992;2:61-67. | Nigeria |
| James-Rugu NN & Iwuala MO. Ectoparasites of some domestic animals on the Jos plateau. Nigeria Science Forum. 2002;5:146-156. | Nigeria |
| James-Rugu NN & Iwuala MO. Ticks of Nigerian livestock with different fur conditions and colour shades. African Journal of Natural Sciences. 2002;3:102-6. | Nigeria |
| James-Rugu NN & Jidayi S. A survey on the ectoparasites of some livestock from some areas of Borno and Yobe states. Nigerian Veterinary Journal. 2004;25:48-55. | Nigeria |
| Johnston JE. A summary of an entomological survey of Kaduna district, Northern Nigeria. Bulletin of Entomological Research. 1916;7:19-28. | Nigeria |
| Unsworth K. The Ixodid parasites of cattle in Nigeria, with particular reference to the Northern territories. Annals of Tropical Medicine & Parasitology. 1952;46:331-6. | Nigeria |
| Kaaboub EA, Ouchene N, Ouchene NA, Dahmani A, Ouchtati I, Haif A, et al. Investigation of the principal vectors of abortive diseases in one-humped camels (*Camelus dromedarius*). Iraqi Journal of Veterinary Sciences. 2021;35:411-5. | Algeria |
| Kamani J, Baneth G, Mumcuoglu KY, Waziri NE, Eyal O, Guthmann Y, et al. Molecular detection and characterization of tick-borne pathogens in dogs and ticks from Nigeria. PLoS Neglected Tropical Diseases. 2013;7:e2108. | Nigeria |
| Kamani J, Morick D, Mumcuoglu KY, & Harrus S. Prevalence and diversity of *Bartonella* species in commensal rodents and ectoparasites from Nigeria, West Africa. PLoS Neglected Tropical Diseases. 2013;7:e2246. | Nigeria |
| Kamani J. Molecular evidence indicts *Haemaphysalis leachi* (*Acari: Ixodidae*) as the vector of *Babesia rossi* in dogs in Nigeria, West Africa. Ticks & Tick-Borne Diseases. 2021;12:101717. | Nigeria |
| Kamani J. Molecular identification and genetic analysis of *Rhipicephalus sanguineus* sensu lato of dogs in Nigeria, West Africa. Experimental and Applied Acarology. 2021;85:277-89. | Nigeria |
| Kamani J, Apanaskevich DA, Gutiérrez R, Nachum-Biala Y, Baneth G, & Harrus S. Morphological and molecular identification of *Rhipicephalus* (*Boophilus*) *microplus* in Nigeria, West Africa: a threat to livestock health. Experimental and Applied Acarology. 2017;73:283-96. | Nigeria |
| Kamani J, Baneth G, Apanaskevich DA, Mumcuoglu KY, & Harrus S. Molecular detection of *Rickettsia aeschlimannii* in *Hyalomma* spp. ticks from camels (*Camelus dromedarius*) in Nigeria, West Africa. Medical and Veterinary Entomology. 2015;2:205-9. | Nigeria |
| Kamani J, Baneth G, Gutiérrez R, Nachum-Biala Y, Mumcuoglu KY, & Harrus S. *Coxiella burnetii* and *Rickettsia conorii*: Two zoonotic pathogens in peridomestic rodents and their ectoparasites in Nigeria. Ticks & Tick-Borne Diseases. 2018;9:86-92. | Nigeria |
| Kamani J, Chung PJ, Lee CC, & Chung YT. In search of the vector (s) of *Babesia rossi* in Nigeria: Molecular detection of *B. rossi* DNA in *Rhipicephalus sanguineus* sensu lato (Acari: Ixodidae) ticks collected from dogs, circumstantial evidence worth exploring. Experimental and Applied Acarology. 2018;76:243-8. | Nigeria |
| Kamani J, González-Miguel J, Mshelbwala FM, Shekaro A, & Apanaskevich DA. Ticks (Acari: Ixodidae) infesting dogs in Nigeria: epidemiological and public health implications. Experimental and Applied Acarology. 2019;78:231-46. | Nigeria |
| Kamani J, Harrus S, Nachum-Biala Y, Gutiérrez R, Mumcuoglu KY, & Baneth G. Prevalence of *Hepatozoon* and *Sarcocystis* spp. in rodents and their ectoparasites in Nigeria. Acta Tropica. 2018;187:124-8. | Nigeria |
| Kamani J, Jwander LD, & Ubali Z. Demonstration of vermicules of *Babesia* species in haemolymph smears of *Amblyomma variegatum* in Nigeria. Journal of Advanced Veterinary Research. 2011;1:1-3. | Nigeria |
| Kamani J, Sannusi A, Dogo AG, Tanko JT, Egwu KO, Tafarki AE, et al. *Babesia canis* and *Babesia rossi* co-infection in an untraveled Nigerian dog. Veterinary Parasitology. 2010;173:334-5. | Nigeria |
| Kamara JA. Some parasites of wild animals in Sierra Leone. Bulletin of Animal Health and Production in Africa. 1973;23:265-268. | Sierra Leone |
| Kautman M, Tiar G, Papa A, & Široký P. AP92-like Crimean-Congo Hemorrhagic Fever Virus in *Hyalomma aegyptium* Ticks, Algeria. Emerging Infectious Diseases. 2016;22:354-6. | Algeria |
| Kebbi R, Nait-Mouloud M, Hassissen L, & Ayad A. Seasonal activity of ticks infesting domestic dogs in Bejaia province, Northern Algeria. Onderstepoort Journal of Veterinary Research. 2019;86:1-6. | Algeria |
| Keita AK, Mediannikov O, Ratmanov P, Diatta G, Bassene H, Roucher C, et al. Looking for Tropheryma whipplei source and reservoir in rural Senegal. The American Journal of Tropical Medicine and Hygiene. 2013;88:339-43. | Senegal |
| Kemp GE, Lee VH, & Moore DL. Isolation of Nyamanini and Quaranfil viruses from *Argas (*Persicargas*) arboreus* ticks in Nigeria. Journal of Medical Entomology. 1975;12:535-7 | Nigeria |
| Kernif T, Djerbouh A, Mediannikov O, Ayach B, Rolain JM, Raoult D, et al. *Rickettsia africae* in *Hyalomma dromedarii* ticks from sub-Saharan Algeria. Ticks & Tick-Borne Diseases. 2012;3:377-9. | Algeria |
| Kernif T, Messaoudene D, Ouahioune S, Parola P, Raoult D, & Bitam I. Spotted fever group rickettsiae identified in *Dermacentor marginatus* and *Ixodes ricinus* ticks in Algeria. Ticks & Tick-Borne Diseases. 2012;3:380-1. | Algeria |
| Khaldi M, Socolovschi C, Benyettou M, Barech G, Biche M, Kernif T, et al. Rickettsiae in arthropods collected from the North African hedgehog (*Atelerix algirus*) and the desert hedgehog (*Paraechinus aethiopicus)* in Algeria. Comparative Immunology, Microbiology and Infectious Diseases. 2012;35:117-22. | Algeria |
| Khallaayoune K, Biron JM, Chaoui A, & Duvallet G. Efficacy of 1% geraniol (Fulltec®) as a tick repellent. Parasite. 2009;16:223-6. | Morocco |
| Kheira LA, Radhwane SA, Nora MI, Farouk BE, Ratiba BA, Rachid CH, et al. The study of ectoparasites and mesoparasites in turtles (*Testudo Graeca Graeca*) in the region of Laghouat (South of Algeria). Bulletin of the University of Agricultural Sciences & Veterinary Medicine Cluj-Napoca. 2020;77:61-9. | Algeria |
| Khelifi-Ouchene NA, Ouchene N, Dahmani A, Kaaboub EA, Ouchetati I, & Haif A. Investigation of internal and external parasites of the camels (*Camelus dromedarius*) in Algeria. Annals of Parasitology. 2020;66:331-7. | Algeria |
| Khrouf F, M'Ghirbi Y, Znazen A, Ben Jemaa M, Hammami A, & Bouattour A. Detection of Rickettsia in *Rhipicephalus sanguineus* ticks and *Ctenocephalides felis* fleas from southeastern Tunisia by reverse line blot assay. Journal of Clinical Microbiology. 2014;52:268-74. | Tunisia |
| Kiouani A, Azzag N, Tennah S, & Ghalmi F. Infection with *Babesia canis* in dogs in the Algiers region: Parasitological and serological study. Veterinary World. 2020;13:1351-7. | Algeria |
| Konto M, Biu AA, Ahmed MI, and Charles S. Prevalence and seasonal abundance of ticks on dogs and the role of *Rhipicephalus sanguineus* in transmitting *Babesia* species in Maidugiri, North-Eastern Nigeria. Veterinary World. 2014;7:119-24. | Nigeria |
| Laamri M, El Kharrim K, Mrifag R, Boukbal M, & Belghyti D. Population dynamics of cattle parasitic ticks in the Gharb region of Morocco. Revue d’élevage et de Médecine Vétérinaire des Pays Tropicaux. 2012;65:57. | Morocco |
| Laatamna A, Oswald B, Chitimia-Dobler L, & Bakkes DK. Mitochondrial 16S rRNA gene analysis reveals occurrence of *Rhipicephalus sanguineus* sensu stricto from steppe and high plateaus regions, Algeria. Parasitology Research. 2020;119:2085-91. | Algeria |
| Lafri I, Benredjem W, Neffah-Baaziz F, Lalout R, Abdelouahed K, Gassen B, et al. Inventory and update on argasid ticks and associated pathogens in Algeria. New Microbes & New Infections. 2018;23:110-4. | Algeria |
| Lafri I, El Hamzaoui B, Bitam I, Leulmi H, Lalout R, Mediannikov O, et al. Detection of relapsing fever *Borrelia* spp., *Bartonella* spp. and *Anaplasmataceae* bacteria in argasid ticks in Algeria. PLoS Neglected Tropical Diseases. 2017;11:e0006064. | Algeria |
| Laghzaoui EM, Kasrati A, Abbad A, Leach D, Spooner-Hart R, & El Mouden EH. Acaricidal properties of essential oils from Moroccan plants against immature ticks of *Hyalomma aegyptium* (Linnaeus, 1758); an external parasite of the spur-thighed tortoise (Testudo graeca). International Journal of Acarology. 2018;44:315-21. | Morocco |
| Laghzaoui EM, Bouazza A, Abbad A, & El Mouden EH. Cross-sectional study of ticks in the vulnerable free-living spur-thighed tortoise *Testudo graeca* (Testudines: Testudinidae) from Morocco. International Journal of Acarology. 2022;48:76-83. | Morocco |
| Lakehal K, Saidi R, Rahmani MM, Kaidi R, Mimoune N, Benaceur F. Razlika u infestaciji krpeljima: *Hyalomma dromedarii Rhipicephalus sanguineus* sensu lato na jugu Alžira. Veterinarska Stanica. 2021;52:331-7. | Algeria |
| Lawal MD, Ameh IG, & Ahmed A. Some ectoparasites of *Camelus dromedarius* in Sokoto, Nigeria. Journal of Entomology. 2007;4:143-8. | Nigeria |
| Lawal MD, Mahmuda A, Fabiyi JP, George BD, Adamu Y, Kabir A, et al. A preliminary study on the monthly dynamics of cattle tick infestation in Sokoto, Northwestern Nigeria. Nigerian Journal of Animal Production. 2017;44:296-300. | Nigeria |
| Lee VH, Kemp GE, Madbouly MH, Moore DL, Causey OR, & Casals J. Jos, a new tick-borne virus from Nigeria. American Journal of Veterinary Research. 1974;35:1165-7. | Nigeria |
| Leeflang P, Pimentel WJ, Blotkamp J. The occurrence of *Haemobartonella canis* in Nigeria. Bulletin of Epizootic Diseases of Africa. 1974;22:51-3. | Nigeria |
| Lempereur L, Geysen D, & Madder M. Development and validation of a PCR–RFLP test to identify African *Rhipicephalus* (Boophilus) ticks. Acta Tropica. 2010;114:55-8. | Niger |
| Lo N, Beninati T, Sassera D, Bouman EA, Santagati S, Gern L, et al. Widespread distribution and high prevalence of an alpha-proteobacterial symbiont in the tick *Ixodes ricinus*. Environmental Microbiology. 2006;8:1280-7. | Algeria |
| Logan TM, Wilson ML, & Cornet JP. Association of ticks (*Acari: Ixodoidea*) with rodent burrows in northern Senegal. Journal of Medical Entomology. 1993;30:799-801. | Senegal |
| Lorusso V, Gruszka KA, Majekodunmi A, Igweh A, Welburn SC, & Picozzi K. *Rickettsia africae* in *Amblyomma variegatum* ticks, Uganda and Nigeria. Emerging Infectious Diseases. 2013;19:1705-7. | Nigeria |
| Lorusso V, Picozzi K, de Bronsvoort BM, Majekodunmi A, Dongkum C, Balak G, et al. Ixodid ticks of traditionally managed cattle in central Nigeria: where *Rhipicephalus* (Boophilus) *microplus* does not dare (yet?). Parasites & Vectors. 2013;6:171. | Nigeria |
| Lotfi D, & Karima K. Identification and incidence of hard tick species during summer season 2019 in Jijel Province (Northeastern Algeria). Journal of Parasitic Diseases. 2021;45:211-7. | Algeria |
| Madakan SP, Basu M, Mshelbara AJ, & Basu AK. Incidence of ticks (Acari: Ixodidae) or cattle in Maiduguri, Nigeria. Indian Veterinary Medicine Journal. 1998;17:63-5. | Nigeria |
| Maidala AM. A survey of cattle, sheep and goat ticks infestation in Katagum local government area of Bauchi state, Nigeria. International Journal of Agriculture & Earth Science. 2015;1:1-5. | Nigeria |
| Main AJ, Kloter KO, Camicas JL, Robin Y, and Sarr M. Wad medani and soldado viruses from ticks (*Ixodoidea*) in West Africa. Journal of Medical Entomology. 1980;17:380-2. | Senegal |
| Mamman AH, Lorusso V, Adam BM, Dogo GA, Bown KJ, & Birtles RJ. First report of *Theileria annulata* in Nigeria: Findings from cattle ticks in Zamfara and Sokoto states. Parasites & Vectors. 2021;14:1-9. | Nigeria |
| Mangombi JB, Roqueplo C, Sambou M, Dahmani M, Mediannikov O, Comtet L, et al. Seroprevalence of Crimean-Congo hemorrhagic fever in domesticated animals in northwestern Senegal. Vector-Borne and Zoonotic Diseases. 2020; 20:797–9. | Senegal |
| Matallah F, Benakhla A, Medjouel L, & Matallah S. Tick infestation of dogs and prevalence of canine babesiosis in the North-East of Algeria; area of El-Tarf. American-Eurasian Journal of Sustainable Agriculture. 2012;1:126-35. | Algeria |
| Mbaya AW, Mohammed AM, Okwudiri NC, Isa IU. Captive wild animals as potential reservoirs of haemo and ectoparasitic infections of man and domestic animals in the aridregion of Northeastern Nigeria. Veterinarski Arhiv. 2008;78:429-40. | Nigeria |
| Mediannikov O, Diatta G, Fenollar F, Sokhna C, Trape JF, & Raoult D. Tick-borne rickettsioses, neglected emerging diseases in rural Senegal. PLoS Neglected Tropical Diseases. 2010;4:e821. | Senegal |
| Mediannikov O, Diatta G, Kasongo K, & Raoult D. Identification of Bartonellae in the soft tick species *Ornithodoros sonrai* in Senegal. Vector-Borne and Zoonotic Diseases. 2014;14:26-32. | Senegal |
| Mediannikov O, Karkouri KE, Diatta G, Robert C, Fournier PE, & Raoult D. Non-contiguous finished genome sequence and description of *Bartonella senegalensis* sp. nov.. Standards in Genomic Sciences. 2013;8:279-89. | Senegal |
| Mediannikov O, Fenollar F, Socolovschi C, Diatta G, Bassene H, Molez JF, et al. *Coxiella burnetii* in humans and ticks in rural Senegal. PLoS Neglected Tropical Diseases. 2010;4:e654. | Senegal |
| Mediannikov O, Nguyen TT, Bell-Sakyi L, Padmanabhan R, Fournier PE, & Raoult D. High quality draft genome sequence and description of *Occidentia massiliensis* gen. nov., sp. nov., a new member of the family *Rickettsiaceae*. Standards in Genomic Sciences. 2014;9:1-8. | Senegal |
| Mediannikov O, Socolovschi C, Edouard S, Fenollar F, Mouffok N, Bassene H, et al. Common epidemiology of Rickettsia felis infection and malaria, Africa. Emerging Infectious Diseases. 2013;19:1775-83. | Senegal |
| Meeüs TD, Béati L, Delaye C, Aeschlimann A, & Renaud F. Sex‐biased genetic structure in the vector of Lyme disease, *Ixodes ricinus*. Evolution. 2002;56:1802-7. | Tunisia |
| Mohammed AN. Prevalence and experimental transmission of bovine piroplasms in Northern Nigeria. Bulletin of Animal Health and Production in Africa. 1976;24;171-80. | Nigeria |
| Mohammed AN. Seasonal incidence of Ixodid ticks of cattle in Northern Nigeria. Bulletin of Animal Health and Production in Africa. 1977;25:273-93. | Nigeria |
| Mohammed AN & Aliu YO. Nymphae of *Amblyomma variegatum*, adults of *Aponomma latum* and *Haemogregarina* spp. from the African beauty snake *Psammorphis sibilans*. Nigerian Journal of Science. 1973;7:17-8. | Nigeria |
| Mokhtaria K, Ammar AA, Mohammed Ammar SS, Chahrazed K, Fadela S, & Belkacem BT. Survey on species composition of Ixodidae tick infesting cattle in Tiaret (Algeria). Tropical Agriculture. 2018;95:102-5. | Algeria |
| Musa HI, Jajere SM, Adamu NB, Atsanda NN, Lawal JR, Adamu SG, et al. Prevalence of tick infestation in different breeds of cattle in Maiduguri, Northeastern Nigeria. Bangladesh Journal of Veterinary Medicine. 2014;12:161-6. | Nigeria |
| Myers BJ, Kuntz RE, & Kamara JA. Parasites and commensals of chimpanzees captured in Sierra-Leone, West-Africa. Proceedings of the Helminthological Society of Washington. 1973;40:298-9. | Sierra Leone |
| Natala AJ, Okubanjo OO, Ulayi BM, Owolabi YN, Jatau ID, & Yusuf KH. Ectoparasites of domestic animals in Northern Nigeria. Journal of Animal & Plant Sciences. 2009;3:238-42. | Nigeria |
| Ndiaye EH, Diouf FS, Ndiaye M, Bassene H, Raoult D, Sokhna C, et al. Tick-borne relapsing fever Borreliosis, a major public health problem overlooked in Senegal. PLoS Neglected Tropical Diseases. 2021;15:e0009184. | Senegal |
| Nduaka O, & Ikeme MM. Human skin lesions in East Central state, Nigeria due to the larvae of *Amblyomma variegatum* (Fabricius, 1794). Nigerian Medical Journal: Journal of the Nigeria Medical Association. 1973;3:140-3. | Nigeria |
| Ndumu PA, George JB, & Choudhury MK. Toxicity of neem seed oil (*Azadiracta indica*) against the larvae of *Amblyomma variegatum* a three‐host tick in cattle. Phytotherapy Research: An International Journal Devoted to Pharmacological and Toxicological Evaluation of Natural Product Derivatives. 1999;13:532-4. | Nigeria |
| Njila HL, Debi-Dore JD, Ombugadu A, Dibal M, & Mafuyai MJ. Survey of ectoparasites infesting captive birds in the Jos museum zoological garden, North Central, Nigeria. Journal of Natural Sciences Research. 2018;8:36-40. | Nigeria |
| Norte AC, Harris DJ, Silveira D, Nunes CS, Núncio MS, Martínez EG, et al. Diversity of microorganisms in *Hyalomma aegyptium* collected from spur‐thighed tortoise (*Testudo graeca*) in North Africa and Anatolia. Transboundary and Emerging Diseases. 2022;69:1951-62. | Algeria, Morocco, Tunisia |
| Nuttall GH, & Cuncliffe N. Notes on Ticks. III: On four new species of Ixodes. Parasitology. 1913;6:131-8. | Sierra Leone |
| Nwosu CO, Adamu M, Shinggu PA, & Ahmed MI. Parasites of camels (*Camelus dromedarius*) in Borno state, Nigeria. Nigerian Journal of Experimental and Applied Biology. 2003;4:65-70. | Nigeria |
| Obadiah HI, Onah IE, Ugochukwu JU, & Gbinde AK. Tick infestation of cattle in three markets in Makurdi, North-central, Nigeria. American Journal of Entomology. 2017;1:6-10. | Nigeria |
| Obadiah HI, & Shekaro A. Survey of tick infestation in cattle in Zaria abattoir, Nigeria. Journal of Veterinary Advances. 2012;2:81-7. | Nigeria |
| Obeta SS, Ibrahim B, Lawal IA, Natala JA, Ogo NI, & Balogun EO. Prevalence of canine babesiosis and their risk factors among asymptomatic dogs in the federal capital territory, Abuja, Nigeria. Parasite Epidemiology and Control. 2020;11:e00186. | Nigeria |
| Oduguwa BO, Oloyo OO, Talabi AD, Sogunle OA, Okwelum N, & Oloyo RA. Assessment of tick infestation and its effects on growth of extensively managed cattle in Ogun state, Nigeria. Nigerian Veterinary Journal. 2013;34:701-8. | Nigeria |
| Ofukwu RA, Ogbaje CI, & Akwuobu CA. Preliminary study of the epidemiology of ectoparasite infestation of goats and sheep in Makurdi, North Central Nigeria. Sokoto Journal of Veterinary Sciences. 2008;7:22-6. | Nigeria |
| Ogo N, de Mera IG, Okubanjo O, & de la Fuente J. Genetic characterization of *Coxiella burnetii* in *Amblyomma varigatum* ticks from North-central Nigeria: Public health importance. Veterinary World. 2013;6:818-22. | Nigeria |
| Ogo NI, de Mera IG, Galindo RC, Okubanjo OO, Inuwa HM, Agbede RI, et al. Molecular identification of tick-borne pathogens in Nigerian ticks. Veterinary Parasitology. 2012;187:572-7. | Nigeria |
| Ojeh CK & Dipeolu OO. The occurrence of *Aponoma ochraceum* (Acarina: Ixodidae) on a royal python in Ibadan, Nigeria. Nigerian Entomologists' Magazine.1983;4:36-7. | Nigeria |
| Okaeme AN. Ectoparasites and gastrointestinal parasites of nomadic cattle infiltrating into Kainji lake national park Nigeria. International Journal of Zoonoses. 1986;13:40-4. | Nigeria |
| Okaeme AN. Ectoparasites of guinea fowl (*Numida meleagris galeata* Pallas) and local domestic chicken (*Gallus gallus*) in Southern Guinea Savanna, Nigeria. Veterinary Research Communications. 1988;12:277-80. | Nigeria |
| Okaeme AN. Lameness associated with heavy ectoparasitic infestation in *Numidia meleagris galeata*, *Gallus domestica*, *Pavo multicus*. Bulletin of Animal Health and Production in Africa. 1989;37:189-90. | Nigeria |
| Okaeme AN, & Osakwe ME. Ectoparasites of the African hedgehog *Atelerix albiventris*,(Wagner) in the Kainji lake area of Nigeria. African Journal of Ecology. 1985;23:167-9. | Nigeria |
| Okeke JJ, Ikegbunam NM, Umeaniebue AC, Ezeonyejiaku DC, & Ezeadila JO. A survey on the ectoparasites and haemoparasites of grasscutter (*Thryonomys swinderianus*) reared under captive conditions. Journal of Natural Sciences Research. 2013;3:57-60. | Nigeria |
| Okewole E, & Adejinmi J. Comparison of two clinic-based immunoassays with the immunofluorescence antibody test for the field diagnosis of canine monocytic ehrlichiosis. Acta Microbiologica et Immunologica Hungarica. 2009;56:145-55. | Nigeria |
| Okiwelu SN, Ikpamii T, & Umeozor OC. Arthropods associated with mammalian carcasses in rivers state, Nigeria. African Journal of Biomedical Research. 2008;11:339-42. | Nigeria |
| Okoh AE, Oyetunde IL, & Ibu JO. Fatal heartwater in a captive Sitatunga. Veterinary Record. 1986;118:696. | Nigeria |
| Okoli IC, Okoli CG, & Opara M. Environmental and multi-host infestation of the brown dog tick, *Rhipicephalus sanguineus* in Owerri, South-east Nigeria- a case report. Veterinarski arhiv. 2006;76:93-100. | Nigeria |
| Okon EO, & Obiekazie AI. Parasites of cattle in Obudu cattle Ranch. Nigerian Veterinary Journal. 1981;10:1-4. | Nigeria |
| Okorafor KA, Odaibo AB, Eleng I, & Okete JA. Occurrence and prevalence of ecto and gastrointestinal parasites in wild cane rats (*Tryonomys swinderianus*) from Oyo state, South-Western Nigeria. European Journal of Zoological Research. 2012;1:70-6. | Nigeria |
| Okoye JO, & Ikeme MM. Acute dermatitis caused by *Amblyomma variegatum* larvae on chickens. Avian Pathology. 1990;19:785-9. | Nigeria |
| Okwuonu ES, Andong FA, & Ugwuanyi IK. Association of ticks with seasons, age, and cattle color of North-Western region of Nigeria. Scientific African. 2021;12:e00832. | Nigeria |
| Okwuonu ES, Bala AY, Ikpeze OO. Ixodid ticks infestation of zebu cattle crosses in Sokoto state Nigeria. The Bioscientist Journal. 2017;5:50-6. | Nigeria |
| Olabode HO, Silas PM, & Agbede RI. Survey of ectoparasites and their predilection sites on cattle in Bukuru Market. Journal of Agricultural and Veterinary Sciences. 2010;2:70-4. | Nigeria |
| Omonijo AO, & Sowemimo OA. Prevalence of ectoparasites of dogs and cats in Ijero and Moba LGAs, Ekiti State, Nigeria. Nigerian Journal of Parasitology. 2017;38:278-83. | Nigeria |
| Omonona A, Adeyanju T, & Eke F. Parasite Prevalence among wildbirds in two sites in Ibadan, South Western Nigeria. Production, Agriculture, and Technology Journal. 2014;10:65-77. | Nigeria |
| Omudu EA, & Amuta EU. Parasitology and urban livestock farming in Nigeria: prevalence of ova in faecal and soil samples and animal ectoparasites in Makurdi. Journal of the South African Veterinary Association. 2007;78:40-5. | Nigeria |
| Omudu EA, Iorlaha GT, & Adelusi S. Medically important arthropods infesting some exotic birds and mammals in the Makurdi zoological garden in Benue State, Nigeria. Scientific Journal of King Faisal University. 2011;12:239-49. | Nigeria |
| Onyali IO, Oluigbo FO, & Ajayi ST. Dry season outbreaks of *Ornithodoros savignyi* in Gashu'a, Borno State: a case report. Tropical Veterinarian. 1989;7:101-3. | Nigeria |
| Onyiche TE, Ogo NI, & Thekisoe O. Species distribution, prevalence, and risk factors associated with tick infestations of equines in Nigeria. International Journal of Acarology. 2022;48:201-6. | Nigeria |
| Onyiche TE, Taioe MO, Ogo NI, Sivakumar T, Biu AA, Mbaya AW, et al. Molecular evidence of *Babesia caballi* and *Theileria equi* in equines and ticks in Nigeria: prevalence and risk factors analysis. Parasitology. 2020;147:1238-48. | Nigeria |
| Onyiche TE, Răileanu C, Tauchmann O, Fischer S, Vasić A, Schäfer M, et al. Prevalence and molecular characterization of ticks and tick-borne pathogens of one-humped camels (*Camelus dromedarius*) in Nigeria. Parasites & Vectors. 2020;13:1-6. | Nigeria |
| Opara MN, & Ezeh NO. Ixodid ticks of cattle in Borno and Yobe states of Northeastern Nigeria: Breed and coat colour preference. Animal Research International. 2011;8:1359-65. | Nigeria |
| Opara MN, Abdu Y, Okoli IC. Survey of ticks of veterinary Importance and tick-borne protozoa of cattle grazed in very hot months in Sokoto Municipality, Nigeria. International Journal of Agriculture and Rural Development. 2005;6:168-74. | Nigeria |
| Opasina BA. Disease constraints on village goat production in Southwest Nigeria. Revue d’élevage et de médecine vétérinaire des pays tropicaux. 1985;38:284-94. | Nigeria |
| Opasina BA, Dipeolu OO, & Fagbemi BO. Some ectoparasites of veterinary importance on dwarf sheep and goats under traditional system of management in the humid forest and derived savanna zones of Nigeria. Revue d’élevage et de médecine vétérinaire des pays tropicaux. 1983;36:387-391. | Nigeria |
| Orhierhor M, Okaka CE, & Okonkwo VO. A survey of the parasites of the African white-bellied pangolin, *Phataginus tricuspis*, in Benin City, Edo State, Nigeria. Nigerian Journal of Parasitology. 2017;38:266-70 | Nigeria |
| Ouchene N, Nebbak A, Ouchene-Khelifi NA, Dahmani A, Zeroual F, Khelef D, et al. Molecular detection of avian spirochete *Borrelia anserina* in *Argas persicus* ticks in Algeria. Comparative Immunology, Microbiology and Infectious Diseases. 2020;68:101408. | Algeria |
| Ouhelli H, & Pandey VS. Prevalence of cattle ticks in Morocco. Tropical Animal Health and Production. 1982;14:151-4. | Morocco |
| Palomar AM, Portillo A, Santibáñez P, Mazuelas D, Arizaga J, Crespo A, et al. Crimean-Congo hemorrhagic fever virus in ticks from migratory birds, Morocco. Emerging Infectious Diseases. 2013;19:260. | Morocco |
| Pandey VS, Dakkak A, & Elmamoune M. Parasites of stray dogs in the Rabat region, Morocco. Annals of Tropical Medicine and Parasitology. 1987;81:53-5. | Morocco |
| Parent R, Alogninouwa T. Amélioration de la productivité de l'élevage en zone tropicale. Traitement systématique des vaches gestantes à l'Ivermectine dans les mois précédant la mise bas. Revue d’élevage et de médecine vétérinaire des pays tropicaux. 1984;37:341-54. | Senegal |
| Parola P, & Raoult D. Molecular tools in the epidemiology of tick-borne bacterial diseases. Annales de biologie clinique. 2001;59:77-82. | Niger |
| Parola P, Inokuma H, Camicas JL, Brouqui P, & Raoult D. Detection and identification of spotted fever group Rickettsiae and Ehrlichiae in African ticks. Emerging Infectious Diseases. 2001;7:1014-7. | Niger |
| Paul BT, Bello AM, Haruna NM, Dauda J, Ojo DT, & Gadzama MA. Infestation of zebu cattle (*Bos indicus linnaeus*) by hard ticks (acari: Ixodidae) in Maiduguri, Northeastern Nigeria. Persian Journal of Acarology. 2017;6:213-224. | Nigeria |
| Pearse AS. Ecology of the ectoparasites of Nigerian rodents and insectivores. Journal of Mammalogy. 1929;10:229-39. | Nigeria |
| Philip CB. Discovery in West Africa of *Hunterbllus hookeri howard,* parasite of ixodids. Annales de Parasitologie. 1931;9:276. | Nigeria |
| Philip CB. Occurrence of a colony of the tick parasite *Hunterellus hookeri* *howard* in West Africa. Public Health Reports. 1931;46:2168-72. | Nigeria |
| Pukuma SM, James-Rugu NN, & Sale M. A study on tick borne infections of cattle in Yola locality of Adamawa state. African Journal of Agricultural Research. 2011;6:6208-11. | Nigeria |
| Pullan NB. Productivity of white Fulani cattle on the Jos Plateau, Nigeria. III. Disease and management factors. Tropical Animal Health and Production. 1980;12:77-84. | Nigeria |
| Rahal M, Medkour H, Diarra AZ, Bitam I, Parola P, & Mediannikov O. Molecular identification and evaluation of Coxiella-like endosymbionts genetic diversity carried by cattle ticks in Algeria. Ticks & Tick-borne Diseases. 2020;11:101493. | Algeria |
| Randa ML, Meddour S, Bilal DI, Souttou K, & Sekour M. First report of ectoparasites from black rats (*Rattus rattus* Linnaeus, 1758) in oasis regions from Algeria. Notulae Scientia Biologicae. 2022;14:11013. | Algeria |
| René-Martellet M, Minard G, Massot R, Tran Van V, Valiente Moro C, Chabanne L, et al. Bacterial microbiota associated with *Rhipicephalus sanguineus* (sl) ticks from France, Senegal and Arizona. Parasites & Vectors. 2017;10:416. | Senegal |
| Reye AL, Arinola OG, Hübschen JM, & Muller CP. Pathogen prevalence in ticks collected from the vegetation and livestock in Nigeria. Applied & Environmental Microbiology. 2012;78:2562-8. | Nigeria |
| Rjeibi MR, Amairia S, Mhadhbi M, Rekik M, & Gharbi M. Detection and molecular identification of *Anaplasma phagocytophilum* and *Babesia* spp. infections in *Hyalomma aegyptium* ticks in Tunisia. Archives of Microbiology. 2022;204:385. | Tunisia |
| Rjeibi MR, Darghouth MA, Rekik M, Amor B, Sassi L, & Gharbi M. First molecular identification and genetic characterization of *Theileria lestoquardi* in sheep of the Maghreb region. Transboundary & Emerging Diseases. 2016;63:278-84. | Tunisia |
| Rjeibi MR, Gharbi M, Mhadhbi M, Mabrouk W, Ayari B, Nasfi I, et al. Prevalence of piroplasms in small ruminants in North-West Tunisia and the first genetic characterization of *Babesia ovis* in Africa. Parasite. 2014;21:23. | Tunisia |
| Robin Y, Camicas JL, Bres P, & Hery G. International symposium on tick-borne arboviruses: Observations on some viruses isolated from ticks in Senegal. Folia Parasitologica. 1970;17:345-8. | Senegal |
| Ros-García A, M'ghirbi Y, Bouattour A, & Hurtado A. First detection of *Babesia occultans* in *Hyalomma* ticks from Tunisia. Parasitology. 2011;138:578-82. | Tunisia |
| Sadeddine R, Diarra AZ, Laroche M, Mediannikov O, Righi S, Benakhla A, et al. Molecular identification of protozoal and bacterial organisms in domestic animals and their infesting ticks from North-eastern Algeria. Ticks & Tick-Borne Diseases. 2020;11:101330. | Algeria |
| Sadiq NA, Adejinmi JO, Adedokun OA, Fashanu SO, Alimi AA, & Sofunmade YT. Ectoparasites and haemoparasites of indigenous chicken (*Gallus domesticus*) in Ibadan and environs. Tropical Veterinarian. 2003;21:187-91. | Nigeria |
| Sadiq NA, Adejinmi JO, & Adedokun AO. Anthropophilic nature of the brown dog tick, *Rhipicephalus sanguineus* in Ibadan, Nigeria. Tropical Veterinarian. 2001;19:58-9. | Nigeria |
| Sahibi H, Rhalem A, Berrag B, & Goff WL. Bovine *Babesiosis*: Seroprevalence and ticks associated with cattle from two different regions of Morocco. Annals of the New York Academy of Sciences. 1998;849:213-8. | Morocco |
| Said MB, Belkahia H, Alberti A, Zobba R, Bousrih M, Yahiaoui M, et al. Molecular survey of Anaplasma species in small ruminants reveals the presence of novel strains closely related to *A. phagocytophilum* in Tunisia. Vector-Borne & Zoonotic Diseases. 2015;15:580-90. | Tunisia |
| Said MB, Galai Y, Canales M, Nijhof AM, Mhadhbi M, Jedidi M, et al. Hd86, the Bm86 tick protein ortholog in *Hyalomma scupense* (syn. *H. detritum*): expression in *Pichia pastoris* and analysis of nucleotides and amino acids sequences variations prior to vaccination trials. Veterinary Parasitology. 2012;183:215-23. | Tunisia |
| Said MB, Galai Y, Mhadhbi M, Jedidi M, de la Fuente J, & Darghouth MA. Molecular characterization of Bm86 gene orthologs from *Hyalomma excavatum*, *Hyalomma dromedarii* and *Hyalomma marginatum marginatum* and comparison with a vaccine candidate from *Hyalomma scupense*. Veterinary Parasitology. 2012;190:230-40. | Tunisia |
| Said Y, Lahmar S, Dhibi M, Rjeibi MR, Jdidi M, & Gharbi M. First survey of ticks, tick-borne pathogens (*Theileria*, *Babesia*, *Anaplasma* and *Ehrlichia*) and *Trypanosoma evansi* in protected areas for threatened wild ruminants in Tunisia. Parasitology International. 2021;81:102275. | Tunisia |
| Sa'idu L, Agbede RI, & Abdu AP. Prevalence of avian spirochaetosis in Zaria (1980-1989). Israel Journal of Veterinary Medicine. 1995;50:39-40. | Nigeria |
| Sakraoui F, Boukheroufa M, Sakraoui W, & El Madoui MB. Ectoparasitic ecology of Algerian hedgehog *Ateleris algirus* (Lereboullet, 1842)(Erinaceidae, Mammalia) in some localities of Edough Montain (W. Annaba, Northeast Algeria). Advances in Environmental Biology. 2014;1:217-22. | Algeria |
| Sambo SJ, Ibrahim ND, Esievo KA, Hambolu JO, Oladele SB, Sackey AK, et al. Co-existence of besnoitiosis and dermatophilosis in indigenous cattle slaughtered at Zaria abattoir. Journal of Animal and Veterinary Advances. 2007;6:617-620. | Nigeria |
| Sambou M, Faye N, Bassène H, Diatta G, Raoult D, & Mediannikov O. Identification of *rickettsial* pathogens in ixodid ticks in northern Senegal. Ticks & Tick-Borne Diseases. 2014;5:552-6. | Senegal |
| Sanusi M, Ahmed IA, Tahir I, Mai HM, Kalla DJ, & Shuaibu I. Survey of equine piroplasmosis in the savanna areas, Bauchi state, North-eastern Nigeria. Ippologia. 2014;25:3-8. | Nigeria |
| Sarih MH, M'Ghirbi Y, Bouattour A, Gern L, Baranton G, & Postic D. Detection and identification of *Ehrlichia* spp. in ticks collected in Tunisia and Morocco. Journal of Clinical Microbiology. 2005;43:1127-32. | Morocco |
| Sarih M, Socolovschi C, Boudebouch N, Hassar M, Raoult D, & Parola P. Spotted fever group rickettsiae in ticks, Morocco. Emerging Infectious Diseases. 2008;14:1067. | Morocco |
| Schein E, Rehbein G, Voigt WP, & Zweygarth E. *Babesia equi* (Laveran 1901). Development in horses and in lymphocyte culture. Tropical Medicine and Parasitology. 1981;32:223-7. | Morocco |
| Seddik MM, Cuaquil L, Driot C, & Khorchani T. Effects of herding methods on the kinetics of tick infestation of dromedary camel in southern Tunisia. Bulletin de la Société Zoologique de France. 2011;136;299-311. | Tunisia |
| Segura A, Rodríguez O, Ruiz-Fons F, & Acevedo P. Tick parasitism in the Mediterranean spur-thighed tortoise in the Maamora forest, Morocco. Ticks & Tick-Borne Diseases. 2019;10:286-9. | Morocco |
| Selmi R, Said MB, Mamlouk A, Yahia HB, & Messadi L. Molecular detection and genetic characterization of the potentially pathogenic *Coxiella burnetii* and the endosymbiotic Candidatus Midichloria mitochondrii in ticks infesting camels (*Camelus dromedarius*) from Tunisia. Microbial Pathogenesis. 2019;136:103655. | Tunisia |
| Senevet G, & Pampiglione S. Some species of Ixodidae from the Geryville region (high plateaus of Oran). Bulletin de la Societe de Pathologie Exotique et de ses Filiales. 1964;57:400-2. | Algeria |
| Seng P, Sarih M, Socolovschi C, Boudebouch N, Hassar M, Parola Pet al. Detection of Anaplasmataceae in ticks collected in Morocco. Clinical Microbiology and Infection. 2009;15:86-7. | Morocco |
| Sfar N, M'ghirbi Y, Letaief A, Parola P, Bouattour A, & Raoult D. First report of *Rickettsia monacensis* and *Rickettsia helvetica* from Tunisia. Annals of Tropical Medicine & Parasitology. 2008;102:561-4. | Tunisia |
| Shitta KB, James-Rugu NN, & Badaki JA. Prevalence of ticks on dogs in Jos, plateau state, Nigeria. Bayero Journal of Pure and Applied Sciences. 2018;11:451-4. | Nigeria |
| Simpson JJ. Entomological research in British West Africa. IV. Sierra Leone. Bulletin of Entomological Research. 1913;4:151-90. | Sierra Leone |
| Simpson JJ. Entomological research in British West Africa. II. Northern Nigeria. Bulletin of Entomological Research. 1914;2:301-356 | Nigeria |
| Simpson JJ. Entomological research in British West Africa. III. Southern Nigeria. Bulletin of Entomological Research, 1914;3:137-193. | Nigeria |
| Široký P, Mikulíček P, Jandzík D, Kami H, Mihalca AD, Rouag R, et al. Co-distribution pattern of a haemogregarine *Hemolivia mauritanica* (Apicomplexa: Haemogregarinidae) and its vector *Hyalomma aegyptium* (Metastigmata: Ixodidae). Journal of Parasitology. 2009;95:728-33. | Algeria, Morocco |
| Socolovschi C, Bitam I, Raoult D, & Parola P. Transmission of *Rickettsia conorii conorii* in naturally infected *Rhipicephalus sanguineus*. Clinical Microbiology & Infection. 2009;15:319-21. | Algeria |
| Soualah-Alila H, Bouslama Z, Amr Z, & Bani Hani R. Tick infestations (Acari: Ixodidae) on three lizard species from Seraidi (Annaba District), Northeastern Algeria. Experimental and Applied Acarology. 2015;67:159-63. | Algeria |
| Sylla M, & Thonnon J. *Argasidae (Acari: Ixodida)* parasites of wild and domestic animals in Senegal: 2-Arboviruses isolation and epidemiological implications. Acarologia. 2004;44:137-150. | Senegal |
| Sylla M, Molez JF, Cornet JP, & Camicas JL. Climate change and distribution of ticks (*Acari: Ixodida*) in Senegal and Mauritania. Acarologia. 2009;48:137-53. | Senegal |
| Sylla M, Molez JF, Cornet JP, Mondet B, Camicas JL. Les tiques (Acari: Ixodida) du Sénégal: Fréquence des hôtes répertoriés, dynamique saisonnière et chorologie d'*Amblyomma* (Xiphiastor) *variegatum* (Fabricius, 1794). Acarologia. 2006;47:13-23. | Senegal |
| Tager-Kagan P, Tibayrenc R, & Garba D. Epidemiology of poultry parasitic disease in village breeding in Niamey area, Niger. Revue d’élevage et de médecine vétérinaire des pays tropicaux. 1992;45:139-47. | Niger |
| Tags SZ, Agbede RI, & Mohammed BR. First incidence of ectoparasites in Abuja zoological parks, Abuja, Nigeria. Annals of Parasitology. 2020;66:533-7. | Nigeria |
| Takeet MI, Oyewusi IK, Ganiyu AI, Anifowose IO, Famuyide MI, Talabi OA, et al. Molecular detection of protozoan parasites in ticks infesting cattle entering Nigeria through a major trans-boundary route in Ogun state. Bulletin of Animal Health and Production in Africa. 2017;65:175-80. | Nigeria |
| Tiar G, Tiar‐Saadi M, Benyacoub S, Rouag R, Široký P. The dependence of *Hyalomma aegyptium* on its tortoise host *Testudo graeca* in Algeria. Medical & Veterinary Entomology. 2016;30:351-9. | Algeria |
| Tomlinson JA, & Apanaskevich DA. Two new species of *Haemaphysalis* Koch, 1844 (Acari: Ixodidae) in the *H.(Rhipistoma*) *spinulosa* subgroup, parasites of carnivores and hedgehogs in Africa. Systematic Parasitology. 2019;96:485-509. | Nigeria, Senegal |
| Tongjura JD, Amuga GA, Ombugadu RJ, Azamu Y, & Mafuiya HB. Ectoparasites infesting livestock in three local government areas (LGAs) of Nasarawa State, Nigeria. Science World Journal. 2012;7:15-7. | Nigeria |
| Trape JF, Godeluck B, Diatta G, Rogier C, Legros F, Albergel J et al. The spread of tick-borne borreliosis in West Africa and its relationship to sub-Saharan drought. The American Journal of Tropical Medicine and Hygiene. 1996;54:289-93. | Senegal |
| Trape JF, Diatta G, Arnathau C, Bitam I, Sarih MH, Belghyti D, et al. The epidemiology and geographic distribution of relapsing fever borreliosis in West and North Africa, with a review of the *Ornithodoros erraticus* complex (Acari: Ixodida). PLoS One. 2013;8:e7847. | Senegal |
| Ubah AS, Abalaka SE, Idoko IS, Obeta SS, Ejiofor CE, Mshelbwala PP, et al. Canine babesiosis in a male Boerboel: Hematobiochemical and anatomic pathological changes in the cardiorespiratory and reproductive organs. Veterinary and Animal Science. 2019;7:100049. | Nigeria |
| Ugbomoiko US, Ariza L, & Heukelbach J. Parasites of importance for human health in Nigerian dogs: high prevalence and limited knowledge of pet owners. BMC Veterinary Research. 2008;4:1-9. | Nigeria |
| Ugbomoiko US, & Obiamiwe BA. Distribution and incidence of ectoparasites on small mammals in a rainforest belt of Southern Nigeria. Angewandte Parasitologie. 1991;32:143-8. | Nigeria |
| Ugochukwu EI, & Apeh AO. Prevalence of ectoparasites of small ruminants in Nsukka, Nigeria. International Journal of Zoonoses. 1985;12:313-7. | Nigeria |
| Ugochukwu EI, & Nnadozie CC. Ectoparasitic infestation of dogs in Bendel State, Nigeria. International Journal of Zoonoses. 1985;12:308-12. | Nigeria |
| Ugochukwu EI, & Omije FA. Ectoparasitic fauna of poultry in Nsukka, Nigeria. International Journal of Zoonoses. 1986;13:93-7. | Nigeria |
| Umar YA, George BD,  & Ajanusi OJ. Survey of hard ticks (Ixodidae) infesting one-humped camels (*Camelus dromedarius)* in Kano State-Nigeria. Nigerian Journal of Parasitology. 2011;32:61-66. | Nigeria |
| Vial L, Diatta G, Tall A, Bouganali H, Durand P, Sokhna C, et al. Incidence of tick-borne relapsing fever in west Africa: longitudinal study. The Lancet. 2006;368:37-43. | Senegal |
| Vial L, Durand P, Arnathau C, Halos L, Diatta G, Trape JF, et al. Molecular divergences of the *Ornithodoros sonrai* soft tick species, a vector of human relapsing fever in West Africa. Microbes & Infection. 2006;8:2605-11. | Senegal |
| Vial L, Wieland B, Jori F, Etter E, Dixon L, & Roger F. African swine fever virus DNA in soft ticks, Senegal. Emerging Infectious Diseases. 2007;3:1928-31. | Senegal |
| Walton GA. The *Ornithodorus moubata* superspecies problem in relation to human relapsing fever epidemiology. Symposia of the Zoological Society of London. 1962;6:83-156. | Sierra Leone |
| Yorke W, & Blacklock B. Notes on certain animal parasites of domestic stock in Sierra Leone. Annals of Tropical Medicine & Parasitology. 1915;9:413-20. | Sierra Leone |
| Whitaker Jr JO, & Matthysse JG. Records of some ectoparasites from Nigeria. Entomological News. 1982;93:95-102. | Nigeria |
| Williams RW, Causey OR, & Kemp GE. Ixodid ticks from domestic livestock in Ibadan, Nigeria as carriers of viral agents. Journal of Medical Entomology. 1972;9:443-5. | Nigeria |
| Younsi H, Fares W, Cherni S, Dachraoui K, Barhoumi W, Najjar C, et al. *Ixodes inopinatus* and *Ixodes ricinus* (Acari: Ixodidae) are sympatric ticks in North Africa. Journal of Medical Entomology. 2020;57:952-6. | Tunisia |
| Younsi H, Postic D, Baranton G, & Bouattour A. High prevalence of *Borrelia lusitaniae* in *Ixodes ricinus* ticks in Tunisia. European Journal of Epidemiology. 2001;17:53-6. | Tunisia |
| Yousfi-Monod R, Aeschlimann A, & Derscheid JM. 3 infections by trypanosomes observed in *Hyalomma detritum*, *Ixodes ricinus* and *Rhipicephalus sanguineus* (Acarina: Ixodidae). Schweiz Archiv fur Tierheilkunde.1986;128:243-54. | Algeria |
| Yousfi-Monod R. Annual evolution of the sex ratio of *Rhipicephalus sanguineus* (Acarina, Ixodidae) in an urban area of western Algeria. Acarologia. 1985;26:361-5. | Algeria |
| Zeller HG, Cornet JP, & Camicas JL. Experimental transmission of Crimean-Congo hemorrhagic fever virus by west African wild ground-feeding birds to Hyalomma marginatum rufipes ticks. The American Journal of Tropical Medicine and Hygiene. 1994;50:676-81 | Senegal |
| Zeller HG, Cornet JP, Diop A, & Camicas JL. Crimean—Congo hemorrhagic fever in ticks (*Acari: Ixodidae*) and ruminants: Field observations of an epizootic in Bandia, Senegal (1989–1992). Journal of Medical Entomology. 1997;34:511-6. | Senegal |
| Zeroual F, Bitam I, Ouchene N, Leulmi H, Aouadi A, & Benakhla A. Identification and seasonal dynamics of ticks on wild boar (*Sus scrofa*) in the extreme North-east of Algeria. Bulletin de la Société Zoologique de France. 2014;139:245-53. | Algeria |
| Zhioua E, Bouattour A, Hu CM, Gharbi M, Aeschliman A, Ginsberg HS, et al. Infection of *Ixodes ricinus* (Acari: Ixodidae) by *Borrelia burgdorferi* sensu lato in North Africa. Journal of Medical Entomology. 1999;36:216-8. | Tunisia |
| Zhioua E, Gern L, & Aeschlimann A. Isolement d'un spirochète à partir d'*Ixodes ricinus* de Tunisie. Bulletin de la Société française de parasitologie. 1989;7:107-10. | Tunisia |
| Ziam H, Saidani K, & Aissi M. Prevalence of bovine piroplasmosis and anaplasmosis in North-central Algeria. Scientia Parasitologica. 2017;18:7-15. | Algeria |
