# Supplementary figures and images for: A compilation of ticks and tick-borne pathogen distributions in seven countries within North and West Africa from 1901 to 2022: a systematic literature review
Source: Parasit Vectors. 2025 Dec 4;19:13. doi: 10.1186/s13071-025-07153-8 (PMC12781586; doi:10.1186/s13071-025-07153-8)

Number of Citations by Decade and Country

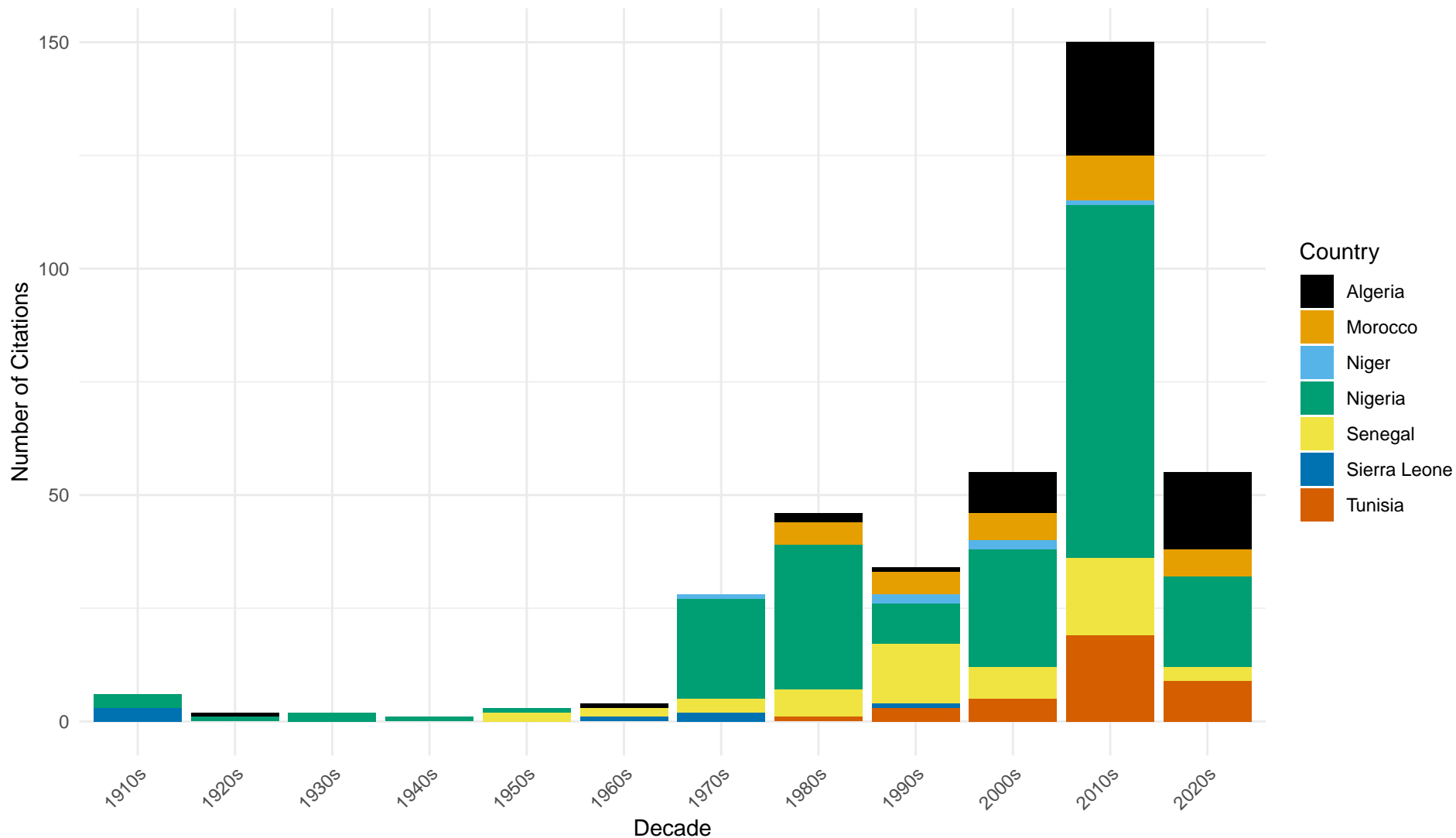

Supplement: Supplementary file 4 — Additional file 4. Timeline of Publications Over Decades.pdf [file 13071_2025_7153_MOESM4_ESM.pdf]
